# Bile salt hydrolases shape the bile acid landscape and restrict *Clostridioides difficile* growth in the murine gut

**DOI:** 10.1038/s41564-023-01337-7

**Published:** 2023-03-13

**Authors:** Matthew H. Foley, Morgan E. Walker, Allison K. Stewart, Sarah O’Flaherty, Emily C. Gentry, Shakshi Patel, Violet V. Beaty, Garrison Allen, Meichen Pan, Joshua B. Simpson, Caroline Perkins, Molly E. Vanhoy, Michael K. Dougherty, Sarah K. McGill, Ajay S. Gulati, Pieter C. Dorrestein, Erin S. Baker, Matthew R. Redinbo, Rodolphe Barrangou, Casey M. Theriot

**Affiliations:** 1 Department of Pathobiology and Population Health, College of Veterinary Medicine, North Carolina State University, Raleigh, NC USA; 2 Department of Food, Bioprocessing and Nutrition Sciences, North Carolina State University, Raleigh, NC USA; 3 Department of Chemistry, University of North Carolina at Chapel Hill, Chapel Hill, NC USA; 4 Department of Chemistry, North Carolina State University, Raleigh, NC USA; 5 Skaggs School of Pharmacy and Pharmaceutical Sciences, University of California San Diego, San Diego, CA USA; 6 Collaborative Mass Spectrometry Innovation Center, Skaggs School of Pharmacy and Pharmaceutical Sciences, University of California San Diego, La Jolla, CA USA; 7 Department of Pediatrics, Division of Gastroenterology and Hepatology, University of North Carolina at Chapel Hill, Chapel Hill, NC USA; 8 Department of Pathology and Laboratory Medicine, University of North Carolina at Chapel Hill, Chapel Hill, NC USA; 9 Departments of Biochemistry and Biophysics, and Microbiology and Immunology, and the Integrated Program in Biological and Genome Sciences, University of North Carolina at Chapel Hill, Chapel Hill, NC USA

**Keywords:** Hydrolases, Pathogens, Metabolomics

## Abstract

Bile acids (BAs) mediate the crosstalk between human and microbial cells and influence diseases including *Clostridioides difficile* infection (CDI). While bile salt hydrolases (BSHs) shape the BA pool by deconjugating conjugated BAs, the basis for their substrate selectivity and impact on *C. difficile* remain elusive. Here we survey the diversity of BSHs in the gut commensals Lactobacillaceae, which are commonly used as probiotics, and other members of the human gut microbiome. We structurally pinpoint a loop that predicts BSH preferences for either glycine or taurine substrates. BSHs with varying specificities were shown to restrict *C. difficile* spore germination and growth in vitro and colonization in pre-clinical in vivo models of CDI. Furthermore, BSHs reshape the pool of microbial conjugated bile acids (MCBAs) in the murine gut, and these MCBAs can further restrict *C. difficile* virulence in vitro. The recognition of conjugated BAs by BSHs defines the resulting BA pool, including the expansive MCBAs. This work provides insights into the structural basis of BSH mechanisms that shape the BA landscape and promote colonization resistance against *C. difficile*.

## Abstract (web summary)

Bile salt hydrolases encoded by the gut microbiome shape the bile acid pool, including microbial conjugated bile acids, which impact *Clostridioides difficile* infection in the murine gut.

## Main

Bile acids (BAs) are host-synthesized and microbial-derived metabolites that support intestinal health and homoeostasis by providing a scaffold for host-microbiome crosstalk and adaptation^1,2^. Host-encoded BA receptors recognize BAs as signalling molecules to regulate host immunity^3^, metabolism^4^ and circadian rhythms^5,6^. BAs also shape the microbiota, where specific taxa exhibiting resistance to bile stress harbour mechanisms to survive BA exposure^7,8^. The influence of BAs on the microbiota is reciprocated by the microbial transformation of BAs to establish the chemical complexity of the intestinal BA pool^9^. The resulting mosaicism is a hallmark of host health and is dependent on the collective activity of microbiome-encoded BA-altering enzymes^10^.

**Fig. 1:**
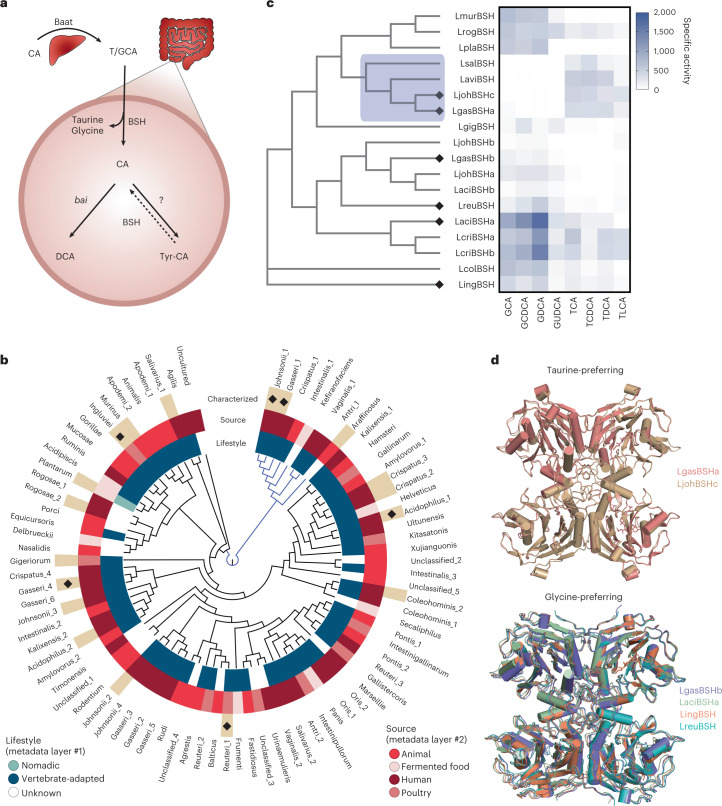
Lactobacillaceae BSHs have distinct substrate preferences. **a**, Overview of BA metabolism using CA as an example. BAs are synthesized and conjugated with a taurine or glycine (T/GCA) by the liver enzyme Baat. Conjugated BAs are deconjugated by BSHs, allowing transformations such as 7α-dehydroxylation encoded by the *bai* operon to generate secondary BAs such as DCA. Deconjugated BAs can be reconjugated to produce MCBAs such as Tyr-CA. **b**, The Lactobacillaceae BSH phylogenetic tree was assembled from 84 BSH clusters. The two major clades are distinguished by black and blue branches. Each cluster name indicates the BSH-encoding species, with the inner metadata layers indicating lifestyle and isolation source^82^. Beige wedges in the outer metadata layer indicate the BSHs cloned for characterization. **c**, Heat map of BSH activity across conjugated BAs. The clade highlighted in blue displays preference for taurine-conjugated BAs. Diamonds in **b** and **c** indicate BSH crystal structures presented here. Values represent mean specific activity (*n* = 3–4). Phylogenetic relationships between BSHs are based on amino acid sequences. **d**, Superposition of the taurine and glycine-preferring BSH enzyme tetramer crystal structures presented here. Source data

Bile salt hydrolases (BSHs) comprise a diverse family of microbial enzymes that catalyse critical BA transformation^11^. Primary conjugated BAs synthesized in the liver, such as the tauro- and glycoconjugates of cholic acid (TCA, GCA), are deconjugated by BSHs to generate cholic acid (CA) (Fig. 1a; BA names and abbreviations are summarized in Supplementary Fig. 1). This vanguard reaction acts as a ‘gatekeeper’ for subsequent BA transformations that turn primary BAs into secondary BAs (that is, deoxycholic acid (DCA) formed from the 7α-dehydroxylation of CA by bacteria that encode the *bai* operon)^1,11^.

Dysregulation of BA biosynthesis and BSH activity has been directly implicated in obesity^12^, cancer^13^, inflammatory bowel disease^3^ and colonization resistance against pathogens including *Clostridioides difficile*^14–18^. *C. difficile* infection (CDI) is an important public health problem that is difficult to treat with conventional antibiotics^19^. Even after a course of vancomycin or fidaxomicin, there remains a high recurrence rate (~30%)^20,21^, making a faecal microbiota transplant (FMT) the last line of treatment^22^. Although FMTs can treat recurrent CDI (rCDI), the long-term consequences remain unclear, making a targeted therapeutic approach appealing for safety and efficacy reasons. Susceptibility to CDI is predominantly driven by antibiotic-induced perturbations to the intestinal microbiota resulting in a loss of colonization resistance^23,24^. Strong evidence suggests that microbial BA metabolism is an important mechanism of colonization resistance against *C. difficile*^25^, as antibiotic usage depletes BSHs, increasing conjugated BAs and decreasing secondary BAs^17^.

BAs govern fundamental aspects of the *C. difficile* pathogenic life cycle: TCA acts as a germinant for *C. difficile* spores^26^, whereas chenodeoxycholic acid (CDCA) can inhibit germination and growth^27^. Most studies have focused on the production of deconjugated secondary BAs as inhibitors of *C. difficile* pathogenesis, while relatively few have examined the contributions of BSH activity alone^28^. The therapeutic potential of BSHs has been proposed, but gaps in our knowledge of their pleiotropic effects on intestinal biology and their exact mechanism of action have hampered their development^29,30^. Additionally, the recent discovery of microbial conjugated bile acids (MCBAs) has considerably increased the complexity of the BA pool and altered the paradigm of BSH-BA biology^31–33^, warranting new investigations into BSH–substrate relationships (Fig. 1a).

Here we examine the effect of BSH enzymes on *C. difficile* pathogenesis using the Lactobacillaceae as a model gut microbial family encoding a highly active set of BSHs^34–36^. First, we identify a 3-residue selectivity loop that is predictive of the glycine vs taurine substrate preferences of BSH enzymes. Using this structural motif as a guide, we deploy a set of selective BSH enzymes with differing substrate preferences to inhibit *C. difficile* spore germination and colonization in pre-clinical models. Finally, we further establish an additional function for BSHs as enzymes able to shape the MCBA pool in vivo. Together, these data reveal the structural underpinnings of BSH activity that shape the bile acid landscape and can govern *C. difficile* biology.

## Results

### Surveying Lactobacillaceae BSH diversity

To map the range of BSHs harboured by Lactobacillaceae, 3,712 genomes from 274 Lactobacillaceae were assembled to comprehensively define the diversity of the enzyme within this genus (Supplementary Table 1)^37^. Highly similar BSH sequence (>95% amino acid identity) were grouped into 84 clusters and a representative sequence from each cluster was used to construct a phylogenetic tree. Nearly all BSH clusters were identified from vertebrate-adapted species and ~40% were human-associated, underscoring their role in host-microbiota symbiosis (Fig. 1b and Supplementary Table 1).

**Fig. 2:**
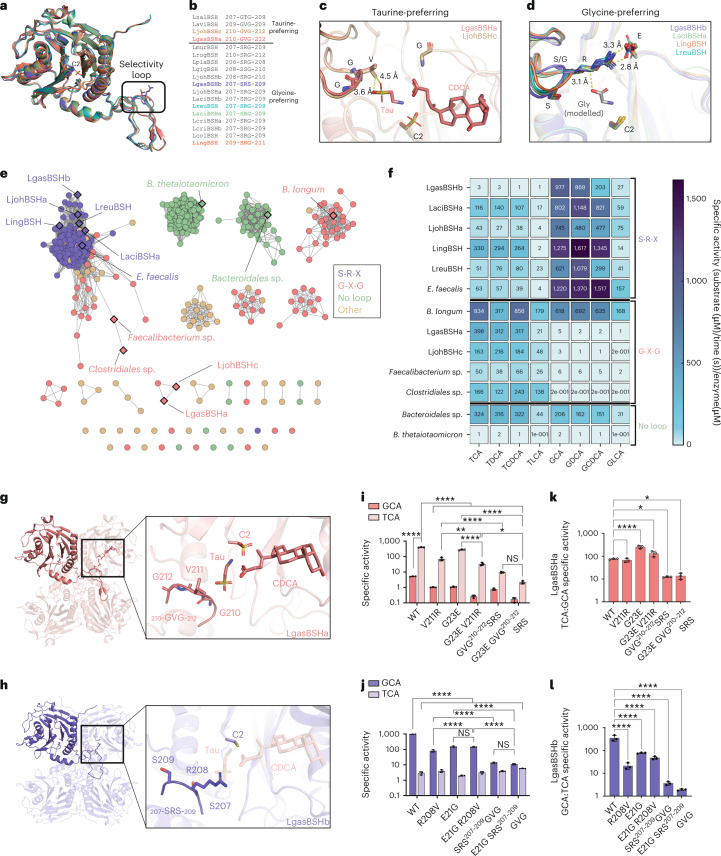
BSH selectivity loop sequence predicts substrate preference. **a**, Superimposed monomers of six BSH structures highlighting the substrate preference loop and coloured as indicated in **b**. **b**, Sequence alignment of the selectivity loop region of Lactobacillaceae BSHs. Resolved structures are uniquely coloured, with a constant colouring scheme throughout the figure. Taurine-preferring enzymes exhibit a G-V/T-G motif, while glycine-preferring enzymes maintain an S-R-G/S motif. **c**,**d**, Superposition of the active sites of either two taurine-preferring (**c**) or four glycine-preferring (**d**) BSH structures reveals a consistent active site architecture that accommodates taurine or glycine, respectively. **e**, Sequence similarity network (SSN) of BSHs reveals clustering by selectivity loop sequence. Lines connecting BSHs indicate relatedness. Bolded diamonds represent proteins selected for activity characterization. Sequences (689) were clustered by 95% sequence identity to yield 654 unique nodes (circles). **f**, Heat map of specific activities of a panel of BSH enzymes with 8 BA substrates shows that substrate specificity is predicted by selectivity loop sequence. Values in boxes represent mean specific activity from *n* = 3 replicates. **g**, The LgasBSHa tetramer with the active site shown in the inset, where the catalytic cysteine at position 2, the bound taurine and CDCA compounds, and the GVG swapped into the active site from the neighbouring monomer are highlighted. **h**, The LgasBSHb tetramer with the active site shown in the inset, where the catalytic cysteine at position 2 and the SRS swapped into the active site from the neighbouring monomer are highlighted. The taurine and CDCA ligands from the LgasBSHa structure are also rendered transparent. **i**,**j**, Specific activities for wild-type and mutant forms of LgasBSHa (**i**) and LgasBSHb (**j**) with GCA and TCA. Bars in **i** and **j** are the mean ± s.d. of *n* = 3. Significant differences were tested by two-way ANOVA with Tukey’s multiple comparisons test (NS, not significant; *P < 0.05, **P < 0.01, ****P < 0.0001). **k**,**l**, Ratios of TCA:GCA specific activity for LgasBSHa (**k**) and GCA:TCA specific activity for LgasBSHb (**l**). Bars in **k** and **l** are the mean ± s.d. of *n* = 3. Significant differences were tested by one-way ANOVA with Tukey’s multiple comparisons test. *P < 0.05, ****P < 0.0001. All *P* values are listed in Supplementary Data 1. Source data

To move toward a functional understanding of BSH activity in vivo, 18 diverse BSHs were selected for heterologous expression and purification (Supplementary Table 2 and Fig. 2). BSH activity was screened on a panel of conjugated primary and secondary BAs to comprehensively determine BSH substrate preferences **(**Fig. 1c). We found that Lactobacillaceae BSHs display a clear bias for either glycine or taurine-conjugated BAs. Glycine preferences were prominent, potentially reflecting the increased abundance of glycine-conjugated BAs among vertebrates^38^, whereas taurine specificity is restricted to only a few related BSHs. The glycine-preferring enzyme clade containing LaciBSHa, LcriBSHa and LcriBSHb exhibits the highest activity. Furthermore, the taurine-preferring LaviBSH, LjohBSHc and LgasBSHa (corresponding to the clusters arrafinosis, johnsonii_1 and gasseri_1, respectively) enzymes are all present within a single major clade, while the LsalBSH (cluster salivarius_1) stands alone in a distantly related branch (Fig. 1b,c). This suggests that taurine preference may have evolved twice within Lactobacillaceae. Overall, BSH specialization for glycine or taurine illustrates how lactobacilli may have tailored their metabolism to manage BA exposure.

### The BSH selectivity loop defines substrate preference

**Extended Data Fig. 1:**
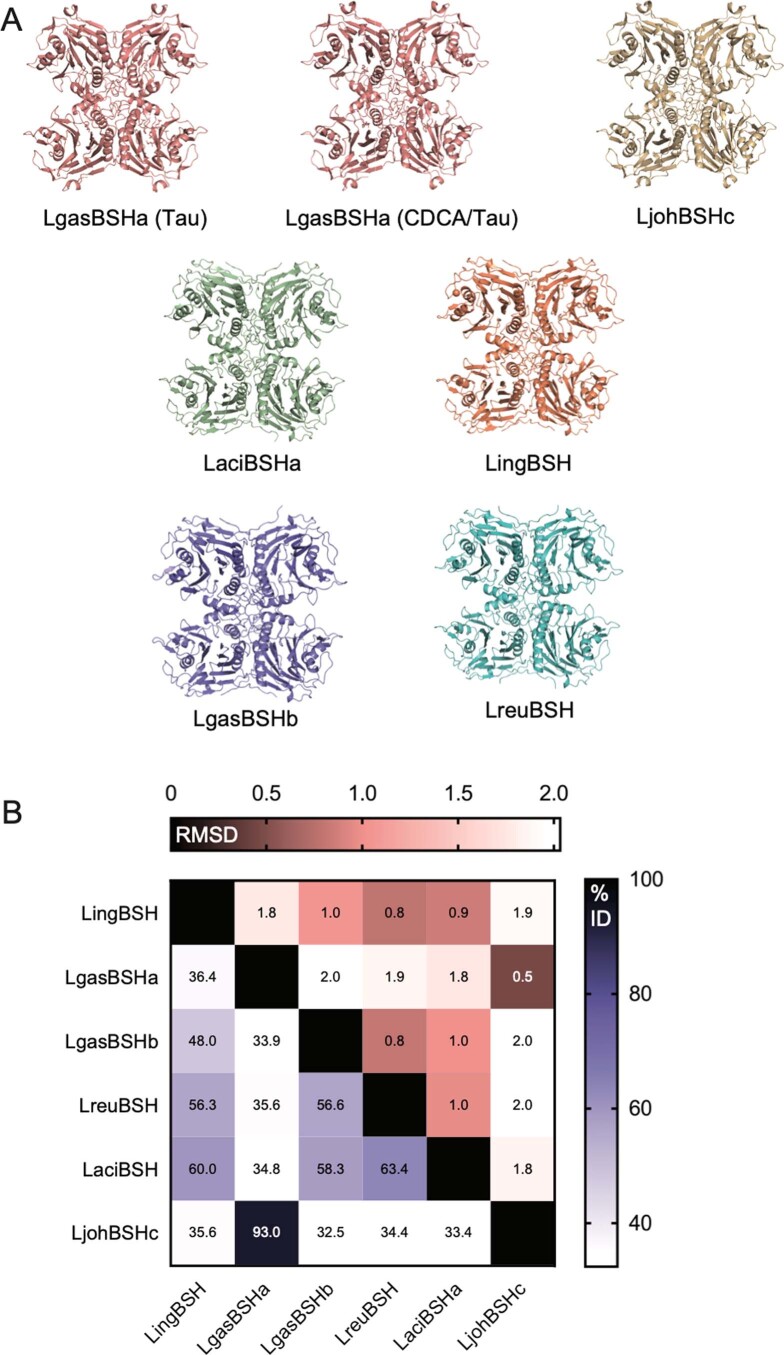
Structural and sequence comparisons of Lactobacillaceae BSHs. (A) Full tetrameric structures of the crystal structures presented here and colored as indicated in Fig. 2B (B) Root-mean square deviation (RMSD) in Å across equivalent Cα positions for the crystal structures presented, as well as % sequence identity values.

To examine the molecular foundations of BSH substrate specificity, we determined the crystal structures of six of the Lactobacillaceae BSHs examined above: four glycine-preferring (LgasBSHb, LaciBSHa, LingBSH, LreuBSH) and two taurine-preferring (LgasBSHa, LjohBSHc; Figs. 1d and 2a, Extended Data Fig. 1a and Supplementary Table 3) BSHs. LgasBSHa structures were determined in complexes with taurine and taurine plus CDCA. All enzymes form analogous tetramers of monomers exhibiting the core four-layered αββα Ntn-hydrolase family fold^39^ and 0.5–2.0 Å root mean square deviation values over Cα positions (Fig. 2a and Extended Data Fig. 1b). Notably, each monomer contains a loop that swaps into the active site of a neighbouring monomer within the tetramer and places amino acids proximal to the catalytic residues (Fig. 2a,c,d,g,h). We examined the sequences of these loops and found that taurine-preferring enzymes exclusively contained G-V/T-G (‘G-X-G’) motifs, while glycine-preferring enzymes solely contained S-R-G/S (‘S-R-X’) motifs (Fig. 2b–d and Supplementary Fig. 3)^40^. Thus, we hypothesized that this ‘selectivity loop’ may be predictive of BSH glycine vs taurine conjugate preferences.

To examine the selectivity loop beyond Lactobacillaceae enzymes, we used protein sequence and structure to identify 654 unique BSH sequences from the 9.9 million unique proteins in the Integrated Gene Catalog, a large metagenomic database^41^. A sequence similarity network reveals four selectivity loops: S-R-X, G-X-G, No Loop and Other (Fig. 2e and Supplementary Table 4). ‘No Loop’ proteins (141) lacked the ~20-residue selectivity region, while ‘Other’ proteins^42^ contained motifs unlike those found in the Lactobacillaceae. However, 350 and 87 sequences encoded S-R-X and G-X-G proteins, respectively, indicating that the selectivity loop is present in ~67% of BSH proteins in the gut microbiome. We selected representative S-R-X, G-X-G and No Loop sequences from diverse classes, clusters and taxa, and determined the activity of these purified enzymes with a panel of BA substrates (Fig. 2f). Enzymes within the S-R-X and G-X-G classes demonstrated glycine or taurine selectivity, respectively, while No Loop enzymes processed all substrates on a similar level, probably due to the absence of the selectivity loop. Thus, the selectivity loop sequence is predictive of gut microbial BSH substrate specificity.

To confirm the importance of the selectivity loop, we first created two variant enzymes that swap the central residues in each selectivity loop: a V211R mutant of the taurine-preferring LgasBSHa and a R208V mutant of the glycine-preferring LgasBSHb. We found that both single-swap mutations significantly decreased the specific activities with preferred substrates without significantly impacting the non-preferred substrate (Fig. 2i–l). Second, we noted that R208 in LgasBSHb forms a salt bridge with E21, an anionic residue conserved in all glycine-preferring enzymes examined; in taurine-preferring BSHs, this glutamate is always replaced by glycine (Fig. 2c,d). We examined the activity of LgasBSHa G23E and LgasBSHb E21G variants and found that they decreased activity with the preferred substrate without impacting non-preferred substrate activity (Fig. 2i,j). Third, combining these mutations by adding a G23E V211R salt bridge in LgasBSHa further lowered the preferred activity (Fig. 2i), while removing the salt bridge in LgasBSHb E21G R208V did not further decrease the preferred activity (Fig. 2j). Fourth, we created triple mutants of the selectivity loop sequence (Fig. 2g,h). Both the LgasBSHa-SRS and LgasBSHb-GVG variants exhibited the greatest reduction in specific activities toward their preferred substrates, with little effect on non-preferred substrate processing (Fig. 2i,j). Finally, adding the G/E change to these 3-residue mutations produced no additional effect (Fig. 2i,j). Importantly, without altering secondary structure (Supplementary Fig. 4 and Table 5), the triple mutations significantly decreased substrate selectivity from 76-fold to 14-fold for LgasBSHa and from 353-fold to 2-fold for LgasBSHb (Fig. 2k,l). These data confirm the importance of the selectivity loop for BSH glycine versus taurine conjugate substrate preferences.

### Bile acid conjugation-dependent inhibition of *C. difficile*

**Fig. 3:**
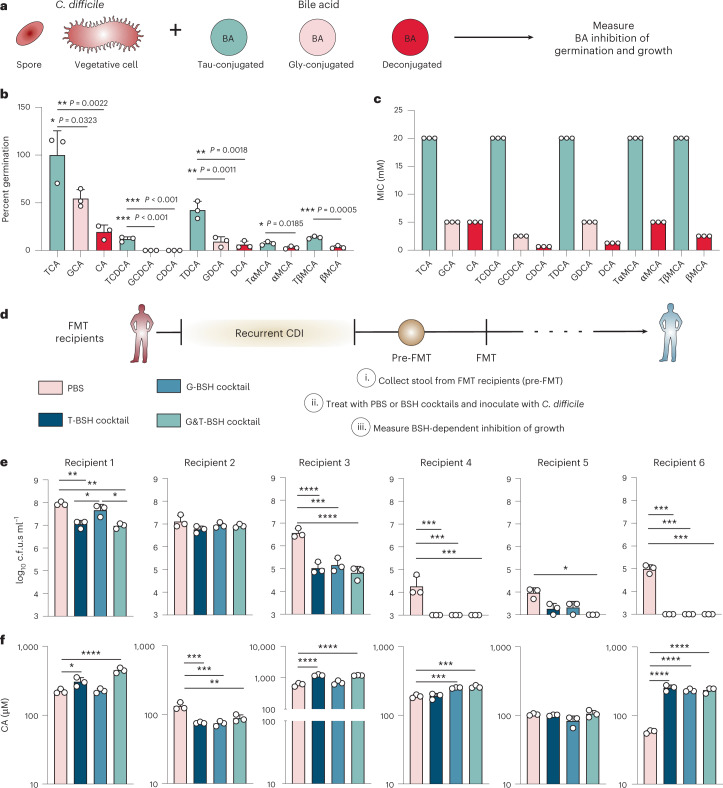
Deconjugated BAs and BSH activity can inhibit *C. difficile*. **a**, Schematic of in vitro BA-dependent inhibition of *C. difficile* germination and growth. **b**, Inhibition of spore germination using various BAs (0.75 mM) in the presence of the germinant TCA (2 mM). Comparisons were only made between BAs that share the same sterol core using one-way ANOVA with Tukey’s multiple comparisons test, except for the αMCA and βMCA BAs which were compared with one-tailed Welch’s *t*-test. **c**, Inhibition of *C. difficile* growth as determined by MIC testing. Bar colours in **b** and **c** are coded consistently on the basis of the BA conjugation indicated in **a**. **d**, Schematic of pre-FMT collection and ex vivo growth. Pre-FMT stool collected from rCDI patient was supplemented with PBS or a BSH cocktail and subsequently inoculated with *C. difficile*. Recipients 1–3 and 4–6 were inoculated with 10^5^ and 10^6^ c.f.u.s ml^−1^, respectively. **e**, *C. difficile* growth measured at 8 h. **f**, Targeted metabolomics showing CA from the samples in **e**. Statistical differences were tested by one-way ANOVA with Sidak’s correction for multiple comparisons. All bars represent mean ± s.d. of *n* = 3 replicates. *P < 0.05, **P < 0.01, ***P < 0.001, ****P < 0.0001. All *P* values are listed in Supplementary Data 1. Source data

**Extended Data Fig. 2:**
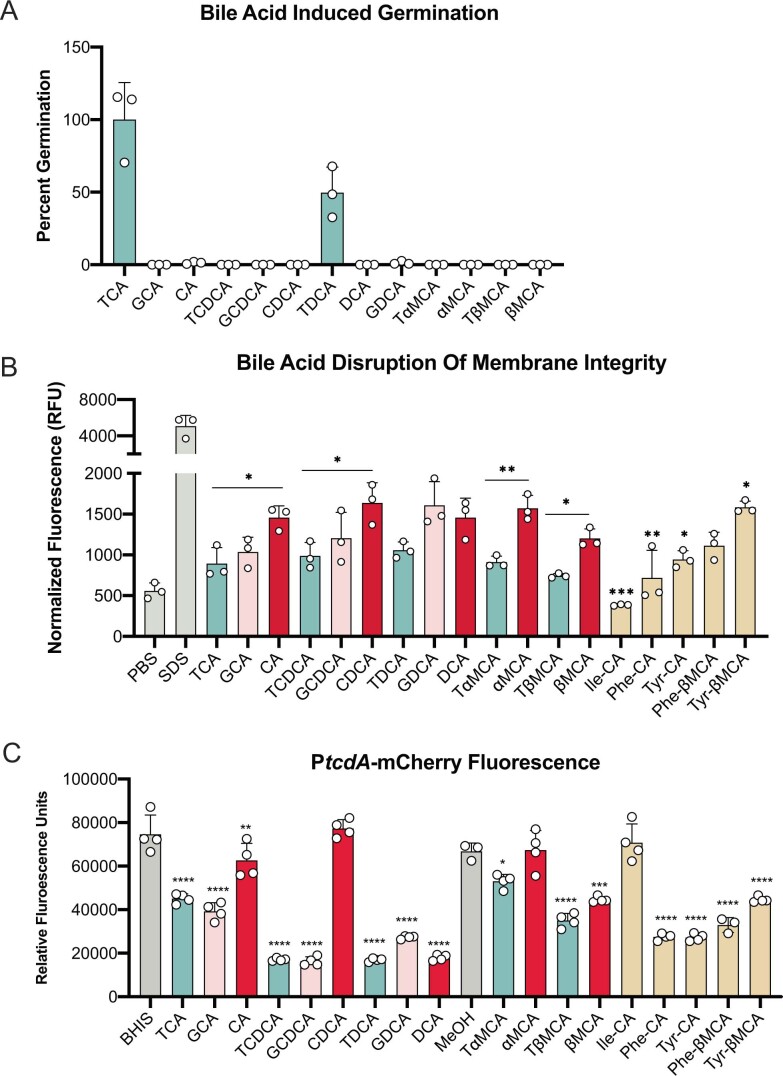
Bile acid impacts on *C. difficile* biology. A) Germination of *C. difficile* carried out using 2 mM BAs as the sole germinant. (B) Propidium iodide staining of exponentially growing *C. difficile* after a 30 min exposure to BAs (n = 3). Bars from A and B represent mean biological replicates (n = 3) ± standard deviation. Conventional BAs and Trp-CDCA concentrations were 0.25x their MIC except the MCBAs which were used at 0.5 mM. The detergent SDS (150 μM) was used as a positive control for detergent-induced membrane damage. Comparisons were only made between BAs that share the same BA sterol core using a one-way ANOVA with Dunnett’s multiple comparisons test except for αMCA and TαMCA which were compared with a one-tailed Welch’s *t* test. (C) BA regulation of *tcdA* expression using a P_*tcdA*_-mCherry reporter^31^. *C. difficile* was grown for 24 in the presence of the conventional BAs and Trp-CDCA at 0.25x their MIC whereas the MCBAs were used at 0.5 mM. Bars represent mean biological replicates (n = 4) ± standard deviation. BAs conditions were compared to BHIS or MeOH (BHIS media with methanol) based on the BA’s solvent using a one-way ANOVA with Dunnett’s multiple comparisons test. Asterisks indicate significant differences (*p < 0.05, **p < 0.01, ***p < 0.001, ****p < 0.0001). All *p* values listed in Supplementary Data 1. Bars are color coded as follows: teal = taurine conjugation, pink = glycine conjugation, red = deconjugated, beige = MCBA, grey = no BA. Source data

After exploring the structural basis for BSH substrate preferences, we wanted to determine how these differential BSH activities impact *C. difficile* since different stages of its life cycle are exquisitely sensitive to different bile acids. First, we measured the ability of many common BAs to induce spore germination alone or inhibit germination in the presence of a germinant TCA (Fig. 3a). While most BAs alone do not induce spore germination in *C. difficile* R20291 (Extended Data Fig. 2a), most BAs tested can inhibit TCA-mediated germination (Fig. 3b). Deconjugated BAs are significantly more inhibitory compared with their taurine-conjugated versions, whereas they are equally or only mildly more inhibitory relative to their glycine-conjugated variants (Fig. 3b). We also assayed the ability of BAs to inhibit *C. difficile* growth, as we hypothesized that the conjugation would determine BA toxicity (Fig. 3c). Taurine conjugates inhibit growth less, while glycine conjugates and deconjugated BAs are much more toxic to *C. difficile*. Deconjugated BAs especially inhibit *C. difficile* growth even at low sub-minimum inhibitory concentrations (MIC) (Supplementary Fig. 5). Deconjugated BAs are weaker acids, making them more likely to be uncharged and disruptive of the bacterial membrane. Indeed, deconjugated BAs are significantly more disruptive to *C. difficile*’s membrane integrity compared with taurine and glycine-conjugated BAs (Extended Data Fig. 2b). However, conjugated BAs may suppress the expression of *C. difficile*’s toxin A (*tcdA*), as demonstrated using its promoter (P_*tcdA*_) fused to an mCherry reporter (Extended Data Fig. 2c)*. C. difficile* toxins mediate inflammation and their decreased expression may ameliorate disease. Thus, the use of BSHs to selectively deconjugate taurine or glycine-conjugated BAs may be able to restrict *C. difficile* in the intestinal tract.

**Extended Data Fig. 3:**
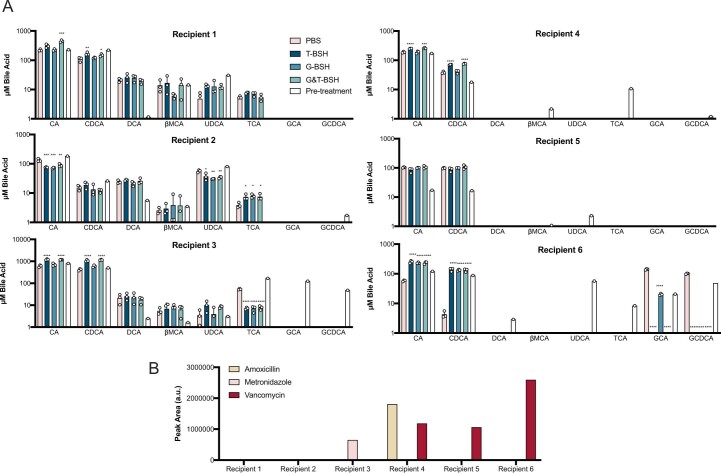
Bile acid metabolomics of BSH-treated pre-FMT stool. (A) Targeted metabolomics showing all detected BAs at 8 h in pre-FMT stool samples. Bars represent mean (n = 3) ± standard deviation. Asterisks indicate significant differences (*p < 0.05, **p < 0.01, ***p < 0.001, ****p < 0.0001) from PBS by one-way ANOVA with Sidak’s correction for multiple comparisons. All *p* values listed in Supplementary Data 1. (B) Untargeted metabolomic analysis of antibiotics detected in pre-FMT fecal samples. Vancomycin was detected in all three samples that were most inhibitory to *C. difficile* even in the PBS control condition. Source data

**Extended Data Fig. 4:**
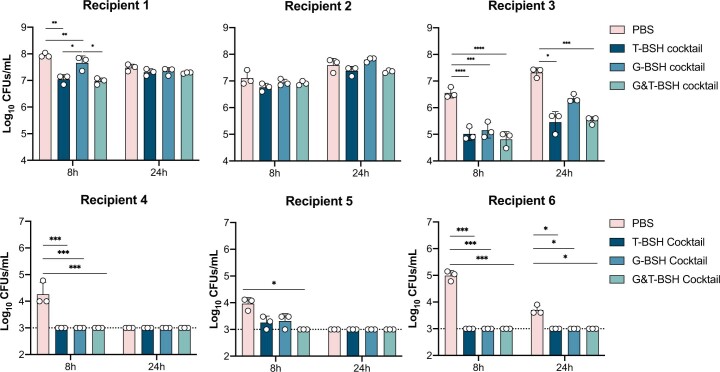
*C. difficile* growth in pre-FMT stool. Growth was measured at 8 and 24 h from an n = 3 replicates. Targeted metabolomics showing the deconjugated BAs CA and CDCA from the samples from. Bars represent mean ± standard deviation. Asterisks indicate significant differences (*p < 0.05, **p < 0.01, ***p < 0.001, ****p < 0.0001) between CFUs by one-way ANOVA with Sidak’s correction for multiple comparisons. All *p* values listed in Supplementary Data 1. Source data

To determine whether BSH activity and selectivity impact *C. difficile* growth, we deployed cocktails of glycine or taurine-preferring BSHs to treat the stool of patients with rCDI. Unlike mice, FMT recipients have a complex mixture of both taurine and glycine-conjugated BAs (Extended Data Fig. 3a). Six rCDI stool samples were collected before each patient receiving an FMT (Fig. 3d). Pre-FMT stool was treated with PBS, a cocktail of taurine-preferring BSHs (T-BSH), glycine-preferring BSHs (G-BSH) or a broadly acting combination (G&T-BSH) before *C. difficile* inoculation to determine whether BSH substrate preference influenced *C. difficile* growth (Fig. 3e and Extended Data Fig. 3a). The BSH cocktails, glycine or taurine alone have no impact on *C. difficile* growth (Supplementary Fig. 6). Despite the pre-FMT stool containing a variety of starting BA pools (Extended Data Fig. 3a), Recipient samples 1, 3, 4 and 6 displayed significant BSH-dependent *C. difficile* inhibition at 8 h and/or 24 h regardless of the cocktail’s substrate selectivity (Fig. 3e and Extended Data Fig. 4). Recipient 5’s stool only showed inhibition when treated with the G&T-BSH cocktails at 8 h and Recipient 2’s stool showed no significant inhibition across treatments at 8 or 24 h (Fig. 3e and Extended Data Fig. 4). Recipients 4, 5 and 6’s stool inhibited *C. difficile* growth probably due to elevated levels of residual vancomycin present in these samples (Extended Data Fig. 3b). Nevertheless, killing by the BSH cocktails was significantly enhanced in these samples. Overall, despite predictable sample heterogeneity across recipients, BSHs appear to effectively hinder *C. difficile* growth, irrespective of their selectivity.

Targeted BA metabolomics was used to validate the activity of the BSH cocktails in stool to examine individual variability between recipients (Fig. 3f and Extended Data Fig. 3a). The stool samples of Recipients 1, 3, 4 and 6 display an increase in deconjugated BAs such as CA primarily in response to the BSH cocktails, thereby supporting the idea that deconjugated BAs can inhibit *C. difficile* (Extended Data Fig. 3a). Recipient 3’s high concentration of BAs pre-treatment provides an explanation for the robust inhibition observed (Fig. 3e and Extended Data Fig. 3a). Treatment of Recipient 5’s stool with BSHs does not improve deconjugation, suggesting that there may be some basal BSH activity in this sample. Interestingly, Recipient 2 does not display an increase in deconjugated BAs from any BSH treatment, presumably due to the absence of conjugated BAs pre-treatment (Extended Data Fig. 3a). Taken together, BSHs can inhibit *C. difficile* growth in pre-FMT human faecal samples via deconjugation of conjugated bile acids.

### BSH activity inhibits *C. difficile* spore germination and growth ex vivo

**Fig. 4:**
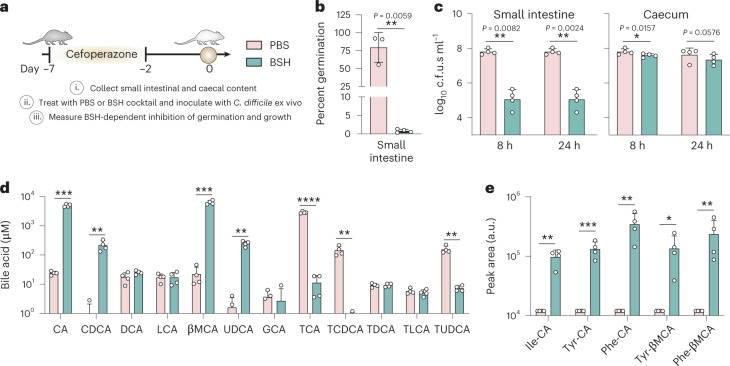
BSHs govern *C. difficile* spore germination and growth along the murine intestinal tract. **a**, Schematic of BSH-dependent inhibition of ex vivo *C. difficile* germination and growth. Intestinal contents collected from cefoperazone-treated mice were supplemented with a cocktail of BSHs and then inoculated with *C. difficile* spores or cells to measure germination or growth, respectively. **b**, Germination of *C. difficile* spores in murine small intestinal contents. Contents were treated with PBS or a BSH cocktail for 8 h before inoculation. **c**, Growth of *C. difficile* at 8 and 24 h in murine intestinal contents. Contents were treated with PBS or a BSH cocktail and inoculated simultaneously. Statistical differences were tested by one-tailed ratio paired *t*-test. **d**,**e**, Targeted metabolomics showing the BAs identified in the day 0 small intestinal samples from **c**. Statistical differences were assessed by one-tailed *t*-test. Bars represent mean ± s.d. (*n* = 4). *P < 0.05, **P < 0.01, ***P < 0.001, ****P < 0.0001. All *P* values are listed in Supplementary Data 1. Source data

**Extended Data Fig. 5:**
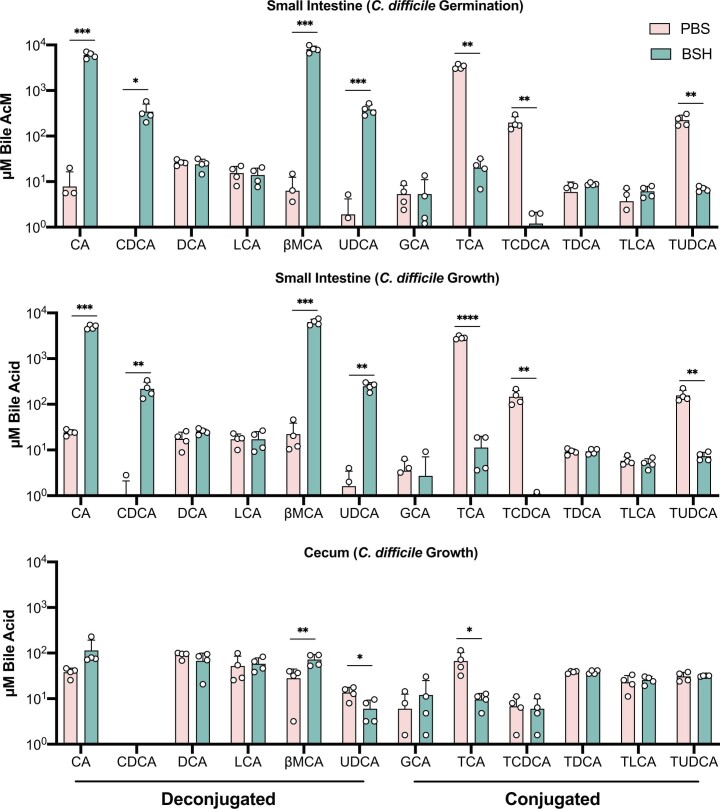
Bile acid metabolomics of *C. difficile* spore germination and growth in mouse intestinal content. Asterisks indicate significant differences (*p < 0.05, **p < 0.01, ***p < 0.001, ****p < 0.0001) between treatments by ratio paired one-tailed *t* test. Bars represent mean ± standard deviation from n = 4 samples. All *p* values listed in Supplementary Data 1. Source data

The observation that BSH treatment can inhibit *C. difficile* in clinically relevant human samples is encouraging but given the difficulty of controlling for variation across stool samples, we sought to understand how BSHs process BAs in the more reproducible murine intestinal tract. *C. difficile* initiates infection when spores germinate in the small intestine then progress to the large intestine where most toxin production and disease occurs. To examine the contribution of BSH activity to colonization resistance along the intestinal tract, we collected murine small intestinal and caecal contents from cefoperazone-treated mice at several times post-antibiotic treatment. Content was then supplemented with PBS or the T-BSH cocktail, which can optimally act on the murine BA pool dominated by taurine-conjugated BAs (Fig. 4a). The treated contents were then inoculated with 10^5^ spores or colony-forming units (c.f.u.s) ml^−1^ to measure ex vivo spore germination and growth, respectively. The addition of the BSH cocktail significantly inhibits *C. difficile* spore germination in the small intestinal contents of all mice and suppresses growth at 8 and 24 h (Fig. 4b,c). Inhibition of growth is more subtle in the caecum, probably due to the lower concentration of BAs in this environment (Fig. 4d and Extended Data Fig. 5). Targeted metabolomics was used to corroborate the BSH cocktail’s effect on the murine BA pool. Small intestinal samples from day 0 treated with the BSH cocktail display a significant increase in deconjugated BAs (CA, CDCA, βMCA and UDCA) and a decrease in the taurine-conjugated forms of the same BAs. In the caecum, only βMCA was significantly deconjugated (Fig. 4d and Extended Data Fig. 5).

Aside from the increase in deconjugated BAs, the most striking difference in the BA pool is the unexpected increase in the MCBAs Ile-CA, Phe-CA, Tyr-CA, Phe-βMCA and Tyr-βMCA in the BSH-treated small intestinal samples (Fig. 4e). An array of intestinal bacteria can catalyse the conjugation of BAs to a variety of amino acids through an unknown mechanism (Fig. 1a)^31,32^, although no evidence exists at this time that MCBAs can be deconjugated or impact *C. difficile*^31^. Given the increased CA and βMCA in the BSH-treated samples, these BAs may be subsequently reconjugated to produce the MCBAs observed.

### BSHs shape the MCBA pool and inhibit *C. difficile* in vivo

**Fig. 5:**
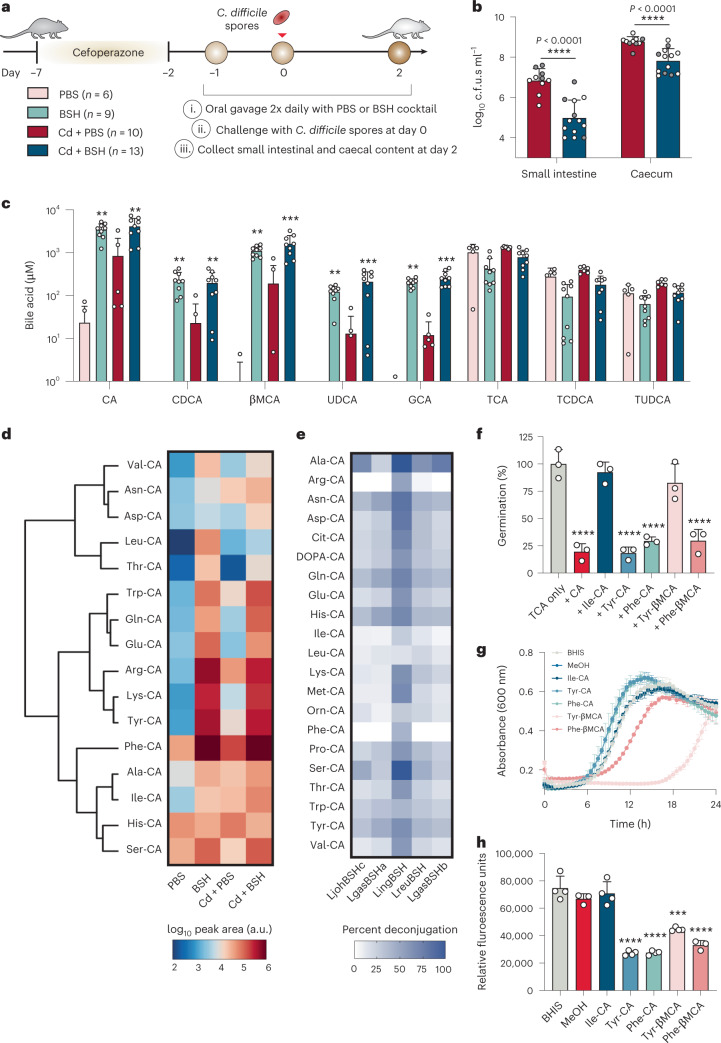
BSHs inhibit *C. difficile* and enrich the MCBA pool in vivo. **a**, Schematic of BSH delivery in a mouse model of CDI. The colour code in this legend corresponds to the test conditions in **b** and **c**. **b**, *C. difficile* c.f.u.s in infected mouse intestinal contents. These data are pooled and the white and grey dots represent separate independent experiments. Bars represent mean ± s.d. (*n* = 10 and 13 for Cd + PBS and Cd + BSH, respectively). Statistical differences were assessed by one-tailed Mann-Whitney test. **c**, Targeted metabolomics showing small intestinal BAs from mice in **b**. Statistical differences were tested by Kruskal-Wallis with Dunn’s test for multiple comparisons. **d**, Targeted metabolomic heat map showing small intestinal MCBAs identified from mice in **b**. MCBAs are organized by unsupervised hierarchical clustering. MCBA abundances are represented as colours corresponding to log_10_-transformed peak areas. **e**, Heat map of select BSH activity across all MCBA pools. Values represent the mean from *n* = 3 replicates of % deconjugation after 5 min. **f**, Inhibition of *C. difficile* germination with MCBAs (0.75 mM) in the presence of the germinant TCA (2 mM). Statistical differences were tested by one-way ANOVA with Dunnett’s test for multiple comparisons. **g**, Growth of *C. difficile* in the presence of 0.75 mM MCBAs. **h**, MCBA regulation of toxin expression using a P*tcdA*-mCherry reporter. Statistical differences were assessed by one-way ANOVA with Dunnett’s test for multiple comparisons. Data in **f**–**h** represent mean ± s.d. (*n* = 3–4). *P < 0.05, **P < 0.01, ***P < 0.001, ****P < 0.0001. All *P* values are listed in Supplementary Data 1. Source data

To further interrogate the role of BSHs in establishing colonization resistance against *C. difficile* and the production of MCBAs in vivo, we leveraged the cefoperazone-treated mouse model of CDI in which the T-BSH cocktail was orally delivered to mice twice daily before *C. difficile* spore challenge (Fig. 5a). Akin to the ex vivo results in Fig. 4, *C. difficile* growth was significantly inhibited in both the small intestine and the caecum of mice that received the BSH cocktail, although this did not alter clinical signs of disease or weight loss (Fig. 5b and Supplementary Fig. 7). Furthermore, targeted metabolomics of those samples demonstrated that the BSH cocktail remained active in vivo and generated an increase in deconjugated BAs despite being orally delivered (Fig. 5c). CDI alone resulted in elevated BA deconjugation in mice. The *C. difficile* R20291 strain did not exhibit BSH activity presumably due to dynamic changes in the gut microbiota from CDI.

**Extended Data Fig. 6:**
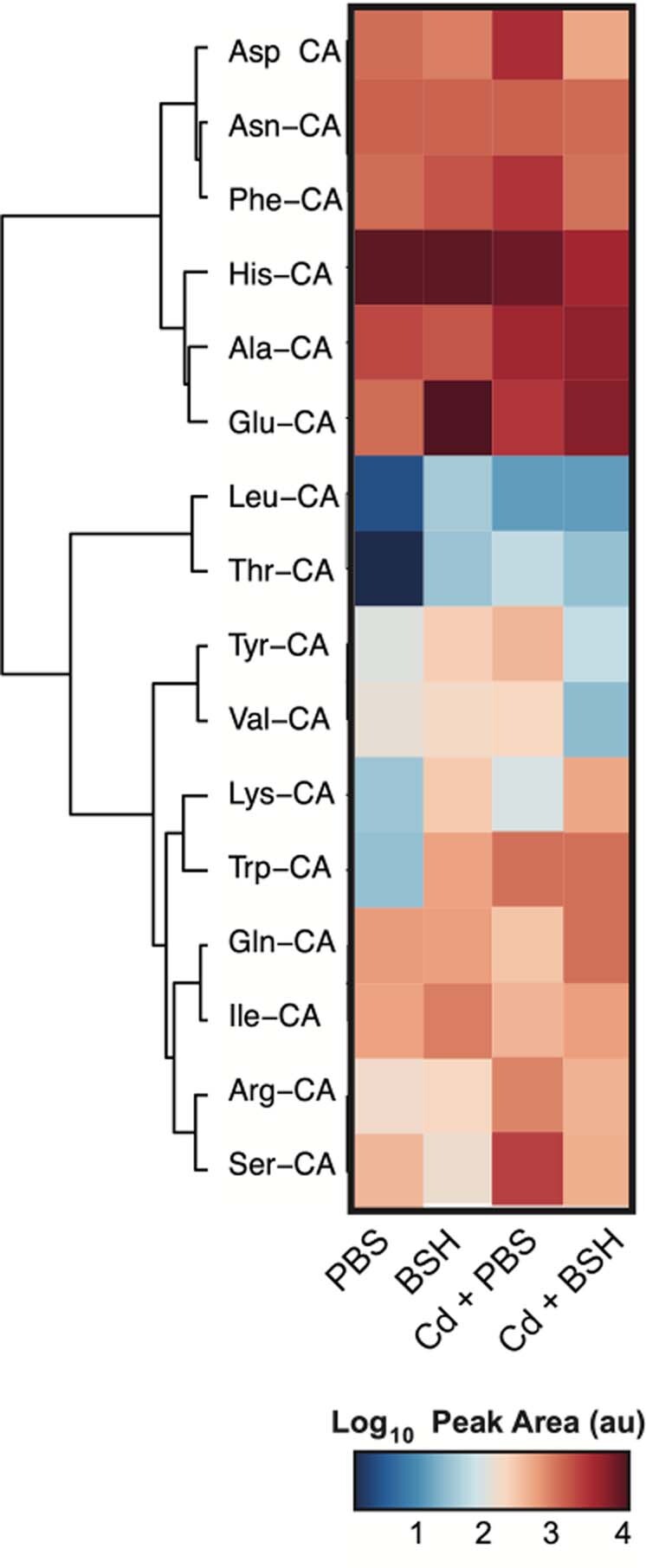
Cecal MCBA pool in BSH treated mice. Targeted metabolomics showing cecal MCBAs identified from mice in Fig. 5B. MCBAs have been Log_10_ transformed and are organized by unsupervised hierarchical clustering. Source data

Given the high concentration of deconjugated BAs generated in the BSH-treated mice (CA, CDCA, βMCA and UDCA), we hypothesized that a set of MCBAs similar to those seen in Fig. 4e would be produced in vivo. Indeed, a large and diverse set of MCBAs was observed, with Ile-CA, Tyr-CA, Phe-CA, Arg-CA and Lys-CA being most prominent (Fig. 5d and Extended Data Fig. 6). Together, these results show that BSHs are actively reshaping the MCBA pool.

**Extended Data Fig. 7:**
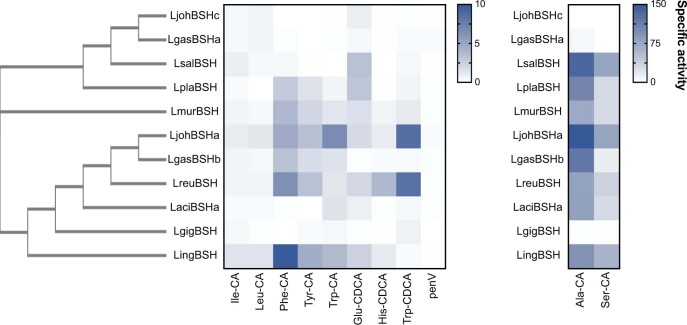
BSH activity with pure MCBAs. Heatmaps of BSH specific activity using pure MCBAs. Values represent mean activity (n = 3). BSHs were arranged into a phylogeny based on their amino acid sequence. The ability to process penV was included as a potential BSH substrate. Source data

**Extended Data Fig. 8:**
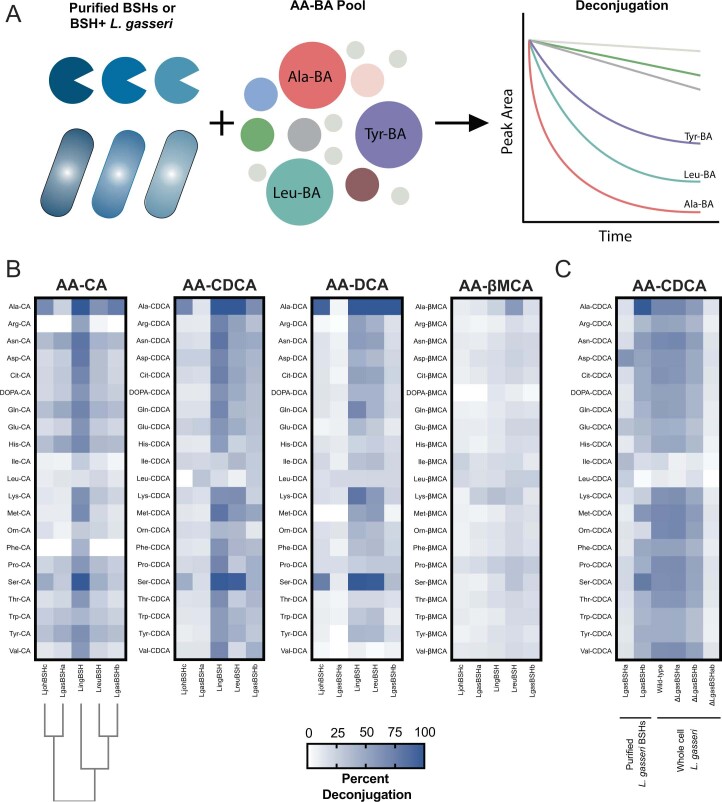
Broadly surveying BSH processing of MCBAs. (A) Schematic of BSH reactions with pooled MCBAs. Individual BSHs were reacted with pooled BA over an hour. Reactions were halted and BA abundance was quantified using LC-IMS-MS targeted BA metabolomics to measure deconjugation over time. (B) A heatmap of BSH activity across all MCBA pools. Values represent the mean from n = 3 replicates of % deconjugation after 5 minutes, which was calculated by measuring the abundance of an individual BA at 0 m and 5 m to quantify the proportion of substrate deconjugated. (C) A heatmap of *L. gasseri* BSH activity after 1 h. Source data

**Extended Data Fig. 9:**
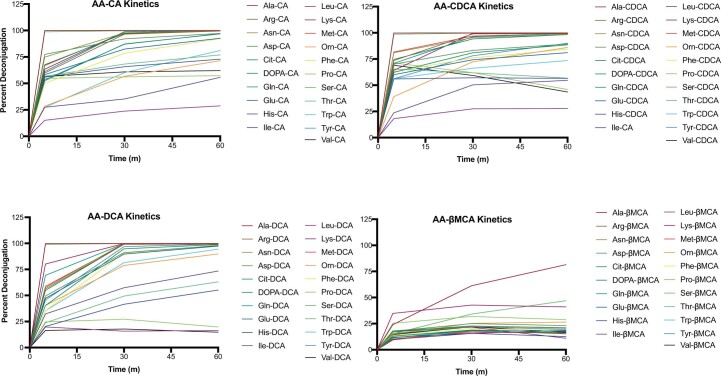
LingBSH deconjugation kinetics of MCBAs. LingBSH-catalyzed deconjugation of MCBAs at 5, 30, and 60 min. Lines are plotted from mean from a n = 3 replicates. Source data

To determine whether BSHs can process or display selectivity for specific MCBAs, we screened a larger set of individual BSHs and MCBAs (Extended Data Fig. 7). While activity with MCBAs is relatively low compared with conventional BAs, some BSHs such as LingBSH and LreuBSH exhibit appreciable activity. To examine the recalcitrance of MCBAs more broadly, we monitored the deconjugation kinetics of 5 BSHs that had previously exhibited assorted substrate preferences (LingBSH, LgasBSHa, LgasBSHb, LjohBSHc and LreuBSH) (Extended Data Fig. 8a). BSHs were incubated with MCBA pools (AA-CA, AA-CDCA, AA-DCA and AA-βMCA) that consist of one BA (CA, CDCA, DCA or βMCA) conjugated to 22 different amino acids, excluding taurine and glycine^33^, and initial BA deconjugation was quantified using liquid chromatography-ion mobility spectrometry-mass spectrometry (LC–IMS–MS) (Fig. 5e and Extended Data Fig. 8b). LingBSH displays exceptional activity, suggesting that some BSHs may be uniquely adapted to process this expanded repertoire of BAs (Fig. 5e, and Extended Data Figs. 8b and 9). Ala and Ser-conjugated BAs are most efficiently processed, and these observations were validated using purified Ala-CA and Ser-CA demonstrating that their susceptibility to deconjugation is akin to GCA and TCA (Extended Data Fig. 7). Ala and Ser are the smallest amino acids next to Gly, suggesting that the steric constraints of the active site could dictate MCBA deconjugation (Supplementary Fig. 8). Notably, amine-containing amino acids such as Lys, Gln and Asn are enriched and among the most efficiently processed conjugates (Supplementary Fig. 9).

Additionally, we examined whether MCBA processing would occur in BSH-expressing *Lactobacillus gasseri* cells. Wild-type, Δ*bshA*, Δ*bshB* and Δ*bshAB L. gasseri* were incubated with the AA-CDCA pool, and the resultant supernatants were examined to detect deconjugation. We note that the Δ*bshAB* double mutant displays basal deconjugation potentially arising from non-specific hydrolases or cell sequestration of supernatant BAs (Extended Data Fig. 8c). However, individual BSH activities and substrate preferences were comparably maintained in vivo, demonstrating that BSH-expressing bacteria are capable of MCBA deconjugation and that they do so on the basis of the enzymatic preferences of the BSHs they encode.

**Extended Data Fig. 10:**
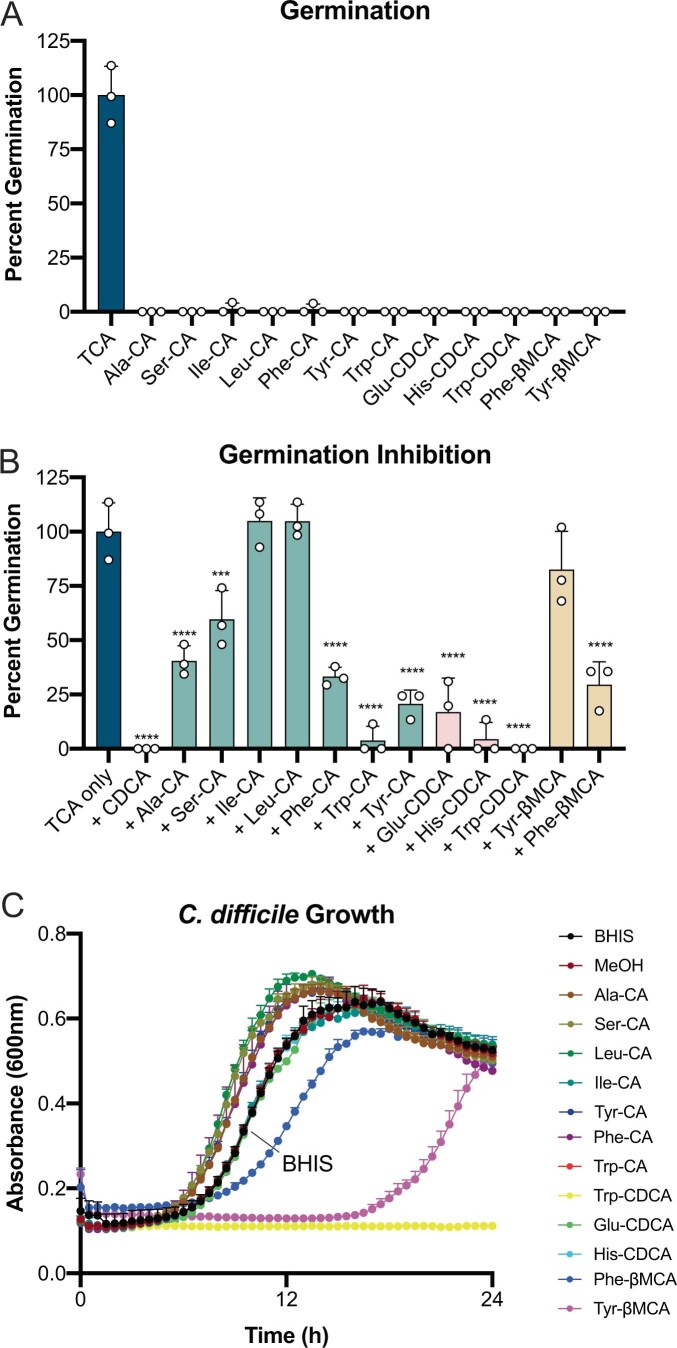
*C. difficile* growth and spore germination with MCBAs. Germination of *C. difficile* was carried out using 2 mM BAs. (B) Germination inhibition with the germinant TCA (2 mM) was carried out with 0.75 mM MCBA. Bars from A and B represent mean (n = 3) ± standard deviation. Bars are color coded as follows: teal = taurine conjugation, pink = glycine conjugation, red = deconjugated, beige = MCBA, grey = no BA. (C) Growth was carried out in the presence of 2 mM BAs in triplicate. Data represent mean ± standard deviation. Trp-CDCA inhibited growth at this concentration and its MIC was determined to be 0.5 mM. Asterisks indicate significant differences (***p < 0.001, ****p < 0.0001) from TCA only by one-way ANOVA with Sidak’s correction for multiple comparisons. Germination of *C. difficile* was carried out using 2 mM BAs. (B) Germination inhibition with the germinant TCA (2 mM) was carried out with 0.75 mM MCBA. Bars from A and B represent mean (n = 3) ± standard deviation. Bars are color coded as follows: teal = taurine conjugation, pink = glycine conjugation, red = deconjugated, beige = MCBA, grey = no BA. (C) Growth was carried out in the presence of 2 mM BAs in triplicate. Data represent mean ± standard deviation. Trp-CDCA inhibited growth at this concentration and its MIC was determined to be 0.5 mM. Asterisks indicate significant differences (***p < 0.001, ****p < 0.0001) from TCA only by one-way ANOVA with Sidak’s correction for multiple comparisons. Source data

Finally, on the basis of the effects conventional BAs have on *C. difficile* pathogenic life cycle, we probed the impact of MCBAs on *C. difficile*. Although none of the tested MCBAs can induce spore germination (Extended Data Fig. 10a), several can inhibit germination including the prominent Tyr-CA, Phe-CA and Phe-βMCA (Fig. 5f and Extended Data Fig. 10b). Additionally, while *C. difficile* growth is enhanced in the presence of Tyr-CA and Phe-CA, it significantly lags with Tyr-βMCA and Phe-βMCA (Fig. 5g and Extended Data Fig. 10c). Thus, the sterol cores of MCBAs distinctly impact the growth of *C. difficile*. It is possible that CA conjugates are relatively poor at disrupting *C. difficile*’s membrane compared with the corresponding conjugates of βMCA, suggesting one potential mechanism for the inhibitory effects of MCBAs on *C. difficile* (Extended Data Fig. 2b). Furthermore, several MCBAs repress the P_*tcdA*_ promoter, indicating that *C. difficile* virulence may also be reduced by the MCBA pool (Fig. 5h and Extended Data Fig. 2c). Given that BSH treatment led to substantially lower *C. difficile* spore germination and growth in the small intestine along with elevated MCBAs (Figs. 4b,c,e and 5b,d), we propose that the MCBA pool may be playing a role in restricting *C. difficile* in vivo as we observed in vitro (Fig. 5f). Together, these results indicate that MCBAs have the capacity to alter *C. difficile* biology, but future work is needed to understand how MCBAs influence *C. difficile*.

## Discussion

There is a growing appreciation for the roles that BSH substrate preferences play in the management of intestinal disease^6,8,43^. Here we identified a key selectivity loop present in all Lactobacillaceae and 67% of BSH enzymes across diverse microbes (Fig. 2e) that predicts substrate specificity for glycine or taurine-conjugated BAs. Understanding the structural and molecular basis for selective deconjugation of glycine and taurine-conjugated BAs is crucial, as BSH deconjugation selectivity plays a role in various diseases. For example, deconjugation of taurine-conjugated BAs has been shown to impact the establishment of colonization resistance against *Klebsiella pneumoniae*, the development of colorectal cancer and more recently metabolic disease^43–45^. Our deeper focus on Lactobacillaceae BSH created a framework that we then used to define BSH substrate selection across the microbiota. These insights will help to inform future studies that aim to fine tune host-microbiota communication to promote health and prevent disease.

Since *C. difficile* is exquisitely sensitive to different BAs^27,28^, we employed sets of glycine and taurine-preferring BSHs to inhibit *C. difficile* (Figs. 3d, 4a and 5a). Our results suggest that leveraging microbial BA metabolism to limit *C. difficile* colonization may present an alternative strategy that avoids antibiotic use, the unspecified risks of FMTs and the variability inherent to live biotherapeutic products.

The extensive compilation of recently identified MCBAs expands the complexity of the BSH-BA pool and reinforces BSHs as the gatekeepers of bile acid metabolism, capable of reshaping the BA landscape in the gut^31,32^. BSH activity fuels the production of MCBAs (Figs. 4d and 5d). While the mechanism and purpose of this reconjugation is yet to be elucidated, our observation that MCBAs can inhibit *C. difficile* spore germination and impact growth and toxin expression introduces other mechanistic avenues by which BSH activity can be used to restore colonization resistance after antibiotic treatment (Fig. 5f,g). Additionally, these data suggest that BSHs are involved in MCBA production; indeed, other studies are already investigating the possibility of BSH-catalysed reconjugation^46,47^. Our demonstration that BSHs exhibit variable propensities for MCBA substrates supports a model in which BSHs directly shape the production and decomposition of the MCBA pool, and further bolsters their use as a precision tool to modulate BA metabolism in vivo (Fig. 4e and Extended Data Fig. 8).

While this work highlights the utility of BSHs as tools to affect intestinal health, their use as a therapeutic remain questionable. BSHs were neither shown to impact clinical signs of disease in vivo nor were they tested as a treatment for CDI (Supplementary Fig. 7), necessitating further studies to determine whether they have pre-clinical treatment potential. The importance of BSH selectivity is relevant to the human BA pool where glycine and taurine-conjugated BAs dominate. The mouse remains a useful model for studying BA dynamics, but its taurine bias can be limiting when investigating BSH substrate preferences. Furthermore, BSH activity is complicated by the emerging data indicating that these enzymes can catalyse reconjugation to generate MCBAs capable of activating host ligand-activated receptors^31,33,46,47^. Additionally, the range of physiologically relevant and effective MCBA concentrations are being defined and will be an area for future investigation. The respective rates of these deconjugation and reconjugation reactions, along with secondary BA transformations and amino acid metabolism, will all impact the landscape of BAs in the gut. This complexity perhaps explains why the BSH inhibition of *C. difficile* was not always congruent with the deconjugation in pre-FMT samples.

Tuning BSH activity may be most impactful when leveraged alongside other metabolic pathways. A continued understanding of BSH–substrate interactions will be required to support the use of BSHs as a targeted strategy to address intestinal disease. Moreover, fully restricting *C. difficile* may require sequestering key nutrients or amino acids in addition to producing inhibitory BAs^48^. Rationally engineering live biotherapeutic products equipped with several such functions may provide a path towards more efficiently and specifically treating CDI.

## Methods

### Phylogenetic analysis of BSH sequences in Lactobacillaceae

The method of BSH sequence analysis in lactobacilli was as previously described^37^.

Briefly, the whole-genome sequence data for lactobacilli were downloaded from NCBI in July 2021, resulting in a total of 3,712 nucleotide files. The data were subsequently built into a local BLAST Lactobacillaceae database. Updated BSH (15 sequences) and penicillin V acylase (PVA) (11 sequences) reference sets were manually created on the basis of previously characterized BSH and PVA proteins^37^. A BLASTx search was then performed using BLAST (v2.6.0), with the BSH reference set as the query and the Lactobacillaceae database as the BLAST database. Search results were filtered using custom code to identify sequences with >30% identity and at least 100 amino acids in length for downstream analysis.

The BSH and PVA reference sets were separately aligned using MUSCLE aligner (v3.8.2)^49^. A Stockholm multiple sequence alignment file generated using the alignment was then used to run the Hidden Markov Model using HMMER (v3.3.2)^50^ on the results of the BLASTx search to identify likely BSH proteins. Custom code was employed to remove results with *E* values <1 × 10^−99^ as false positives and to determine whether the amino acid sequence was likely a BSH or a PVA on the basis of its *E* value.

To facilitate the downstream phylogenetic analysis, CD-HIT (v4.8.1) clustering was used to cluster similar BSH sequences that shared at least 95% identity^51,52^. Custom code was employed to extract the consensus sequence from each cluster for the construction of a phylogenetic tree. CD-HIT cluster data were hand-curated to confirm the amino acid sequence of the representative BSH proteins from each of the 84 clusters and to remove redundant sequences. Geneious Primer (v2022.1) and CLC Genomics Workbench (v12) were used to visualize protein sequence alignments, construct phylogenetic trees and add metadata.

### Chemicals and materials

The following chemicals were used in this study: sodium taurocholate (Cayman Chemical), sodium glycocholate (Sigma Aldrich), sodium taurochenodeoxycholate (Sigma Aldrich), taurine (Sigma Aldrich), glycine (Sigma Aldrich), sodium acetate trihydrate (Thermo Fisher), sodium phosphate dibasic dihydrate (Sigma Aldrich), sodium phosphate monobasic dihydrate (Sigma Aldrich), dl-dithiothreitol (MP Biomedicals), trichloroacetic acid (Sigma Aldrich), ninhydrin (Sigma Aldrich), sodium citrate tribasic dihydrate (Sigma Aldrich), glycerol (Thermo Fisher) and crystal screens MCSG1-4 (Anatrace).

### Bacterial strains and growth conditions

BSH-expressing *Escherichia coli* Rosetta (DE3) pLysS were cultured at 37 °C, shaking overnight in LB broth supplemented with 30 μg ml^−1^ kanamycin and 20 μg ml^-1^ chloramphenicol (Cam) or in Terrific Broth (TB) supplemented with the same antibiotics for BSH overexpression. Lactobacillaceae species were cultured statically in MRS media at 37 °C in a Coy anerobic chamber (5% H_2_/10% CO_2_/85% N_2_) and *L. gasseri* ATCC 33323 *bsh* mutants were previously described^8^. *C. difficile* R20291 was statically cultured anaerobically using BHIS broth supplemented with 100 mg l^−1^ l-cysteine. BHIS agar supplemented with 0.1% TCA (TBHIS) was used to start cultures from spore stocks. *C. difficile* enumeration from intestinal contents or stool was performed on CCFA (cefoxitin, cycloserine and fructose agar) or TCCFA (containing 0.1% TCA). All agar plates were made using 1.5% agar. *C. difficile* growth curves were carried out in BHIS broth in clear flat-bottom plates containing 200 μl of media per well. Growths were performed in a Tecan plate reader within the anaerobic chamber at 37 °C for 24 h.

### Recombinant BSH cloning and protein expression

Selecting BSHs for characterization was informed by the Lactobacillaceae BSH phylogenetic tree in Fig. 1b. We intentionally sampled BSHs by selecting understudied clades or organisms to provide our survey with novelty and diversity. We also sampled from previously studied clades and organisms to provide comparison. Some BSHs were not amenable to heterologous expression and were excluded.

All BSH sequences cloned in this study were amplified from genomic DNA or were codon-optimized in synthesized genes (Integrated DNA Technologies (IDT)) using custom oligonucleotide primers (IDT) or were synthesized by BioBasic, as reported in Supplementary Tables 2 and 3. The BSH genes for LaciBSHa, LgasBSHa, LgasBSHb, LjohBSHa, LjohBSHb, LjohBSHc, LcriBSHa, LcriBSHb and LsalBSH were all amplified from genomic DNA. The genes for LmurBSH, LrogBSH, LplaBSH, LaviBSH, LgigBSH, LreuBSH, LcolBSH, LingBSH and the *E. faecalis* BSH were codon-optimized and synthesized. The genes for *Bifidobacterium longum*, *Bacteroides thetaiotaomicron, Faecalibacterium* sp., *Clostridiales* sp. and *Bacteroidales* sp. BSHs were codon-optimized, synthesized and subcloned into the pLic-His Cterm vector (ampicillin resistant) by BioBasic. PCR was performed using Phusion Flash High-Fidelity PCR master mix (Thermo Fisher) and products were purified using the QIAquick PCR Purification kit (Qiagen). Amplicons were cloned into the pETite C-His vector (Lucigen), purified using the Monarch Plasmid Miniprep kit (NEB) and sequenced. Overexpression *E. coli* cultures were grown at 37 °C with shaking in 1 l of TB with kanamycin and Cam until an OD_600_ ≈ 0.6 was reached. Isopropyl-β-d-thiogalactopyranoside (IPTG) was added to cultures to induce expression and cells were grown at 30 °C with shaking for 16–20 h. Cells were collected by centrifugation and stored at −80 °C.

### Site-directed mutagenesis

All BSH mutants were created with site-directed mutagenesis using Pfusion High-Fidelity DNA Polymerase (New England Biolabs). Primers were synthesized by IDT (Supplementary Table 4). Mutant plasmids were sequenced by Eton Biosciences to confirm the presence of the mutation. Mutant DNAs were transformed into *E. coli* BL21-G (DE3) cells and purified as described in the ‘Protein expression and purification for crystallography and mutant proteins’ section.

### Recombinant protein purification

To purify BSHs, frozen *E. coli* cell pellets were resuspended in 50 ml of lysis buffer (50 mM NaPO_4_, 300 mM NaCl, 20 mM imidazole, 10 mM 2-mercapoethanol, protease inhibitor (Roche), DNAse (Sigma), pH 8.0) and lysed by sonication. Cell debris were pelleted by centrifugation at 25,000 × *g* for 30 min at 4 °C. Lysates were run over a gravity column containing 4 ml of fresh HisPur cobalt resin (Thermo Fisher) that was equilibrated in wash buffer (50 mM NaPO_4_, 300 mM NaCl, 20 mM imidazole, pH 8.0). Bound BSHs were washed on the column at a rate of ~1 ml min^−1^ with 20 ml of wash buffer, eluted with 10 ml of elution buffer (wash buffer plus 150 mM imidazole and 10 mM dl-dithiothreitol) and flash frozen in liquid N_2_ to prevent oxidation. BSHs were quantified using the Qubit Protein Assay kit (Invitrogen) and protein purity was assessed using 10%, 14% or 4–20% SDS–PAGE gels (Thermo Fisher) (Supplementary Fig. 2).

### Protein expression and purification for crystallography and mutant proteins

Expression plasmids were transformed into BL21-G competent *E. coli* cells (New England Biolabs) and cultured under kanamycin (25 µg ml^−1^) and Cam (34 µg ml^−1^) or ampicillin (100 µg ml^−1^) selection. A single colony was selected the next day and grown overnight in 100 ml TB media (4.82 g TB EZMix powder, 400 µl glycerol, 100 ml deionized H_2_O) at 37 °C with shaking at 210 r.p.m. with kanamycin (25 µg ml^−1^) and Cam (34 µg ml^−1^) or ampicillin (100 µg ml^−1^). The next day, 50 ml of the overnight culture was added to 1.5 l TB media with the appropriate antibiotics and ~40 µl of Antifoam 204. The culture was then incubated at 37 °C with shaking at 210 r.p.m. for expression. Expression was induced when optical density (OD)_600_ reached 0.6–0.8 with 100 µM IPTG, and the culture was incubated overnight at 18 °C.

*Clostridiales* sp. BSH was expressed using autoinduction instead of IPTG induction^53^. A single colony or 20 µl of glycerol stock was added to 10 ml MDAG-135 media (9.4 ml double distilled (ddH_2_) O; 20 µl 1 M MgSO_4_; 2 µl 1,000x trace metals (50 mM ferric chloride, 20 mM calcium chloride,10 mM manganese chloride, 10 mM zinc sulfate, 2 mM cobalt chloride, 2 mM cupric chloride, 2 mM nickel chloride, 2 mM sodium molybdate, 2 mM sodium selenite, 2 mM boric acid); 70 µl 50% (w/v) glucose; 40 µl 25% (w/v) aspartate; 200 µl 50x M (1.25 M sodium phosphate dibasic, 1.25 M sodium phosphate monobasic, 2.5 M ammonium chloride, 0.25 M sodium sulfate); 200 µl 17 amino acids (20 essential except cysteine, tyrosine and methionine at 200 µg ml^−1^ each); 80 µl 25 mg ml^−1^ methionine; and 100 µg ml^−1^ ampicillin). The culture was grown overnight at 37 °C with shaking at 210 r.p.m. The next day, the overnight culture was added to 1 l ZYM-5052 media (10 g N-Z-amine AS; 5 g yeast extract; 958 ml ddH_2_O; 2 ml 1 M MgSO_4_; 0.2 ml 1,000x trace metals mix; 20 ml 50×5052 (0.5% glycerol, 0.05% glucose, 0.2% alpha-d-lactose, 730 ml ddH_2_O); and 20 ml 50x M) with ampicillin and ~40 µl of Antifoam 204. The culture was then incubated at 37 °C with shaking at 210 r.p.m. for expression. Expression was autoinduced via incubation at 37 °C; when the OD_600_ reached 1–2, the culture was then incubated for approximately 24 h at 18 °C until optical density reached 13–15.

The cells were then pelleted in a Sorvall Instruments RC-3B centrifuge at 4,500 × *g* for 20 min at 4 °C. The cell pellet was resuspended in lysis buffer (30 ml BSH buffer A: 50 mM sodium phosphate, 300 mM sodium chloride, 10 mM imidazole, pH 8.0 with one cOmplete Mini EDTA-free protease inhibitor cocktail tablet (Roche), lysozyme, 50 µg ml^−1^ DNAse and 10 mM 2-mercaptoethanol). Cells were then lysed by sonication on ice using a Thermo Fisher Sonic dismembrator model 500 with 0.5 s pulses for 1.5 min three times at 40%, 50% and 60% intensity. The cell suspension was pelleted by centrifugation in a Beckman Coulter J2-HC centrifuge at 17,000 × *g* for 50 min at 4 °C. The supernatant was syringe-filtered using a 0.22 µm filter.

The filtrate was flowed over a HisTrap HP 5 ml column (Cytiva) using the Aktaexpress FPLC (Amersham Bioscience) and washed with BSH buffer A. The bound protein was eluted in a stepwise fashion with 100% BSH buffer B (50 mM sodium phosphate, 300 mM sodium chloride, 300 mM imidazole, pH 8.0). Fractions containing the protein of interest were combined and flowed over a HiLoad 16/60 Superdex 200 gel filtration column (GE Life Sciences). Samples were eluted in S200 sizing buffer (20 mM HEPES, 50 mM NaCl, pH 7.4). Fractions containing the protein of interest were analysed for purity using SDS–PAGE, and those with >95% purity were combined and concentrated using 30 kDa cut-off molecular weight centrifuge concentrators (EMD Millipore) to ~10 mg ml^−1^. The final protein concentration was determined using an ND-1000 spectrophotometer. Dithiothreitol was added to the concentrated fractions at a final concentration of 10 mM, and samples were snap-frozen in liquid nitrogen and stored at −80 °C.

### BSH-specific activity assays

Activity assays were performed as previously described for the BSHs surveyed in Fig. 1c and Supplementary Fig. 14 (ref. ^8^). Briefly, the assay reacted 10–25 nM BSH with 9 mM conventional BAs for 5 min, or 100 nM BSH with 4.5 mM MCBAs/penV, for 1 h in 50 μl volumes. Reactions were carried out in 0.1 mM sodium phosphate, 10 mM dithiothreitol (pH 6.0) and stopped with 50 μl of trichloroacetic acid. To determine the quantity of amino acid or 6-APA released, the colorimetric ninhydrin reaction was carried out. Of the quenched BSH reaction, 25 μl was added to 475 μl of ninhydrin buffer (0.3 ml glycerol, 0.175 ml 0.5 M sodium citrate (pH 5.5), 0.25% ninhydrin reagent) and boiled for 14 min. A standard curve of the respective conjugated amino acid or 6-APA was prepared for each assay. Absorbance was measured at 570 nm in clear flat-bottom plates in a Tecan Infinite F200 Pro plate reader. Specific activity is reported as μmol amino acid released per second per μmol BSH.

### pH screens

The optimal pH of each BSH enzyme tested in Fig. 2f was determined using the preferred substrate, either taurocholic acid sodium salt (TCA) or sodium glycocholate hydrate (GCA). The substrate preference was determined either from Fig. 1 (all *Lactobacillus* BSHs) or by performing a single pH screen (one replicate) as described below using both TCA and GCA for all 5 pH values tested. The substrate with faster turnover was then used to complete the full pH screen in triplicate.

To determine the optimal pH, the activity of each BSH was tested with the preferred substrate at pH 5.0, 5.5, 6.0, 6.5 and 7.0. pH screen reactions were prepared to a final volume of 50 µl consisting of 35 µl buffer (50 mM sodium acetate (for pH 5.0 and 5.5) or 50 mM sodium phosphate (for pH 6.0, 6.5 and 7.0)), each with 10 mM dithiothreitol, 5 µl preferred substrate (either TCA or GCA, final reaction concentration 10 mM) and 10 µl enzyme (final concentration ranging from 10 nM to 1,000 nM). Substrate and buffer were plated and allowed to incubate at 37 °C for 5 min before 10 μl enzyme was added to initiate the reaction. Reactions were carried out at 37 °C and quenched with 50 µl of 15% (w/v) trichloroacetic acid at five time points ranging from 2 min to 3 h based on enzyme activity.

The amount of liberated amino acid present was quantitated using ninhydrin. First, reactions were centrifuged at 4,000 × *g* for 2 min. Quenched reaction (10 µl) was added to 190 µl ninhydrin reaction (62.5 ml of 1% ninhydrin in 0.5 M sodium citrate (pH 5.5), 150 ml glycerol, 25 ml 0.5 M sodium citrate (pH 5.5)) in a 96-well PCR plate. A standard curve of glycine or taurine was also created for analysis using the ninhydrin reaction. Using a thermocycler, the mixtures were heated to 90 °C for 14 min and then cooled at 25 °C for 15 min. Ninhydrin mixture (150 µl) was then added to a 96-well flat-black plate (Grenier 655906) and the absorbance was measured at 570 nm using a Clariostar Plus plate reader (BMG Labtech). The standard curve was used to quantitate the amount of free amino acid liberated by the reaction. Reaction curves over time were fit using linear regression and the reaction rate was divided by protein concentration to yield specific activity. Specific activities are the mean ± s.d of three biological replicates. Optimal pH was determined by selecting the pH at which turnover of the substrate was the fastest (Supplementary Fig. 9).

### Extant and mutant BSH-specific activities

Specific-activity assays in Fig. 2i,j and the BSHs presented were performed by adding 10 µl enzyme (10–500 nM final concentration, depending on enzyme) to 5 µl 100 mM taurocholic acid or glycocholic acid (10 mM or 2 mM final concentration glycolithocholic acid sodium salt (GLCA) and sodium taurolithocholate (TLCA)) and 35 µl assay buffer (optimal pH buffer, as determined in pH screen section) for a total volume of 50 µl. GLCA and TLCA stocks were prepared by making a 20 mM stock of substrate in 20% ethanol for GLCA and 20% DMSO for TLCA for a final reaction concentration of 2 mM substrate due to poor solubility. Control reactions contained either no enzyme or no substrate. Reactions were incubated at 37 °C and quenched at five time points with 50 µl 15% (w/v) trichloroacetic acid. Reaction progress was quantitated using the ninhydrin reaction (as described in pH screen section). Reaction curves over time were fitted using linear regression and the reaction rate was divided by protein concentration to yield specific activity. Specific activities are the mean ± s.d. of three biological replicates.

### Protein crystallography

Crystals were generated at 20 °C using sitting drop vapour diffusion for all specimens obtained except those of LgasBSHa grown with taurine and CDCA, which employed hanging drop vapour diffusion. Sitting drop trays were set up using the Douglas Instruments Oryx4 instrument and Hampton Research 3-well midi crystallization plates (Swissci). Hanging drops were manually plated using EasyXtal 15-well tool trays (Qiagen). Specific crystallization conditions follow. LaciBSHa: crystallant composed of 0.15 M dl-malic acid, pH 7.0, with 20% (w/v) PEG 3350. Crystals formed in a 2:1 protein (11.57 mg ml^−1^) to crystallant ratio. LgasBSHa with taurine: crystallant composed of 0.2 M potassium sulfate, 20% (w/v) PEG 3350. Crystals formed in a 1:2 protein (9.5 mg ml^−1^) to crystallant ratio. LgasBSHa with taurine and CDCA: a condition containing 18% (w/v) PEG 3350, 0.2 M ammonium chloride, pH 6.3, in a hanging drop tray was streak seeded with crystals grown using the sitting drop method in 20% (w/v) PEG 3350, 0.2 M ammonium chloride (pH 6.3). Crystals were grown in a 1:2 protein (9.55 mg ml^−1^) to crystallant ratio. The resultant crystals were soaked in 1:1 ratio of crystallant:100 mM taurochenodeoxycholic acid for 24 h before looping. LgasBSHb: crystallant composed of 0.2 M magnesium chloride, 0.1 M sodium cacodyolate:HCl, pH 6.5, 20% (w/v) PEG 1000. Crystals formed in a 1:2 protein (10.75 mg ml^−1^) to crystallant ratio. LjohBSHc: crystallant composed of 0.1 M sodium citrate:HCl, pH 5.6, 10% (w/v) PEG 4000, 10% (v/v) isopropanol. Crystals formed in a 2:1 protein (10.34 mg ml^−1^) to crystallant ratio. LingBSH: crystallant composed of 0.2 M calcium acetate hydrate, 0.1 M Tris:HCl, pH 7.0, 20% (w/v) PEG 3000. Crystals formed in a 1:2 protein (13.8 mg ml^−1^) to crystallant ratio. LreuBSH: crystallant composed of 0.1 M Bis-Tris Propane:HCl, pH 7.0, 1.5 M ammonium sulfate. Crystals formed in a 2:1 protein (13.3 mg ml^−1^) to crystallant ratio.

Crystals were cryo-protected in the conditions described above with 20% glycerol before looping and flash cooling in liquid nitrogen. Diffraction data were collected at 100 K at either APS 23ID-D (LgasBSHa with taurine and CDCA, LingBSH), APS 23ID-B (LaciBSHa, LgasBSHa with taurine, LgasBSHb, LjohBSHc) or ALS 5.0.2 (LreuBSH). Data were processed and scaled with XDS (v2.3.2)^54^ and Phenix (v1.17.1-3660) was employed to perform molecular replacement with the model PDB 2HEZ. Output models from Phaser were improved with Autobuild^55^ and structures were refined using phenix.refine^55^ with iterative cycles of manual adjustment using Coot (v0.9.4.1)^56^. Final coordinates were deposited into the RCSB Protein Data Bank under the codes 7SVE, 7SVF, 7SVG, 7SVH, 7SVI, 7SVJ and 7SVK.

### Glycine modelling

Glycine was modelled into the active site of the unliganded structure of LgasBSHb by superimposing this enzyme’s active site with that of the structure of LgasBSHa in complex with both taurine and CDCA. Glycine was then manually positioned over the backbone of taurine using PyMOL (v2.3.2) to approximate the binding mode of glycine.

### Circular dichroism

Secondary structures of the wild-type and mutant proteins were evaluated using circular dichroism^57^. Enzyme (0.125 mg ml^−1^) in CD buffer (10 mM potassium phosphate pH 7.4, 100 mM potassium fluoride) was placed into a 1 mm cuvette. Scan spectra from 185 to 260 nm were acquired at 20 °C using the Chirascan Plus instrument (Applied Photolysis). A background spectrum of buffer alone was acquired and subtracted out to correct for background signal. Spectra were smoothed before plotting. Calculation of protein secondary structure was performed using the DichroWeb server (http://dichroweb.cryst.bbk.ac.uk/html/process.shtml)^58^. Data were assessed using the CONTIN-LL method^59^, with reference set SMP180 (Supplementary Table 5)^60^. Output classifications of secondary structure are as previously described^61^.

### Identification and characterization of BSH sequences

The Integrated Gene Catalog contains metagenomic sequencing data from 249 human samples examined in the Metagenomics of the Human Intestinal Tract (MetaHit) project along with 1,018 collections of extant human gut metagenomics data to form an overall database with 9,878,647 unique translated bacterial protein sequences^41^. BSH enzymes were identified from the IGC using a structural metagenomics pipeline as reported previously^62^. Briefly, each sequence was aligned pairwise to 5 representative BSH enzymes with reported crystal structures (PDB: 2BJF, 2HF0, 4WL3, 6UFY and 5HKE) using Protein-Protein BLAST (BLASTP v2.5.0+)^63–67^. Candidate sequences with ≥25% identity to any representative BSH enzyme were then assessed for the presence of 5 conserved residues: C2, R18, D21, N175 and R228 (relative to PDB: 2BJF). Sequences that both met the identity threshold and contained all 5 conserved residues were accepted as BSH enzymes (Supplementary Table 4). Accepted sequences were aligned in a multiple sequence alignment and BSH structural class was assigned accordingly. Taxonomy was assigned to representative GUS sequences using BLASTP (v2.5.0+) as reported previously^63,68^. Accepted sequences were processed with the Enzyme Function Initiative–Enzyme Similarity Tool (https://efi.igb.illinois.edu/efi-est/) to create a sequence similarity network, represented using an *E* value of 1 × 10^−100^ (ref. ^69^). Each node represents sequences bearing ≥95% identity.

### Spore preparation

*C. difficile* spores were prepared as previously described^28,70^. Briefly, *C. difficile* was grown at 37 °C anaerobically for 1 week in Clospore media^71^. Spores were collected by centrifugation, washed with water, heat treated for 20 min at 65 °C and stored at 4 °C. Spores were plated on BHIS and TBHIS agar to make sure no viable cells were present.

### Spore germination and outgrowth assays

Spore germination experiments were modified from refs. ^70,72^. Conventional BAs were dissolved in water and MCBAs were dissolved in methanol. For the inhibition of germination experiments, the germinant TCA was used across conditions at 2 mM. CDCA was used at 0.75 mM as a positive control for germination inhibition and all other BAs were tested at the same concentration (0.75 mM). Germination reactions were carried out anaerobically in 100 μl volumes in PBS for 30 min. Reactions were subsequently serially diluted and plated on TBHIS and BHIS agar. Percent germination for each BA was calculated as (100 × (c.f.u.s on BHIS / c.f.u.s on TBHIS)) and was normalized to germination with TCA alone. For the germination reactions with MCBAs, all BAs were used at 1.9 mM and percent germination was calculated and normalized as stated above.

### MICs

BA tolerance measured by MIC was adapted from the bile tolerance assay^73^. Overnight *C. difficile* cultures (~10^8^ c.f.u.s ml^−1^) were inoculated at 1% into BHIS containing a range of BA concentrations. Cultures were anaerobically incubated for 24 h at 37 °C. Following incubation, cultures were serially diluted in PBS and plated on BHIS agar to determine whether the concentration of BA tested inhibited growth relative to the starting inoculum.

### Propidium iodide (PI) staining

Exponentially growing *C. difficile* cultures were captured and washed 3 times with PBS before being back diluted to a final OD_600_ = 0.1 into PBS containing BAs at 0.25× the MIC for 30 min. Bacteria were exposed to 150 μM SDS as positive controls for detergent-induced membrane damage and PI staining. Following ΒΑ exposure, bacteria were stained for 30 min at 37 °C with 20 μg ml^−1^ PI using slight modifications to a previous method^73,74^. Stained bacteria were diluted 1:10 in PBS and PI fluorescence was measured from flat-bottom clear plates (excitation: 540 nm; emission: 610 nm) in 100 μl volumes in a Tecan Infinite F200 Pro plate reader and background fluorescence was subtracted.

### P_*tcdA*_-mCherry reporter assay

*C. difficile* R20291 containing either pDSW1728-P*tcdA*-*mCherry* or the empty vector pDSW1728 was grown anaerobically on BHIS agar + 3 μg ml^−1^ thiamphenicol^75^. A single colony was inoculated into a 5 ml culture of BHIS broth + 3 μg ml^−1^ thiamphenicol and grown overnight at 37 °C. The overnight culture was diluted 1:100 into a fresh 3 ml culture of BHI broth + 3 μg ml^−1^ thiamphenicol with individual BAs and grown at 37 °C for 24 h. Culture (500 μl) was mixed with 120 μl of a 5X fixation cocktail: 100 μl 16% paraformaldehyde and 20 μl 1 M NaPO_4_ buffer (pH 7.4). The sample was incubated anaerobically at room temperature for 30 min, then incubated in the dark for 30 min on ice outside of the anaerobic chamber. Fixed cells were washed 3 times with PBS, resuspended in 30 μl of PBS and left in the dark at 4 °C overnight to allow for chromophore maturation. mCherry fluorescence was measured at excitation/emission wavelengths of 550/610 nm using a Tecan Infinite F200 Pro plate reader. Absorbance at 600 nm was measured on the same plate reader. Data are displayed as the ratio of fluorescence to absorbance. Baseline fluorescence from the empty vector was subtracted from all measurements.

### Pre-FMT sample collection

All consenting patients undergoing FMT for rCDI at the University of North Carolina from January to December 2017 in a prospective registry were enrolled for faecal collection. rCDI was defined as a patient having at least the third episode of CDI. There were no exclusion criteria for participation in the registry specifically, although participants were by definition undergoing FMT under the care of a physician who judged the benefits to outweigh the risks. Patient stool samples from 2 weeks before FMT (pre-FMT) were collected. The study was approved by the UNC Insitutional Review Board (no. 16-2283). Informed written consent was obtained from recipients. Stool samples were collected and de-identified by the research team.

### Animals and housing

Male and female C57BL/6J mice (5 weeks old) were purchased from Jackson Labs (Bar Harbor) for use in infection experiments. The food, bedding and water were autoclaved, and all cage changes were performed in a laminar flow hood. The mice were subjected to a 12 h light and 12 h dark cycle, and were housed at an average temperature of 70 °F and 35% humidity. Animal experiments were conducted in the Laboratory Animal Facilities located on the NCSU CVM campus. The animal facilities are managed by full-time animal care staff coordinated by the Laboratory Animal Resources (LAR) division at NCSU. The NCSU CVM is accredited by the Association for the Assessment and Accreditation of Laboratory Animal Care International (AAALAC). Trained animal handlers in the facility fed and assessed the status of animals several times per day. Those assessed as moribund were humanely euthanized by CO_2_ asphyxiation. This protocol is approved by NC State’s Institutional Animal Care and Use Committee (IACUC).

### Mouse sample collection

Male C57BL/6J mice (6–8 weeks old, *n* = 16 total) were given 0.5 mg ml^−1^ cefoperazone in their drinking water for 5 d to make them susceptible to CDI^76^. Subsequently, the mice were then given normal water (Gibco) for 2 d, after which groups of mice (*n* = 3–4) were killed at day 0. Small intestinal and caecal contents from each mouse were collected and samples were flash frozen in liquid N_2_.

### Ex vivo growth and spore germination assay

To measure BSH-dependent inhibition of *C. difficile* ex vivo in pre-FMT stool and mouse intestinal contents, frozen material was thawed and diluted 1:3 in PBS. For growth experiments, pre-FMT stool samples were treated with PBS, the taurine-preferring BSH cocktail T-BSH (LgasBSHa, LjohBSHc and LsalBSH), the glycine-preferring BSH cocktail G-BSH (LjohBSHa, LgasBSHb and LingBSH) or the combined broad acting G&T-BSH cocktail for 30 min at 37 °C. Mouse contents were treated with PBS or the G&T-BSH cocktail. All BSHs had a final concentration of 0.1 μM each. Immediately afterwards, exponentially growing *C. difficile* was inoculated into samples to a final concentration of 10^5^ c.f.u.s ml^−1^. *C. difficile* was cultured anaerobically at 37 °C and enumerated on TCCFA at 8 and 24 h.

To measure BSH-dependent inhibition of germination in mouse small intestinal contents, 1:3 diluted samples were treated with PBS or the G&T-BSH cocktail for 8 h at 37 °C. *C. difficile* spores (10^6^ ml^−1^) were then inoculated in the small intestinal contents for 30 min and samples were promptly diluted and enumerated on CCFCA and TCCFA. Percent germination was calculated as (100% × (c.f.u.s on CCFA / c.f.u.s on TCCFA)).

### Mouse model of CDI

Male C57BL/6J mice (6–8 weeks old) housed at 3 mice per cage were given 0.5 mg ml^−1^ cefoperazone in their drinking water for 5 d to make them susceptible to CDI^76^. Subsequently, the mice were given normal water (Gibco) for 2 d. Mice were orally gavaged with the T-BSH cocktail containing 10 μg each of LgasBSHa, LjohBSHc and LsalBSH prepared by exchanging the BSHs from their storage buffer into PBS. PBS or the BSH cocktail was administered once on day −1, twice on days 0 and 1, and once on day 2 before necropsy. Infected mice were challenged with 10^5^ *C. difficile* spores at day 0, weighed daily and monitored for clinical signs of distress (ruffled fur, hunched posture, slow ambulation). At day 2, all mice (PBS *n* = 6, BSH *n* = 9, Cd + PBS *n* = 10, Cd + BSH *n* = 13) were humanely killed and necropsy was performed. Small intestinal and caecal contents were collected for enumeration of vegetative *C. difficile* c.f.u.s using TCCFA. Contents were also immediately frozen and stored at −80 °C for later metabolomic analysis.

### MCBA synthesis

All MCBAs were purified according to literature precedent and characterization data are consistent with reported data^31,33,42,77,78^. Synthesis of the conjugated bile acids was adapted from a previously published method^79^. Bile acid (0.25 mmol, 1 equiv) was dissolved in anhydrous tetrahydrofuran (4.9 ml, 0.05 M) and cooled to 0 °C with stirring. Ethyl chloroformate (0.3 mmol, 1.2 equiv) was added, followed by triethylamine (0.3 mmol, 1.2 equiv) and the reaction was stirred for 1.5 h at 0 °C. After conversion of starting material by thin-layer chromatography (TLC), a cold solution of amino acid (0.375 mmol, 1.5 equiv) and either NaHCO_3_ or NaOH (0.375 equiv, 1.5 equiv) in H_2_O (4.9 ml, 0.05 M) was added in one portion. Then, the reaction was stirred for 2 h, allowing it to gradually warm to r.t. After this time, tetrahydrofuran was removed in vacuo, then 2 M HCl was added to acidify the reaction mixture to pH < 2, producing a white precipitate. The mixture was extracted in ethyl acetate (3 × 20 ml), then the combined organic layers were washed with brine (50 ml), dried over Na_2_SO_4_ and concentrated in vacuo. The crude residue was purified over silica gel using methanol and dichloromethane with 1% acetic acid.

Organic solutions were concentrated under reduced pressure on a Büchi rotary evaporator using a water bath. Chromatographic purification of products was accomplished by flash chromatography on Silicycle F60 silica gel. All reactions were carried out in well-ventilated fume hoods. TLC was performed on Silicycle 250 μm silica gel plates. Visualization of the developed chromatogram was performed by irradiation with 254 nm UV light or treatment with a solution of ceric ammonium molybdate stain, followed by heating. Yields refer to purified compounds unless otherwise noted.

^1^H and ^13^C NMR spectra are displayed in Supplementary Fig. 10. Spectra were recorded on a Bruker 600 (600 and 151 MHz for ^1^H and ^13^C, respectively) instrument using Bruker TopSpin (v3.6.0) and are internally referenced to residual protiosolvent signals of CD_3_OD at *δ* 3.31 and 49.00 and (CD_3_)_2_SO at 2.50 and 39.52. Data for ^1^H NMR were analysed using MNova (v14.2.0) and are reported as follows: chemical shift (*δ* ppm), integration, multiplicity (s = singlet, br s = broad singlet, d = doublet, t = triplet, q = quartet, m = multiplet) and coupling constant (Hz). Data for ^13^C NMR are reported in terms of chemical shift and no special nomenclature is used for equivalent carbons.

Glu-CDCA, Ile-CA, Leu-CA, Phe-CA, Trp-CDCA and Tyr-CA were purified according to literature precedent and characterization data are consistent with reported data^31,33^.

Ala-CA: NaHCO_3_ was used as the inorganic base. Product was purified using 3–10% CH_3_OH in CH_2_Cl_2_ with 1% acetic acid to obtain a 92% yield as a white amorphous solid. ^1^H NMR (600 MHz, CD_3_OD) *δ* 4.40–4.29 (m, 1H), 3.96 (t, *J* = 3.1 Hz, 1H), 3.80 (q, *J* = 3.1 Hz, 1H), 3.41–3.36 (m, 1H), 2.33–2.21 (m, 3H), 2.20–2.12 (m, 1H), 2.00–1.93 (m, 2H), 1.92–1.77 (m, 4H), 1.77–1.70 (m, 1H), 1.69–1.63 (m, 1H), 1.63–1.50 (m, 5H), 1.47–1.40 (m, 2H), 1.38 (d, *J* = 6.9 Hz, 3H), 1.36–1.24 (m, 3H), 1.11 (qd, *J* = 11.7, 5.5 Hz, 1H), 1.03 (d, *J* = 6.6 Hz, 3H), 0.98 (td, *J* = 14.2, 3.3 Hz, 1H), 0.91 (s, 3H), 0.71 (s, 3H). ^13^C NMR (151 MHz, MeOD) *δ* 176.46, 74.01, 72.83, 69.02, 49.85, 48.04, 47.46, 43.14, 42.93, 40.97, 40.40, 36.83, 36.47, 35.86, 35.82, 33.85, 33.09, 31.13, 29.52, 28.65, 27.81, 24.21, 23.18, 17.80, 17.76, 13.01. HRMS (ESI) exact mass calculated for (M + H)^+^ (C_27_H_46_NO_6_) requires *m*/*z* 480.3320, found 480.3320 with a difference of 0.00 ppm.

His-CDCA: NaOH was used as the inorganic base. Product was purified using 3–10% CH_3_OH in CH_2_Cl_2_ with 1% acetic acid to obtain a 41% yield as a white amorphous solid. ^1^H NMR (600 MHz, CD_3_OD) *δ* 8.52 (s, 1H), 8.24 (s, 1H, NH), 7.20 (s, 1H), 4.58 (dd, *J* = 8.0, 5.1 Hz, 1H), 3.79 (q, *J* = 3.0 Hz, 1H), 3.38 (tt, *J* = 11.7, 4.6 Hz, 1H), 3.24 (dd, *J* = 15.1, 5.2 Hz, 1H), 3.05 (dd, *J* = 15.1, 8.0 Hz, 1H), 2.34–2.21 (m, 2H), 2.20–2.08 (m, 1H), 2.03–1.93 (m, 2H), 1.93–1.81 (m, 3H), 1.78–1.69 (m, 2H), 1.69–1.57 (m, 2H), 1.57–1.40 (m, 5H), 1.40–1.22 (m, 5H), 1.21–1.06 (m, 3H), 1.02–0.96 (m, 1H), 0.95 (d, *J* = 6.5 Hz, 3H), 0.93 (s, 3H), 0.68 (s, 3H). ^13^C NMR (151 MHz, CD_3_OD) *δ* 176.35, 175.72, 132.60, 118.14, 72.84, 69.06, 57.31, 54.33, 51.56, 43.67, 43.15, 41.06, 40.74, 40.46, 36.96, 36.54, 36.21, 35.91, 34.06, 34.00, 33.10, 31.35, 29.27, 24.63, 23.39, 21.78, 18.92, 12.18. HRMS (ESI) exact mass calculated for (M + H)^+^ (C_30_H_48_N_3_O_5_) requires *m*/*z* 530.3589, found 530.3591 with a difference of 0.38 ppm.

Ser-CA: NaHCO_3_ was used as the inorganic base. Product was purified using 6–12% CH_3_OH in CH_2_Cl_2_ with 1% acetic acid to obtain a 74% yield as an off-white amorphous solid. ^1^H NMR (600 MHz, CD_3_OD) *δ* 4.33 (s, 1H), 3.95 (t, *J* = 3.1 Hz, 1H), 3.88–3.75 (m, 3H), 3.41–3.36 (m, 1H), 2.40–2.33 (m, 1H), 2.31–2.16 (m, 3H), 2.03–1.98 (m, 1H), 1.95–1.71 (m, 7H), 1.69–1.63 (m, 1H), 1.63–1.51 (m, 5H), 1.47–1.35 (m, 4H), 1.34–1.26 (m, 1H), 1.11 (qd, *J* = 11.7, 5.5 Hz, 1H), 1.04 (d, *J* = 6.4 Hz, 3H), 0.98 (td, *J* = 14.1, 3.3 Hz, 1H), 0.91 (s, 3H), 0.71 (s, 3H). ^13^C NMR (151 MHz, CD_3_OD) *δ* 176.47, 74.03, 72.88, 69.04, 63.77, 49.85, 48.04, 47.50, 43.20, 42.99, 41.02, 40.46, 37.00, 36.49, 35.90, 35.86, 34.17, 33.04, 31.18, 29.58, 28.72, 27.88, 24.24, 23.17, 17.80, 13.01. HRMS (ESI) exact mass calculated for (M + H)^+^ (C_27_H_46_NO_7_) requires *m*/*z* 496.3269, found 496.3267 with a difference of 0.40 ppm.

Trp-CA: NaOH was used as the inorganic base. Product was purified using 6–12% CH_3_OH in CH_2_Cl_2_ with 1% acetic acid to obtain 49% yield as an off-white amorphous solid. ^1^H NMR (600 MHz, CD_3_OD) *δ* 7.58 (d, *J* = 7.9 Hz, 1H), 7.34 (d, *J* = 8.1 Hz, 1H), 7.14–7.07 (m, 2H), 7.02 (t, *J* = 7.4 Hz, 1H), 4.75 (dd, *J* = 8.4, 4.9 Hz, 1H), 3.93 (t, *J* = 3.1 Hz, 1H), 3.81 (q, *J* = 3.1 Hz, 1H), 3.42–3.35 (m, 2H), 3.17 (dd, *J* = 14.7, 8.4 Hz, 1H), 2.34–2.20 (m, 3H), 2.14–2.06 (m, 1H), 2.03–1.93 (m, 2H), 1.86–1.76 (m, 3H), 1.76–1.65 (m, 3H), 1.64–1.50 (m, 6H), 1.49–1.41 (m, 1H), 1.41–1.29 (m, 2H), 1.28–1.15 (m, 2H), 1.13–1.05 (m, 1H), 1.05–1.00 (m, 1H), 0.98 (d, *J* = 6.7 Hz, 3H), 0.92 (s, 3H), 0.67 (s, 3H). ^13^C NMR (151 MHz, CD_3_OD) *δ* 176.70, 175.49, 137.97, 128.83, 124.30, 122.35, 119.78, 119.21, 112.27, 111.07, 74.04, 72.84, 69.08, 54.62, 47.94, 47.40, 43.10, 42.90, 40.91, 40.40, 36.71, 36.43, 35.84, 35.80, 33.79, 32.96, 31.12, 29.47, 28.54, 28.45, 27.80, 24.19, 23.14, 17.70, 12.96. HRMS (ESI) exact mass calculated for (M + H)^+^ (C_35_H_51_N_2_O_6_) requires *m*/*z* 595.3742, found 595.3737 with a difference of 0.84 ppm.

Phe-βMCA: product isolated in 91% yield as a white solid. ^1^H NMR (600 MHz, CD_3_OD) *δ* 7.30 – 7.16 (m, 5H), 4.64 (t, *J* = 7.0 Hz, 1H), 3.58 (t, *J* = 2.8 Hz, 1H), 3.48 (ddt, *J* = 24.6, 10.8, 3.9 Hz, 2H), 3.22 (dd, *J* = 14.0, 4.6 Hz, 1H), 2.94 (dd, *J* = 13.9, 9.1 Hz, 1H), 2.19 (ddt, *J* = 19.2, 13.9, 6.9 Hz, 1H), 2.11–2.00 (m, 2H), 1.94 (dtt, *J* = 10.1, 7.3, 2.8 Hz, 1H), 1.81 (tdd, *J* = 17.1, 9.6, 4.8 Hz, 1H), 1.77–1.66 (m, 4H), 1.66–1.57 (m, 2H), 1.50–1.32 (m, 7H), 1.27–1.12 (m, 5H), 1.10–1.00 (m, 5H), 0.93 (d, *J* = 6.6 Hz, 3H), 0.69 (s, 3H). ^13^C NMR (151 MHz, MeOD) *δ* 176.63, 138.67, 130.26, 129.37, 127.70, 77.16, 74.34, 71.83, 57.19, 56.46, 49.21, 44.75, 41.38, 41.13, 39.50, 38.44, 36.76, 36.70, 36.48, 34.92, 33.85, 33.21, 30.67, 29.58, 28.19, 26.16, 21.98, 19.01, 12.63. HRMS (ESI) exact mass calculated for (M + H)^+^ (C_33_H_50_NO_6_) requires *m*/*z* 556.3633, found 556.3636 with a difference of 0.54 ppm.

Tyr-βMCA: product isolated in 56% yield as a white solid. ^1^H NMR (600 MHz, MeOD) *δ* 7.03 (d, *J* = 7.9 Hz, 2H), 6.68 (d, *J* = 7.8 Hz, 2H), 4.53 (s, 1H), 3.58 (t, *J* = 2.8 Hz, 1H), 3.53–3.42 (m, 2H), 3.12 (d, *J* = 13.5 Hz, 1H), 2.88–2.80 (m, 1H), 2.25–2.17 (m, 1H), 2.10–2.00 (m, 2H), 1.97–1.90 (m, 1H), 1.88–1.79 (m, 1H), 1.78–1.67 (m, 4H), 1.66–1.57 (m, 2H), 1.51–1.34 (m, 7H), 1.27–1.14 (m, 5H), 1.09 (s, 3H), 1.08–1.01 (m, 2H), 0.93 (dd, *J* = 228.5, 6.2 Hz, 3H), 0.70 (s, 3H). ^13^C NMR (151 MHz, CD_3_OD) *δ* 157.06, 131.33, 129.71, 116.05, 77.18, 74.36, 71.84, 57.20, 56.48, 49.23, 44.77, 41.39, 41.15, 39.51, 37.97, 36.77, 36.76, 36.48, 34.93, 34.07, 33.29, 30.68, 29.60, 28.21, 26.15, 21.99, 19.02, 12.63. HRMS (ESI) exact mass calculated for (M + H)^+^ (C_33_H_50_NO_7_) requires *m*/*z* 572.3582, found 572.3585 with a difference of 0.52 ppm.

### Microbial conjugated AA-BA pooled activity assays

BSH activity assay with the pooled AA-BAs was carried out using similar reaction conditions as described above in the BSH activity assays with purified BAs. BSH (100 nM) was reacted with (2.5 mg ml^−1^) AA-CA, AA-CDCA, AA-DCA or AA-βMCA for 1 h in 50 μl volumes in triplicate. Samples (10 μl) were taken at 5 min, 30 min and 1 h, immediately quenched by diluting them into 90 μl methanol and stored at −80 °C. Reactions containing no BSH were used as a negative control. Percent deconjugation was calculated at each time point as follows:

1 $$\begin{array}{l}{\%}\;{\mathrm{Deconjugation}} = 100{\%}\\ \times \frac{{({\mathrm{BA}}\;{\mathrm{peak}}\;{\mathrm{area}}\;{\mathrm{at}}\;0\;\min ) - \left( {{\mathrm{BA}}\;{\mathrm{peak}}\;{\mathrm{area}}\;{\mathrm{at}}\;5/30/60\;{\mathrm{min}}} \right)}}{{{\mathrm{BA}}\;{\mathrm{peak}}\;{\mathrm{area}}\;{\mathrm{at}}\;0\;{\mathrm{min}}}}\end{array}$$

*L. gasseri* whole-cell activity assays were carried out in a similar manner with identical reaction conditions. *L. gasseri* wild-type, Δ*bshA*, Δ*bshB* and Δ*bshAB* cultures were grown to mid-log, washed 3 times in PBS and diluted to a final concentration of OD_600_ = 0.1 in reactions containing AA-CDCA. After 1 h, 50 μl samples were taken and cells were quickly pelleted before 10 μl of supernatant was diluted into 90 μl methanol and stored at −80 °C. To calculate % deconjugation in the wild-type, Δ*bshA* or Δ*bshB* conditions, each conjugated BA was normalized to its abundance in the the Δ*bshAB* condition to control for the absorption/adsorption of BAs to the *L. gasseri* cell as follows:

2 $$\begin{array}{l}{\%}\;{\mathrm{Deconjugation}} = 100{\%} \times \\ \frac{{({\mathrm{BA}}\;{\mathrm{peak}}\;{\mathrm{area}}\;{\mathrm{in}}\;{{\Delta }}bshAB\;{\mathrm{condition}}) - \left( {{\mathrm{BA}}\;{\mathrm{peak}}\;{\mathrm{area}}\;{\mathrm{in}}\;{\mathrm{WT}}/{{\Delta }}bshA/{{\Delta }}bshB\;{\mathrm{condition}}} \right)}}{{{\mathrm{BA}}\;{\mathrm{peak}}\;{\mathrm{area}}\;{\mathrm{in}}\;{{\Delta }}bshAB\;{\mathrm{condition}}}}\end{array}$$

### Bile acid metabolomics

For metabolomic analysis of treated pre-FMT samples and murine intestinal contents, thawed ex vivo samples were homogenized using a genie disrupter (Scientific Industries) for 15 min after vortex mixing for 30 min. Samples were then centrifuged at 7,000 × *g* and 4 °C for 10 min and the supernatant was removed and used for analysis. Internal standard mixture (ITSD, 10 μl) and 10 μl of the extracted sample were added into the wells of a 96-well filter plate (PALL AcroPrep, PTFE 0.2 μm) fixed on top of a deep-well plate (Waters QuanRecovery) and extracted with 100 μl methanol by shaking at 600 r.p.m. for 20 min with a plate mixer (Thermo Fisher). Elution of the methanol extracts was performed using a positive-pressure manifold (Waters) into the lower receiving deep-well plate, which was then detached from the upper filter plate. After adding 50 μl MS-grade water to the extracts and shaking briefly (600 r.p.m., 5 min), samples from the microbial conjugated AA-BA pooled activity assays were injected (5 μl) without dilution or extraction.

For the quantification of the bile acids, an internal standard mixture of 16 deuterated bile acids (containing 6 unconjugated and 10 conjugated bile acids) was created by combining equal volumes of one vial of the unconjugated (Cambridge Isotope Laboratories, MSK-BA1-1) and one vial of the conjugated (Cambridge Isotope Laboratories, MSK-BA2-1) bile acids to make a 50 μM solution in 1.0 ml 1:1 methanol:water. This stock was further diluted and added in equal volumes (10 μl) to each sample, blank, calibration point and quality control at a final concentration of 10 μM. Calibration curves were prepared by making a mixture of 16 unlabelled bile acids (containing 6 unconjugated and 10 conjugated bile acids) by combining equal volumes of a vial of the unconjugated (Cambridge Isotope Laboratories, MSK-BA1-US-1) and a vial of the conjugated (Cambridge Isotope Laboratories, MSK-BA2-US-1) bile acids to make a 400 μM solution in 1:1 methanol:water. Serial dilutions were prepared to create a 9-point calibration curve ranging from 500 nM to 400 μM and using the concentrations 500 nM, 1 μM, 5 μM, 10 μM, 20 μM, 50 μM, 100 μM, 200 μM and 400 μM.

LC–IMS–MS analyses were performed using an Agilent 1290 Infinity UPLC system coupled with an Agilent 6560 IM-QTOF MS instrument. Chromatographic separation was achieved using a Restek Raptor C18 column (1.7 μm, 2.1 × 50 mm) heated to a temperature of 60 °C. Mobile phase A was composed of 5 mM ammonium acetate, while mobile phase B was 1:1 methanol:acetonitrile. The LC initially started at 0.5 ml min^−1^ and 15% B with isocratic elution for 2 min, followed by a stepped gradient going to 80% B over the next 7.7 min (15–35% B over 2 min, 35–40% B over 2 min, 40–50% B over 1.5 min, 50–55% B over 1.1 min, 55–80% B over 1.1 min). The flow rate and mobile phase composition were then increased to 0.8 ml min^−1^ and 85% B for an additional 0.8 min, followed by re-equilibration at initial conditions for 2 min, resulting in a total run time of 12.5 min. Samples were analysed at negative ion mode (capillary voltage 4,000 V, nebulizer gas pressure 40 psi, ion source temperature 325 °C, dry gas flow 10 l min^−1^) and data were collected from 50–1,700 *m*/*z* with an IMS drift potential of 17.2 V cm^−1^, frame rate of 0.09 frames per second, IMS transient rate of 16 IMS transients per frame, maximum IMS drift time of 60 ms and TOF transient rate of 600 transients per IMS transient. LC–IMS–MS spectra were acquired using MassHunter Acquisition Software and raw files (.d) were uploaded to Skyline-daily (v22.2) for peak picking and molecular annotation using a library built from the synthesized bile acid mixtures and standard mixtures. Peak areas per compound per sample were exported to Microsoft Excel and utilized for statistical analysis in GraphPad Prism. All LC–IMS–MS data are publicly available on Panorama under the Panorama dashboard of the Baker Lab-NCSU within the ‘0821 BSH Assessment’, ‘1121 Mouse Germination and Growth Ex-Vivo’ and the ‘1121 Theriot FMT Ex-Vivo Growth’ projects.

### Untargeted metabolomic analysis

Metabolomic analyses of untreated pre-FMT samples were performed by Metabolon in the same manner as described in our previous study^80^. Briefly, individual samples were subjected to methanol extraction and then split into aliquots for analysis by ultrahigh-performance liquid chromatography-mass spectrometry. The global biochemical profiling analysis comprised four unique arms consisting of reverse-phase chromatography positive-ionization methods optimized for hydrophilic compounds (LC–MS Pos Polar) and hydrophobic compounds (LC–MS Pos Lipid), and reverse-phase chromatography performed under negative-ionization conditions (LC–MS Neg) as well as a hydrophilic interaction liquid chromatography (HILIC) method coupled to negative ionization (LC–MS Polar). All the methods alternated between full-scan MS and data-dependent MS*n* scans. The scan ranges differed slightly between methods but generally covered 70–1,000 *m*/*z*.

Metabolites were identified by automated comparison of the ion features in the experimental samples to a reference library of chemical standard entries that included retention time, molecular weight (*m*/*z*), preferred adducts, in-source fragments as well as associated MS spectra, and were curated by visual inspection for quality control using software developed at Metabolon. Identification of known chemical entities was based on comparisons to metabolomic library entries of purified standards^81^.

### Statistical analyses

All statistical analysis was performed in GraphPad Prism 8 or 9. No statistical methods were used to pre-determine sample sizes, but our sample sizes are similar to those reported in previous publications. All data met the assumptions of the statistical tests used and data distribution was assumed to be normal in log_10_-transformed values but this was never formally tested. Data collection was not randomized and analysis was not blinded. No data were excluded from analysis. Specific activity assays were analysed as the average of *n* = 3 experiments with a two-way analysis of variance (ANOVA) with Tukey’s multiple comparisons test. Comparisons were made separately between the wild-type and mutant versions of LgasBSHa and LgasBSHb. Inhibition of *C. difficile* spore germination, membrane integrity and P_*tcdA*_-mCherry reporter assays were analysed from *n* = 3 experiments with a one-tailed Welch’s *t*-test. Statistical comparisons were only made between conditions containing related BAs (that is, TCA, GCA and CA). *C. difficile* growth and bile acid metabolomics in pre-FMT samples were analysed from *n* = 3 experiments using a one-way ANOVA with Sidak’s correction for multiple comparisons. In the cases where c.f.u. or bile acid metabolomic data were generated from intestinal contents that were split and treated with PBS or a BSH cocktail, one-tailed ratio paired *t*-tests were used to analyse findings. In vivo c.f.u.s were analysed with a Mann-Whitney test. Bile acid metabolomics from in vivo samples were analysed using a Kruskal-Wallis with Dunn’s test for multiple comparisons. All graphed bars represent mean ± s.d. Asterisks indicate significant differences (*P < 0.05, **P < 0.01, ***P < 0.001, ****P < 0.0001). See Reporting Summary and figure legends for more details.

### Reporting summary

Further information on research design is available in the Nature Portfolio Reporting Summary linked to this article.

## Supplementary information

Supplementary Information Supplementary Figs. 1–10.

Reporting Summary

Peer Review File

Supplementary Tables 1–6 Table 1: Lactobacillaceae BSH cluster and phylogeny information. Table 2: Purified BSH sequences and information. Table 3: Mutagenesis primers. Table 4: IGC sequences and selectivity loop. Table 5: CD analysis. Table 6: X-ray crystallography statistics.

Supplementary Data 1 *P* values from all statistics.

Supplementary Data 2 7SVE PDB validation report.

Supplementary Data 3 7SVF PDB validation report.

Supplementary Data 4 7SVG PDB validation report.

Supplementary Data 5 7SVH PDB validation report.

Supplementary Data 6 7SVI PDB validation report.

Supplementary Data 7 7SVJ PDB validation report.

Supplementary Data 8 7SVK PDB validation report.

## Data Availability

All data associated with this study are available in the main text or the supplementary materials. All PDB accession codes used or generated by this study are listed here: 2HEZ, 2BJF, 2HF0, 4WL3, 6UFY, 5HKE, 7SVE, 7SVF, 7SVG, 7SVH, 7SVI, 7SVJ and 7SVK. The Integrated Gene Catalog dataset file used in this study is available at https://ftp.cngb.org/pub/gigadb/pub/10.5524/100001_101000/100064/1.GeneCatalogs/IGC.pep.gz. Source data are provided with this paper.

## Source data

Source Data Fig. 1 BSH specific activity data.
Source Data Fig. 2 BSH specific activity data.
Source Data Fig. 3 *C. difficile* germination, MIC and c.f.u. data with BA metabolomics.
Source Data Fig. 4 *C. difficile* germination and c.f.u. data with BA metabolomics.
Source Data Fig. 5 *C. difficile* germination, c.f.u., growth and toxin reporter data with bile acid metabolomics and BSH activity data.
Source Data Extended Data Fig. 2 *C. difficile* germination, membrane integrity and toxin reporter data.
Source Data Extended Data Fig. 3 Pre-FMT sample bile acid metabolomics and antibiotics.
Source Data Extended Data Fig. 4 *C. difficile* c.f.u.s.
Source Data Extended Data Fig. 5 Mouse intestinal bile acid metabolomics.
Source Data Extended Data Fig. 6 Mouse intestinal bile acid metabolomics.
Source Data Extended Data Fig. 7 BSH specific activity data.
Source Data Extended Data Fig. 8. BSH activity data.
Source Data Extended Data Fig. 9 BSH activity kinetics.
Source Data Extended Data Fig. 10 *C. difficile* germination and growth.

## Code availability

All custom code is available upon request.
