# Supplementary material for: Bile salt hydrolases shape the bile acid landscape and restrict Clostridioides difficile growth in the murine gut
Source: Nat Microbiol. 2023 Mar 13;8(4):611–28. doi: 10.1038/s41564-023-01337-7 (PMC10066039; doi:10.1038/s41564-023-01337-7)
Supplement: Supplementary file 1 — Supplementary Figs. 1–10. [file 41564_2023_1337_MOESM1_ESM.pdf]

nature microbiology

Article https://doi.org/10.1038/s41564-023-01337-7

Bile salt hydrolases shape the bile acid landscape and restrict *Clostridioides difficile* growth in the murine gut

In the format provided by the authors and unedited

This Supplementary Information file includes:

Fig. S1 to S10

Supplemental Figures

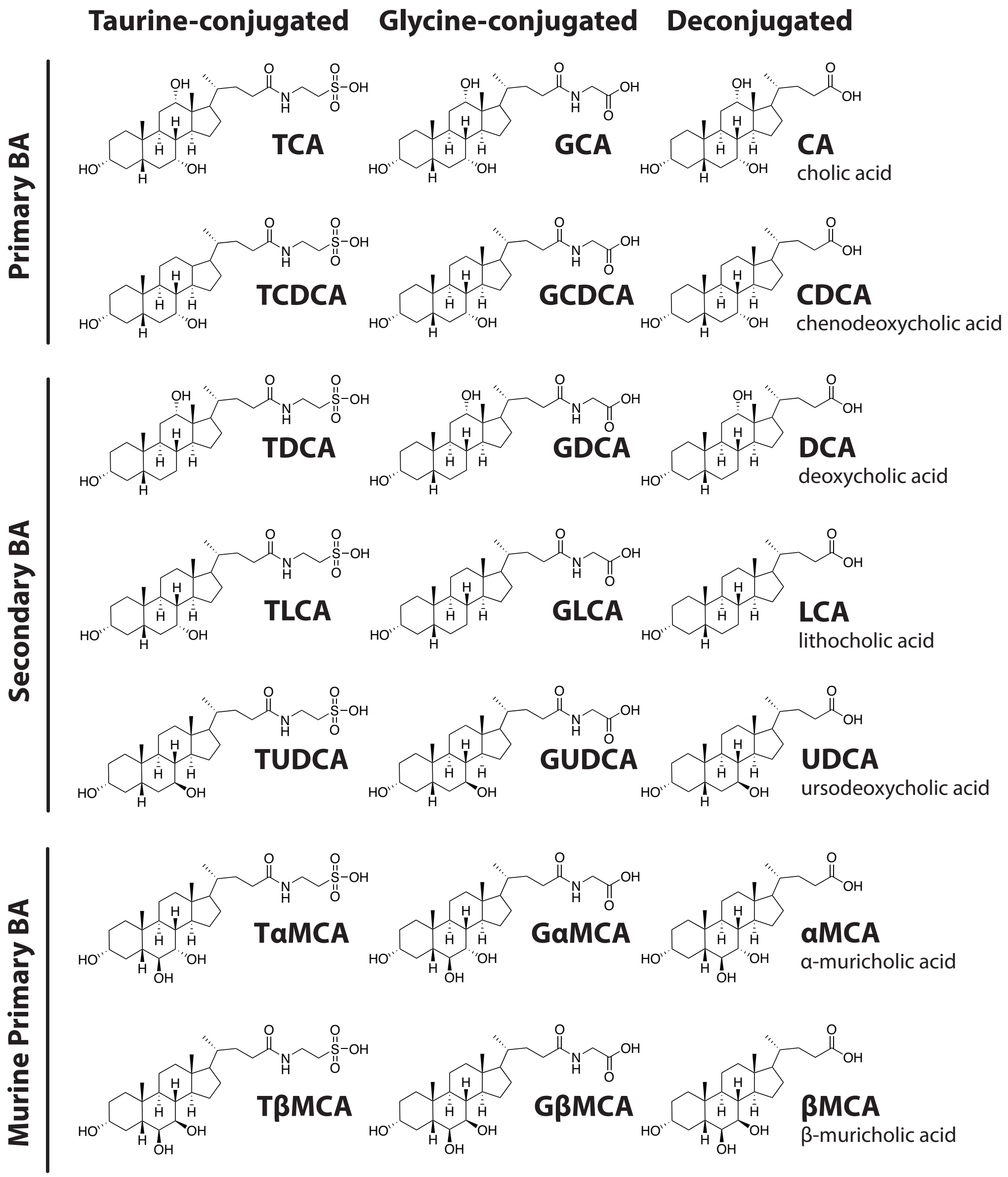

Figure S1. Bile acid structures and abbreviations.

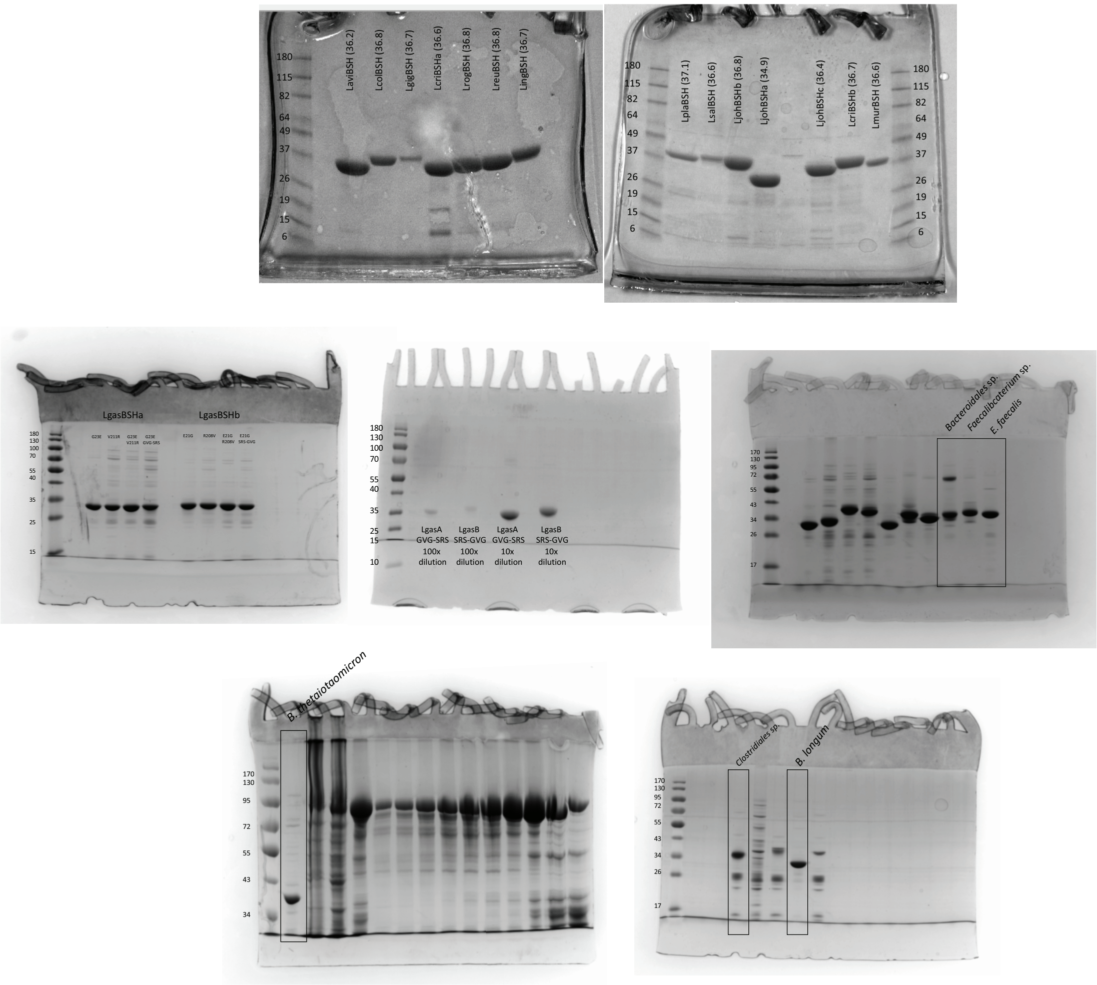

Figure S2. SDS-PAGE of purified *Lactobacillaceae* BSHs. Protein ladder sizes noted in kDa to the left of gels. BSHs were purified from a single batch each.

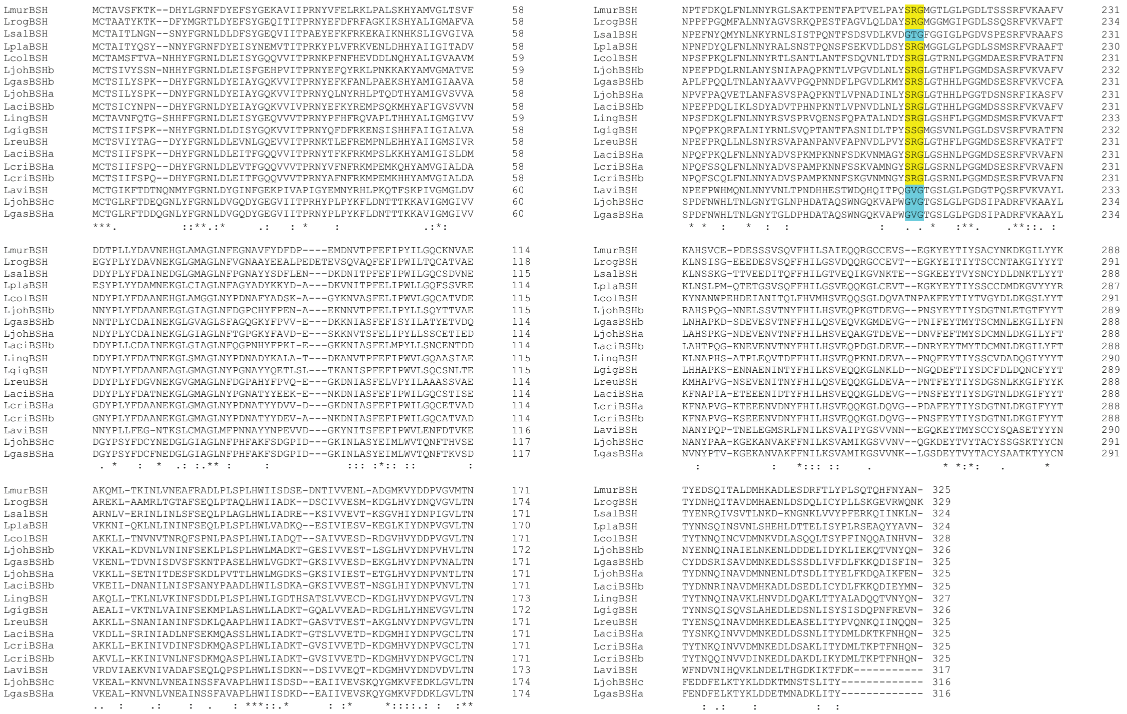

Figure S3. BSH multiple sequence alignment. Multiple sequence alignment of the *Lactobacillaceae* BSH proteins examined here. The three-residue selectivity region is highlighted in yellow (glycine-preferring enzymes) or blue (taurine-preferring enzymes). Created using the ClustalOmega Multiple Sequence Alignment tool.

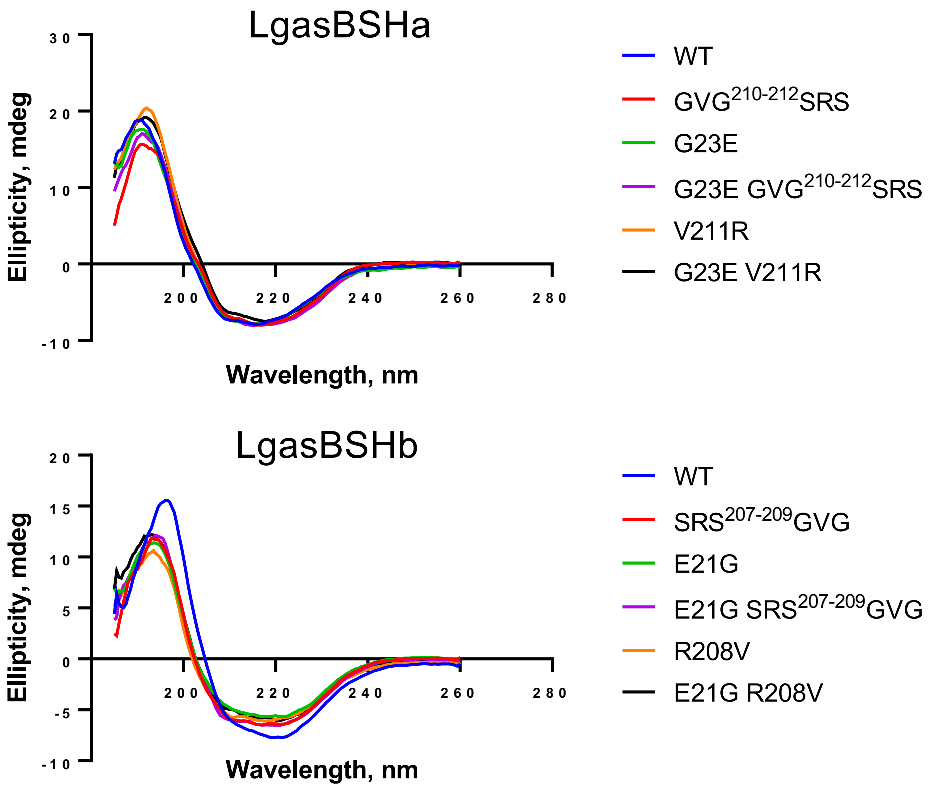

Figure S4. Circular dichroism of LgasBSH mutants. CD spectra of LgasBSHa (top) and LgasBSHb (bottom) with their respective mutants. A background spectrum of buffer alone was acquired and subtracted out to correct for background signal.

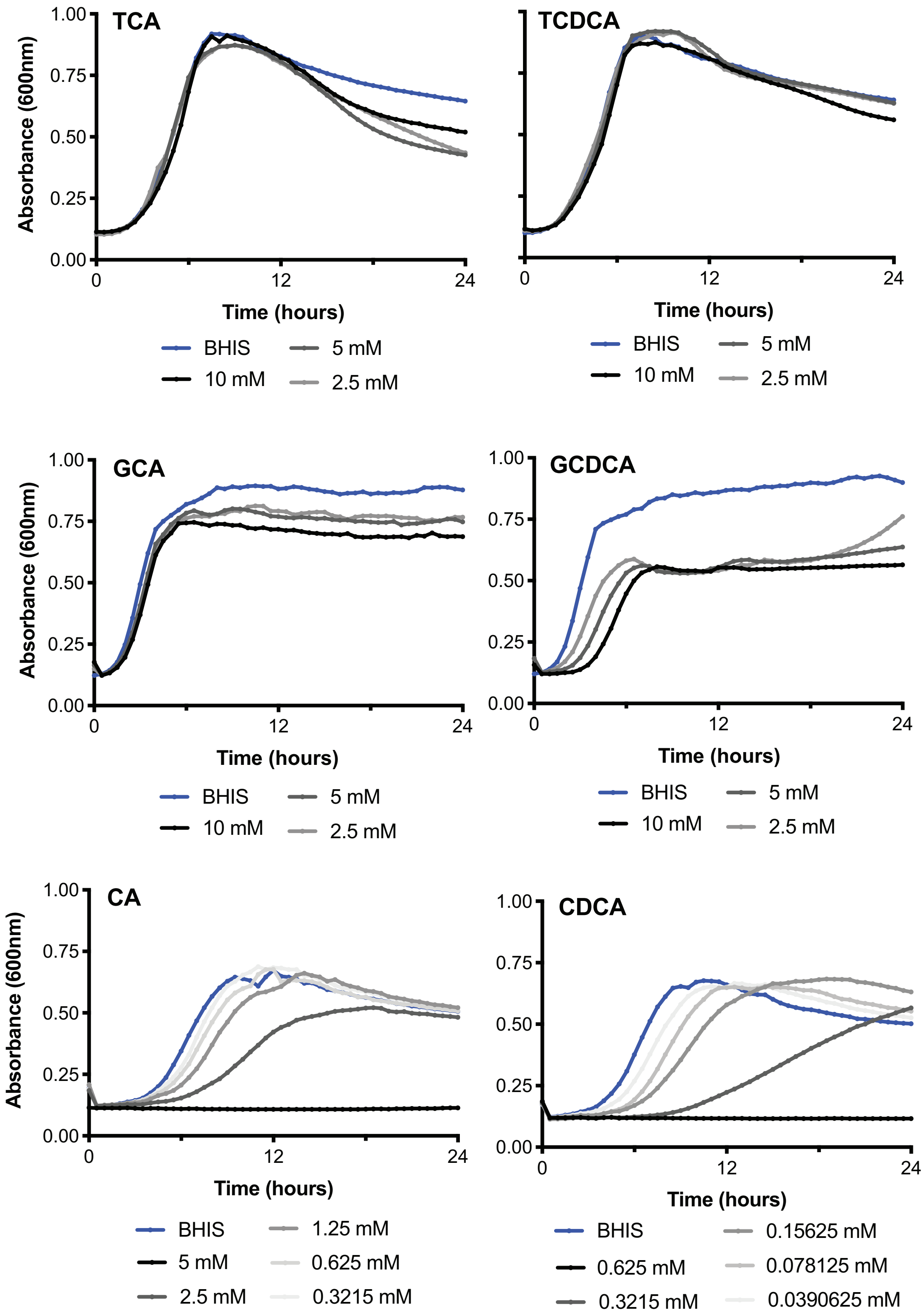

Figure S5. ***C. difficile*** **growth in the presence of various concentrations of CA and CDCA bile acids.**

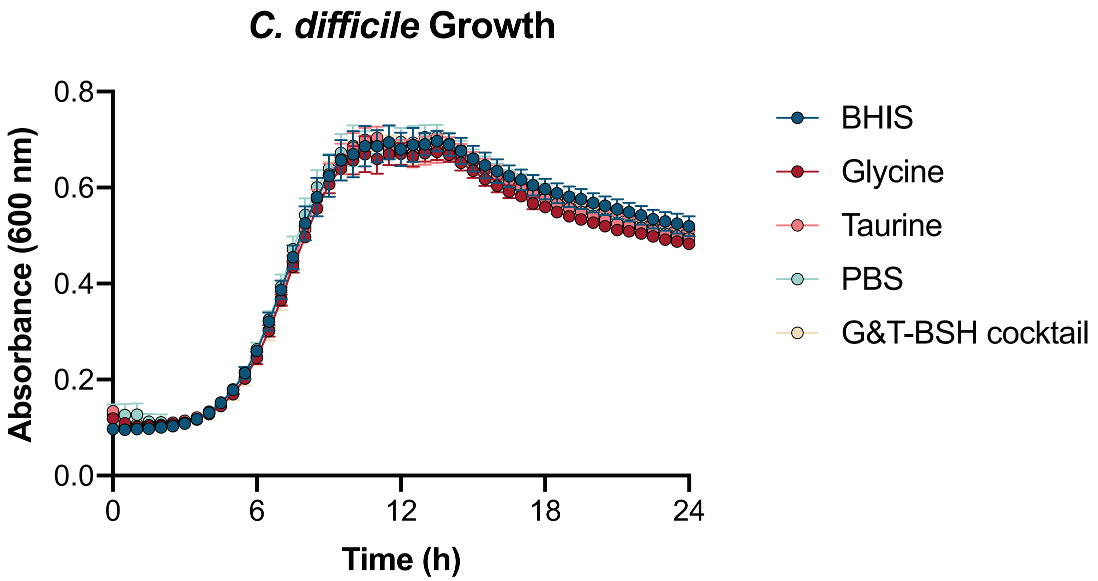

Figure S6. *C. difficile* growth with BSH cocktail. *C. difficile* growth is not impacted by the amino acids (4.5 mM each) or the BSH cocktail. Curves represent mean (n = 3) ± standard deviation.

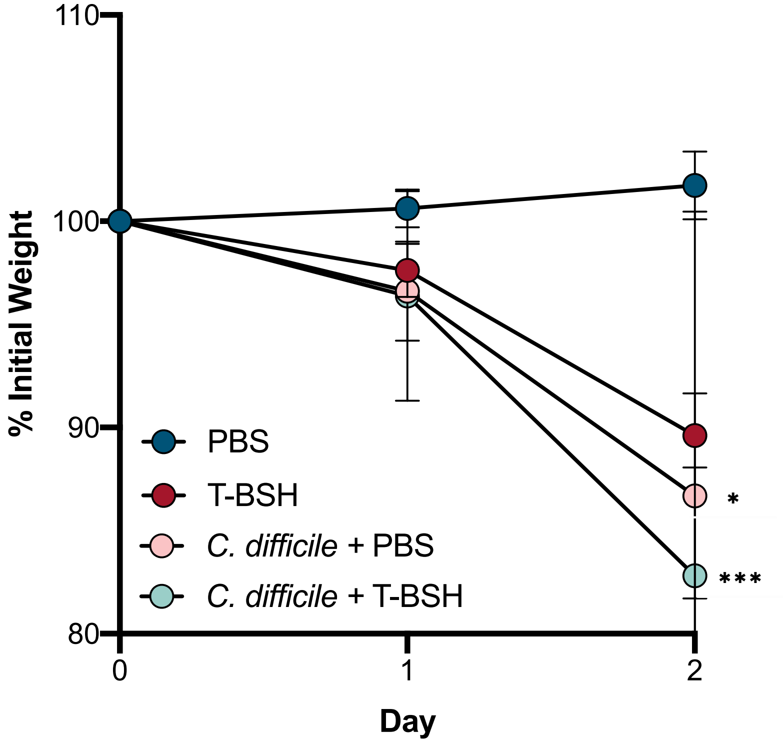

Figure S7. Changes in mouse weight after *C. difficile* challenge. Data points represent mean weight change relative to Day 0 (PBS: n = 6, BSH: n = 9, Cd + PBS = 10, Cd + BSH: n = 13) ± standard deviation. Asterisks indicate significant differences (*$p < 0.05$, ***$p < 0.001$) between treatments and the PBS-treated mice by Kruskal-Wallis test with Dunn's test for multiple comparisons. All p values listed in **Supplementary Data 1**.

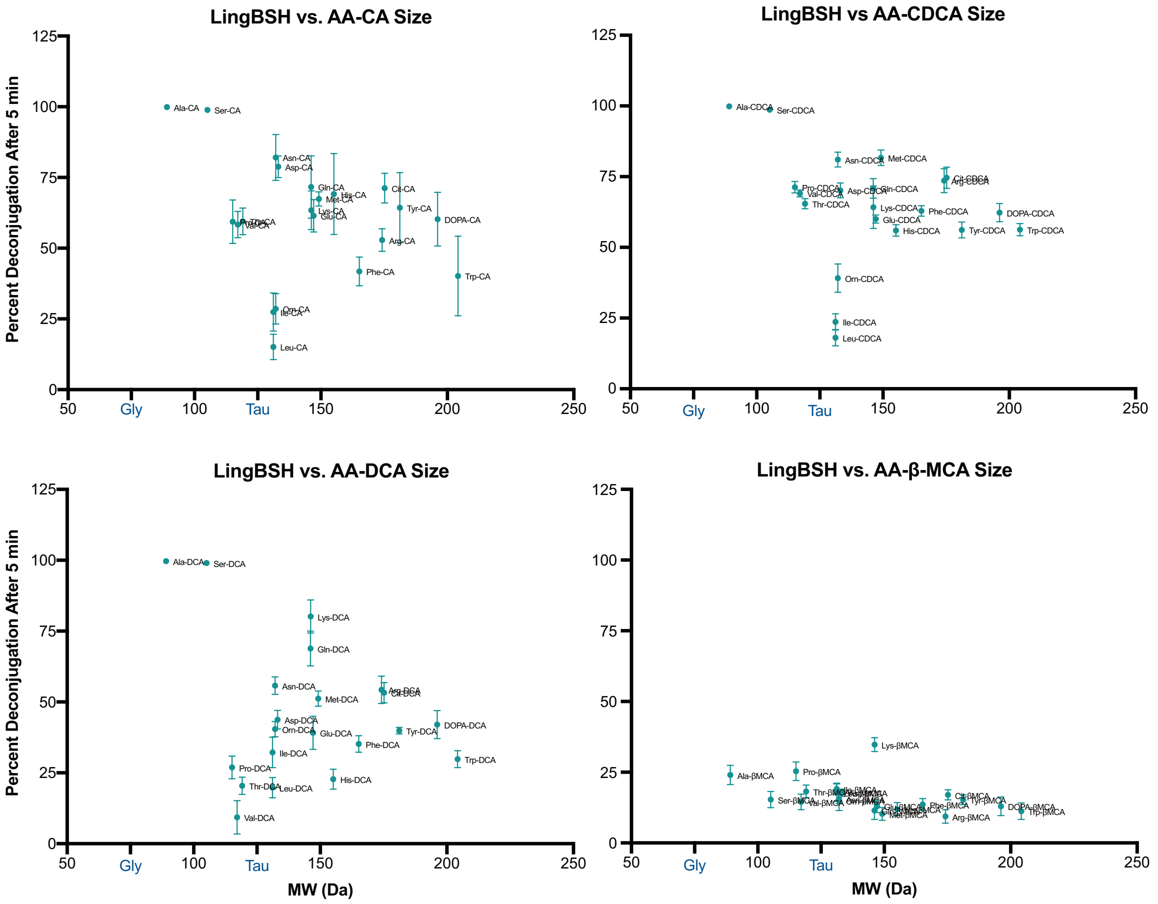

Figure S8. LingBSH activity compared to MCBA amino acid size. Initial activity of LingBSH with AA-BA mixtures. All dots represent the mean deconjugation from a n = 3 replicates ± standard deviation. individual MCBAs. The MW of Gly and Tau are indicated on the x-axis for reference.

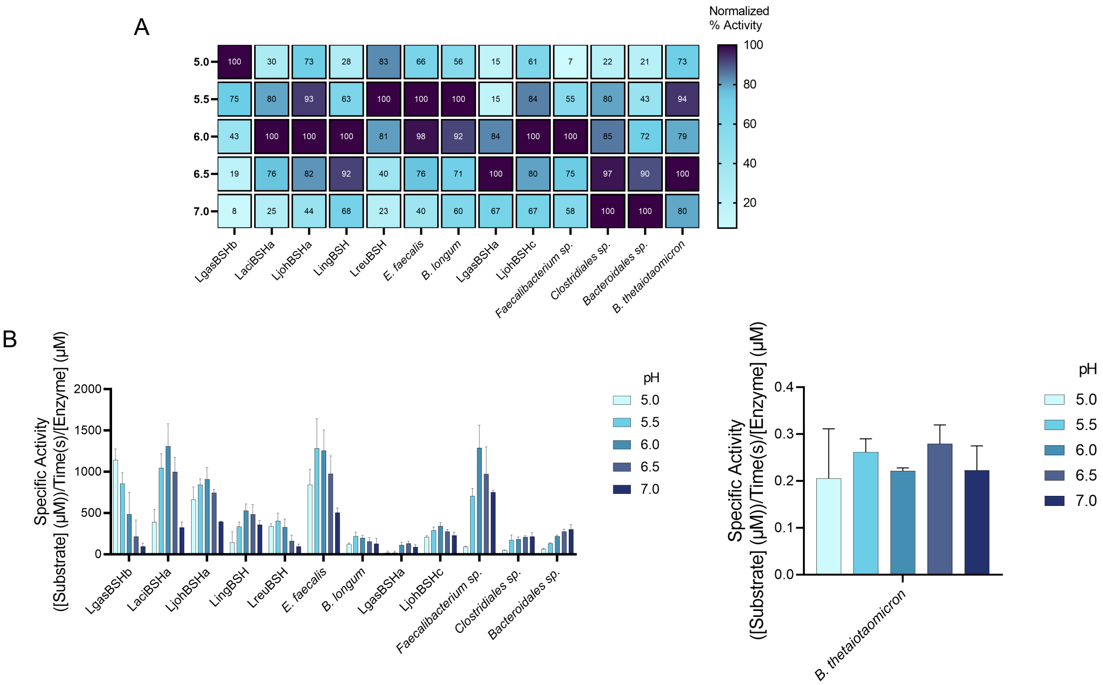

Figure S9. BSH specific activity at various pHs. The activity of each enzyme was tested at 5 pHs, and the optimal pH for activity was used for all future assays. Activity was determined with either GCA or TCA, whichever demonstrated faster processing (See Fig. 2F). A) Normalized rates of activity for each enzyme among 5 pH values. Normalization was performed by defining the highest specific activity to be 100%, and 0 activity to be 0%. B) Specific activity values for each enzyme at each pH. Final specific activity values are the average of n=3 biological replicates ± SD.

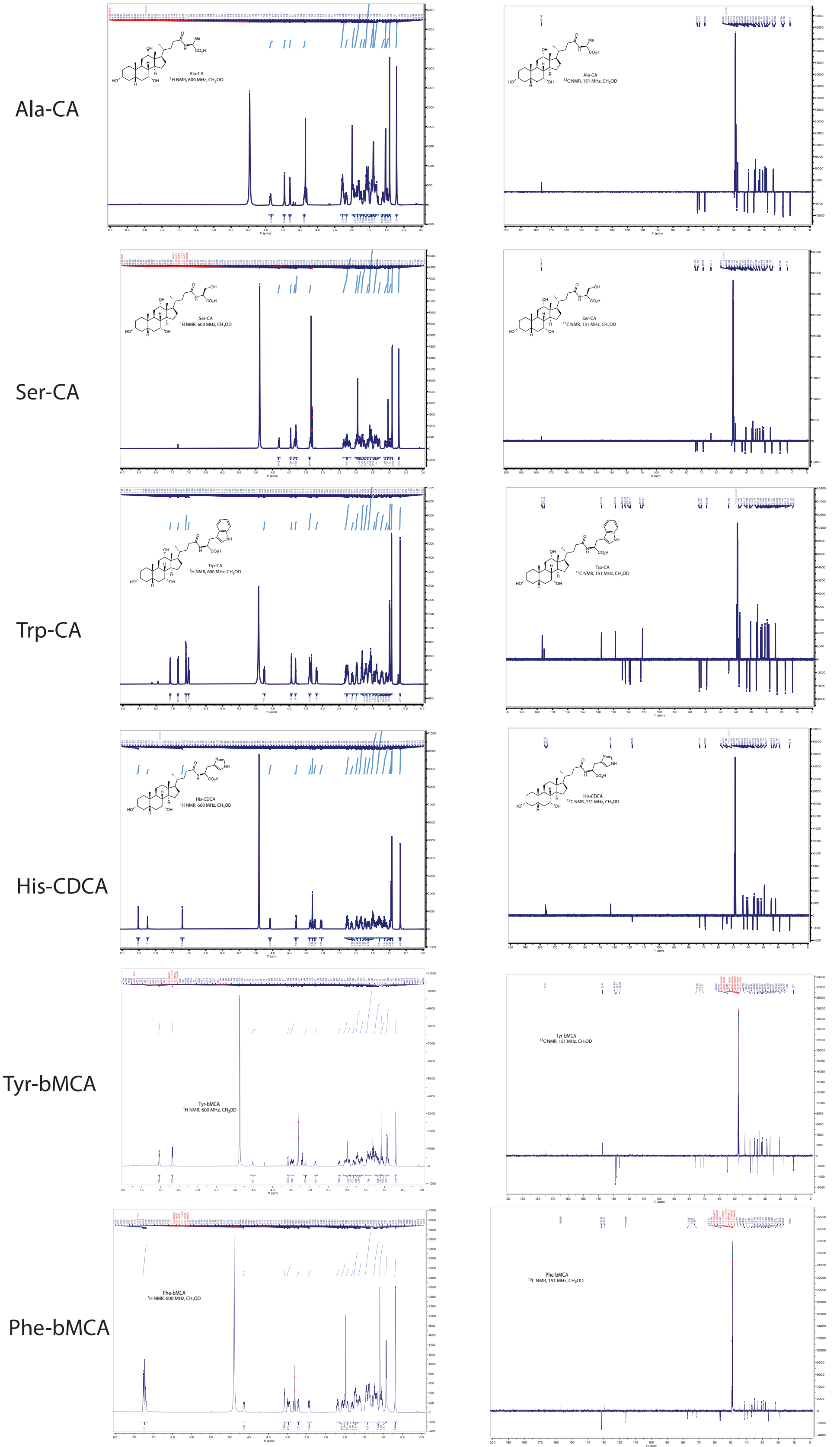

Figure S10. ¹H and ¹3C NMR spectra of pure synthesized conjugated bile acids.
