# Supplementary material for: Bile salt hydrolases shape the bile acid landscape and restrict Clostridioides difficile growth in the murine gut
Source: Nat Microbiol. 2023 Mar 13;8(4):611–28. doi: 10.1038/s41564-023-01337-7 (PMC10066039; doi:10.1038/s41564-023-01337-7)
Supplement: Supplementary file 2 — Reporting Summary [file 41564_2023_1337_MOESM2_ESM.pdf]

# nature portfolio

Corresponding author(s): Casey M. Theriot, Rodolphe Barrangou, Matthew R. Redinbo

Last updated by author(s): Jan 30, 2023

# Reporting Summary

Nature Portfolio wishes to improve the reproducibility of the work that we publish. This form provides structure for consistency and transparency in reporting. For further information on Nature Portfolio policies, see our Editorial Policies and the Editorial Policy Checklist.

## Statistics

For all statistical analyses, confirm that the following items are present in the figure legend, table legend, main text, or Methods section.

| n/a | Confirmed | |
|---|---|---|
| ☐ | ☒ | The exact sample size (*n*) for each experimental group/condition, given as a discrete number and unit of measurement |
| ☐ | ☒ | A statement on whether measurements were taken from distinct samples or whether the same sample was measured repeatedly |
| ☐ | ☒ | The statistical test(s) used AND whether they are one- or two-sided<br>*Only common tests should be described solely by name; describe more complex techniques in the Methods section.* |
| ☒ | ☐ | A description of all covariates tested |
| ☐ | ☒ | A description of any assumptions or corrections, such as tests of normality and adjustment for multiple comparisons |
| ☐ | ☒ | A full description of the statistical parameters including central tendency (e.g. means) or other basic estimates (e.g. regression coefficient) AND variation (e.g. standard deviation) or associated estimates of uncertainty (e.g. confidence intervals) |
| ☐ | ☒ | For null hypothesis testing, the test statistic (e.g. *F*, *t*, *r*) with confidence intervals, effect sizes, degrees of freedom and *P* value noted<br>*Give P values as exact values whenever suitable.* |
| ☒ | ☐ | For Bayesian analysis, information on the choice of priors and Markov chain Monte Carlo settings |
| ☒ | ☐ | For hierarchical and complex designs, identification of the appropriate level for tests and full reporting of outcomes |
| ☒ | ☐ | Estimates of effect sizes (e.g. Cohen's *d*, Pearson's *r*), indicating how they were calculated |

*Our web collection on statistics for biologists contains articles on many of the points above.*

## Software and code

Policy information about availability of computer code

| | |
|---|---|
| Data collection | Figure 1B: BSH sequence data was collected froom NCBI in July 2021.<br>Figure 1C, 2F, 2I, ED 7: BSH activity data was collected using Magellan 7.2.<br>Figure 1D: X-ray detector software was automatically run at APS as part of the GMCA automated data processing pipeline to integrate and scale crystallography data.<br>Figure 3F, 4D-E, 5B-E, ED 3-6, ED 8-9: LC-IMS-MS metabolomic data was collected and visualized with IM-MS Browser 10.0<br>Figure 5H, ED 2B, ED 2C: Membrane integrity and PtcdA-mCherry reporter assay data was collected using Magellan 7.2.<br>Figure 5G, ED 10C, S5, S6: Growth kinetics were captured using Magellan 7.2<br>Figure S10: NMR data collected using Bruker TopSpin 3.6.0. |
| Data analysis | Figure 1B: BSH sequences were analyzed using open-source software BLAST 2.6.0 and tool suite, MUSCLE aligner 3.8.2, HMMER 3.3.2, CDHIT 4.8.1, and custom python and bash scripts. Geneious Prime version 2022.1 and CLC Genomics Workbench version 12 were used for protein sequence alignments, construction of phylogenetic trees and addition of metadata.<br>Figure 1C, 2F, 2I-L, ED 7, S9: BSH specific activity assays were analyzed as the average of n=3-4 experiments and data was analyzed in Excel 16.39. In Figure 1D: Crystallographic data was analyzed using Phenix version 1.17.1-3660 to perform molecular replacement (Phaser) and structure refinement (Autobuild, phenix.refine) of crystal structures. Coot version 0.9.4.1 was used for manual inspection and adjustment of structure models.<br>Figure 2E: A sequence similarity network was generated using the free online Enzyme Function Initiative – Enzyme Similarity Tool (https://efi.igb.illinois.edu/efi-est/). Custom code was used for the structural BSH metagenomics.<br>Figure 2F, statistical comparisons were made using a two-way ANOVA with Tukey's multiple comparisons test. Comparisons were made separately between the wild type and mutant versions of LgasBSHa and LgasBSHb.<br>Figure 3B, 5F, ED 2A-B, 10B: Inhibition of C. difficile spore germination and membrane integrity assays was analyzed from an n=3 experiments and comparisons were only made between BAs that share the same sterol core (i.e. TCA, GCA, CA) using a one-way ANOVA with Tukey's multiple comparisons test except for the αMCAs and βMCAs which were compared with a one-tailed Welch's t test.<br>Figure 3F, 4D-E, 5B-E, ED 3-6, ED 8-9, S8: Metabolomic data was analzyed, and validated using Skyline-daily 22.2 |

Figure 3E-F, ED 4: C. difficile CFU and bile acid metabolomic data generated from preFMT samples were analyzed with a one-way ANOVA with Sidak's multiple comparisons test.
Figure 4B-E, 5B-C, ED 5, S7: C. difficile spore germination, CFU, and bile acid metabolomic data was generated from ex vivo mouse intestinal contents from an n=4 mice that were split and treated with PBS or a BSH cocktail, one-tailed ratio paired t tests or were used to analyze findings. In vivo C. difficile CFUs were compared from n=6-13 mice using a Mann-Whitney whereas mouse weight and bile acid metabolomic data was compared by a Kruskal-Wallis with Dunn's multiple comparisons test.
Figure 5H, ED 2C: PtcdA-mCherry reporter assays were analyzed from an n=4 experiments and comparisons were performed compared to BHIS or MeOH (BHIS media with methanol) based on the BA's solvent using a one-way ANOVA with Dunnett's multiple comparisons test.
Figure S4: CD data was analyzed using the online server DichroWeb.
Figure S10: NMR data was analyzed using MNova 14.2.0.
All statistical analysis was performed in GraphPad Prism 8 or 9. All graphed bars represent mean ± standard deviation. Asterisks indicate significant differences (*p < 0.05, **p < 0.01, ***p < 0.001, ****p < 0.0001).

For manuscripts utilizing custom algorithms or software that are central to the research but not yet described in published literature, software must be made available to editors and reviewers. We strongly encourage code deposition in a community repository (e.g. GitHub). See the Nature Portfolio guidelines for submitting code & software for further information.

## Data

Policy information about availability of data

All manuscripts must include a data availability statement. This statement should provide the following information, where applicable:
- Accession codes, unique identifiers, or web links for publicly available datasets
- A description of any restrictions on data availability
- For clinical datasets or third party data, please ensure that the statement adheres to our policy

All data associated with this study are available in the main text or the Supplementary Materials.

# Field-specific reporting

Please select the one below that is the best fit for your research. If you are not sure, read the appropriate sections before making your selection.

☒ Life sciences ☐ Behavioural & social sciences ☐ Ecological, evolutionary & environmental sciences

For a reference copy of the document with all sections, see nature.com/documents/nr-reporting-summary-flat.pdf

# Life sciences study design

All studies must disclose on these points even when the disclosure is negative.

| | |
|---|---|
| Sample size | Sample sizes were selected based on published results in the field and preliminary experimentation. No samples size calculation was performed.<br><br>Specific activity assays were performed using n=3-4 independent experiments Inhibition of C. difficile spore germination, growth, and membrane integrity assays was analyzed from an n=3 independent experiments. PtcdA-mCherry reporter assays were analyzed from an n=4 independent experiments.<br><br>C. difficile CFUs and bile acid metabolomics ex vivo pre-FMT samples were from n=6 individual patients and these experiments were performed in triplicate within each sample. C. difficile CFU and bile acid metabolomic data generated from ex vivo mouse small intestinal and cecal contents were from n=4 mice. Contents were split and treated with PBS or a BSH cocktail. In vivo C. difficile CFUs were compared from n=6-13 mice depending on the treatment. The number of mice used in this study was n=6-13 per group and was necessary for statistical power and to control for cage-to cage variation. See methods and all figure legends for more details. |
| Data exclusions | No data were excluded from our study. |
| Replication | Attempts at replication were successful for all experiments. Data points in figures from all biological replicates are displayed.<br><br>Replication of the in vivo mouse experiment in Fig. 5 was performed successfully. An initial experiment was performed with a smaller sample size of mice (n=4) that demonstrated the BSH cocktail could significantly lower C. difficile CFUs in vivo. Those data were neither grouped into the data in Fig. 5B nor were they shown. Fig. 5B only displays the data collected from the replication of this experiment that used a larger sample size of mice. |
| Randomization | Mice were assigned a treatment group at random upon arrival.<br>All preFMT samples and mouse gut content samples were randomly selected and run for ex vivo assays with BSHs and C. difficile at two timepoints. All mice were selected for necropsy from different cages post C. difficile challenge to account for cage to cage variation. |
| Blinding | We were not blinded to the samples in this study when running all experiments.We were not blinded to the groups of mice in this study to prevent cross contamination. Additionally, C. difficile infected mice exhibit clinical signs which are quite obvious (lethargy, hunched posture, ruffled fur, wet fecal pellets decorating the cage walls, conjunctivitis, etc.). We were blinded to all downstream analysis including bacterial enumeration measurments and omic analysis as well as rCDI patient data. |

# Reporting for specific materials, systems and methods

We require information from authors about some types of materials, experimental systems and methods used in many studies. Here, indicate whether each material, system or method listed is relevant to your study. If you are not sure if a list item applies to your research, read the appropriate section before selecting a response.

## Materials & experimental systems

| n/a | Involved in the study |
| --- | --- |
| ☒ | ☐ Antibodies |
| ☒ | ☐ Eukaryotic cell lines |
| ☒ | ☐ Palaeontology and archaeology |
| ☐ | ☒ Animals and other organisms |
| ☐ | ☒ Human research participants |
| ☒ | ☐ Clinical data |
| ☒ | ☐ Dual use research of concern |

## Methods

| n/a | Involved in the study |
| --- | --- |
| ☒ | ☐ ChIP-seq |
| ☒ | ☐ Flow cytometry |
| ☒ | ☐ MRI-based neuroimaging |

## Animals and other organisms

Policy information about studies involving animals; ARRIVE guidelines recommended for reporting animal research

| | |
| --- | --- |
| Laboratory animals | Male and female mice were housed in a room with an average temperature of 70F and 35% humidity. C57BL/6J WT mice (5 weeks old) were purchased from Jackson Labs. |
| Wild animals | This study did not involve wild animals. |
| Field-collected samples | This study did not involve samples collected from the field. |
| Ethics oversight | All animal work was approved by NC State's Institutional Animal Care and Use Committee (IACUC). |

Note that full information on the approval of the study protocol must also be provided in the manuscript.

## Human research participants

Policy information about studies involving human research participants

| | |
| --- | --- |
| Population characteristics | We were blinded to patient population characteristics |
| Recruitment | All consenting patients undergoing FMT for rCDI at the University of North Carolina from January to December 2017 in a prospective registry were enrolled for fecal collection. rCDI was defined by a patient having at least the third episode of CDI. There were no exclusion criteria for participation in the registry specifically, though subjects were by definition undergoing FMT under the care of a physician who judged the benefits to outweigh the risks. Patient stool samples from 2 weeks prior to FMT (pre-FMT) were collected. The study was approved by the UNC IRB (#16-2283). Informed written consent was obtained from recipients. Stool samples were collected and de-identified by the research team. |
| Ethics oversight | UNC IRB (#16-2283) |

Note that full information on the approval of the study protocol must also be provided in the manuscript.
