# Supplementary material for: Bile salt hydrolases shape the bile acid landscape and restrict Clostridioides difficile growth in the murine gut
Source: Nat Microbiol. 2023 Mar 13;8(4):611–28. doi: 10.1038/s41564-023-01337-7 (PMC10066039; doi:10.1038/s41564-023-01337-7)
Supplement: Supplementary file 6 — 7SVE PDB validation report. [file 41564_2023_1337_MOESM6_ESM.pdf]

Full wwPDB X-ray Structure Validation Report (i)

Nov 22, 2021 – 11:24 AM EST

PDB ID	:	7SVE
Title	:	Bile Salt Hydrolase A from Lactobacillus acidophilus
Deposited on	:	2021-11-19
Resolution	:	2.15 Å(reported)

This is a Full wwPDB X-ray Structure Validation Report.

This report is produced by the wwPDB biocuration pipeline after annotation of the structure.

We welcome your comments at *validation@mail.wwpdb.org*
A user guide is available at
https://www.wwpdb.org/validation/2017/XrayValidationReportHelp
with specific help available everywhere you see the (i) symbol.

CONFIDENTIAL VALIDATION REPORT

The following versions of software and data (see references (i)) were used in the production of this report:

MolProbity	:	4.02b-467
Xtriage (Phenix)	:	1.13
EDS	:	2.23.2
Percentile statistics	:	20191225.v01 (using entries in the PDB archive December 25th 2019)
Refmac	:	5.8.0158
CCP4	:	7.0.044 (Gargrove)
Ideal geometry (proteins)	:	Engh & Huber (2001)
Ideal geometry (DNA, RNA)	:	Parkinson et al. (1996)
Validation Pipeline (wwPDB-VP)	:	2.23.2

1 Overall quality at a glance ⓘ

The following experimental techniques were used to determine the structure:
X-RAY DIFFRACTION

The reported resolution of this entry is 2.15 Å.

Percentile scores (ranging between 0-100) for global validation metrics of the entry are shown in the following graphic. The table shows the number of entries on which the scores are based.

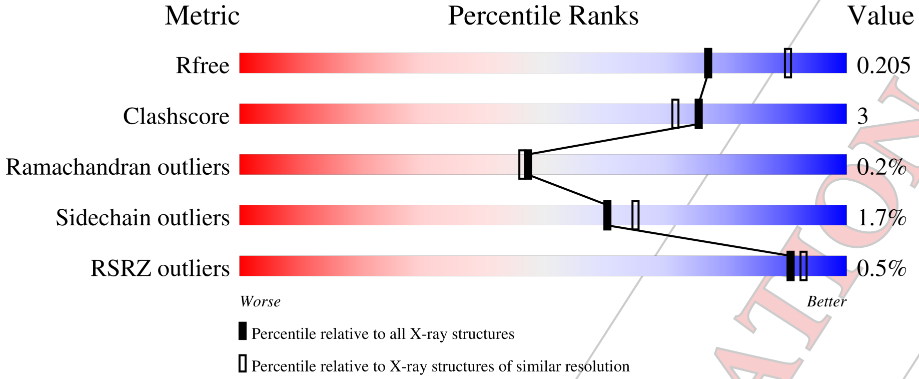

Metric	Whole archive (#Entries)	Similar resolution (#Entries, resolution range(Å))
R_{free}	130704	1479 (2.16-2.16)
Clashscore	141614	1585 (2.16-2.16)
Ramachandran outliers	138981	1560 (2.16-2.16)
Sidechain outliers	138945	1559 (2.16-2.16)
RSRZ outliers	127900	1456 (2.16-2.16)

The table below summarises the geometric issues observed across the polymeric chains and their fit to the electron density. The red, orange, yellow and green segments of the lower bar indicate the fraction of residues that contain outliers for >=3, 2, 1 and 0 types of geometric quality criteria respectively. A grey segment represents the fraction of residues that are not modelled. The numeric value for each fraction is indicated below the corresponding segment, with a dot representing fractions <=5% The upper red bar (where present) indicates the fraction of residues that have poor fit to the electron density. The numeric value is given above the bar.

Mol	Chain	Length	Quality of chain
1	A	331	87% 11% •
1	B	331	% 89% 8% • •
1	C	331	% 89% 9% •
1	D	331	% 90% 8% •

CONFIDENTIAL VALIDATION REPORT

2 Entry composition (i)

There are 2 unique types of molecules in this entry. The entry contains 11181 atoms, of which 0 are hydrogens and 0 are deuteriums.

In the tables below, the ZeroOcc column contains the number of atoms modelled with zero occupancy, the AltConf column contains the number of residues with at least one atom in alternate conformation and the Trace column contains the number of residues modelled with at most 2 atoms.

- Molecule 1 is a protein called Choloylglycine hydrolase.

Mol	Chain	Residues	Atoms					ZeroOcc	AltConf	Trace
1	A	324	Total 2596	C 1648	N 425	O 509	S 14	0	1	0
1	B	324	Total 2584	C 1638	N 425	O 507	S 14	0	1	0
1	C	325	Total 2611	C 1654	N 431	O 512	S 14	0	2	0
1	D	324	Total 2588	C 1641	N 424	O 510	S 13	0	1	0

There are 24 discrepancies between the modelled and reference sequences:

Chain	Residue	Modelled	Actual	Comment	Reference
A	326	HIS	-	expression tag	UNP G1U9U5
A	327	HIS	-	expression tag	UNP G1U9U5
A	328	HIS	-	expression tag	UNP G1U9U5
A	329	HIS	-	expression tag	UNP G1U9U5
A	330	HIS	-	expression tag	UNP G1U9U5
A	331	HIS	-	expression tag	UNP G1U9U5
B	326	HIS	-	expression tag	UNP G1U9U5
B	327	HIS	-	expression tag	UNP G1U9U5
B	328	HIS	-	expression tag	UNP G1U9U5
B	329	HIS	-	expression tag	UNP G1U9U5
B	330	HIS	-	expression tag	UNP G1U9U5
B	331	HIS	-	expression tag	UNP G1U9U5
C	326	HIS	-	expression tag	UNP G1U9U5
C	327	HIS	-	expression tag	UNP G1U9U5
C	328	HIS	-	expression tag	UNP G1U9U5
C	329	HIS	-	expression tag	UNP G1U9U5
C	330	HIS	-	expression tag	UNP G1U9U5
C	331	HIS	-	expression tag	UNP G1U9U5
D	326	HIS	-	expression tag	UNP G1U9U5
D	327	HIS	-	expression tag	UNP G1U9U5
D	328	HIS	-	expression tag	UNP G1U9U5

Continued on next page...

CONFIDENTIAL VALIDATION REPORT

Continued from previous page...

Chain	Residue	Modelled	Actual	Comment	Reference
D	329	HIS	-	expression tag	UNP G1U9U5
D	330	HIS	-	expression tag	UNP G1U9U5
D	331	HIS	-	expression tag	UNP G1U9U5

- Molecule 2 is water.

Mol	Chain	Residues	Atoms	ZeroOcc	AltConf
2	A	207	Total O<br>207 207	0	0
2	B	177	Total O<br>177 177	0	0
2	C	211	Total O<br>211 211	0	0
2	D	207	Total O<br>207 207	0	0

CONFIDENTIAL VALIDATION REPORT

3 Residue-property plots ⓘ

These plots are drawn for all protein, RNA, DNA and oligosaccharide chains in the entry. The first graphic for a chain summarises the proportions of the various outlier classes displayed in the second graphic. The second graphic shows the sequence view annotated by issues in geometry and electron density. Residues are color-coded according to the number of geometric quality criteria for which they contain at least one outlier: green = 0, yellow = 1, orange = 2 and red = 3 or more. A red dot above a residue indicates a poor fit to the electron density (RSRZ > 2). Stretches of 2 or more consecutive residues without any outlier are shown as a green connector. Residues present in the sample, but not in the model, are shown in grey.

- Molecule 1: Choloylglycine hydrolase

Chain A:

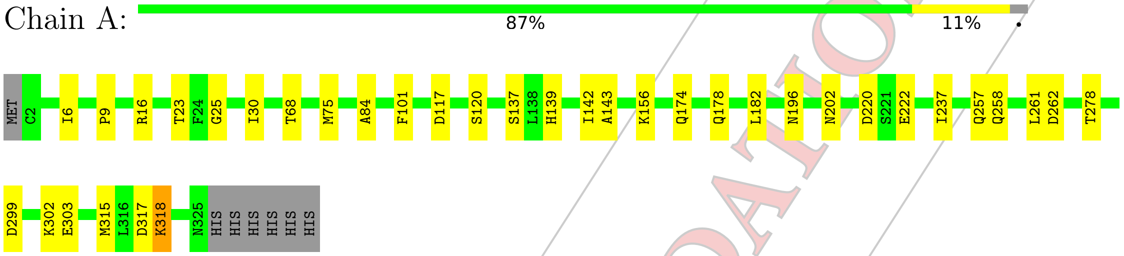

- Molecule 1: Choloylglycine hydrolase

Chain B:

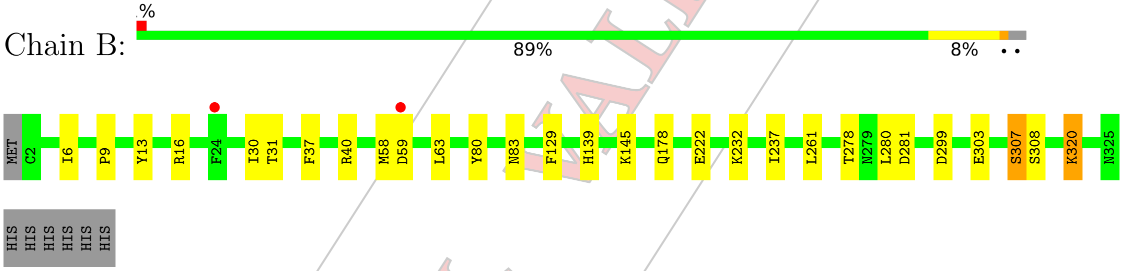

- Molecule 1: Choloylglycine hydrolase

Chain C:

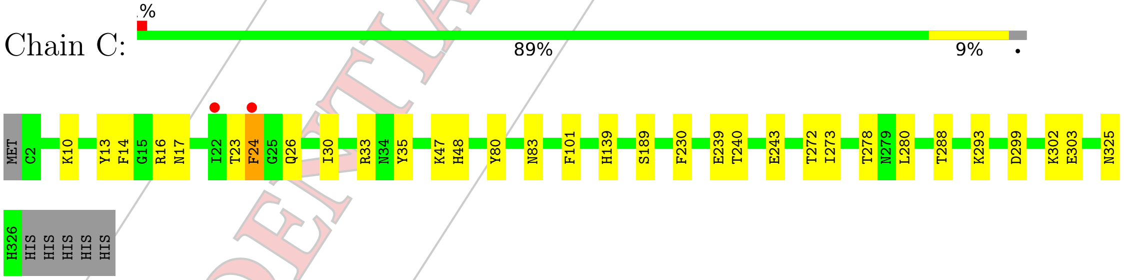

- Molecule 1: Choloylglycine hydrolase

Chain D:

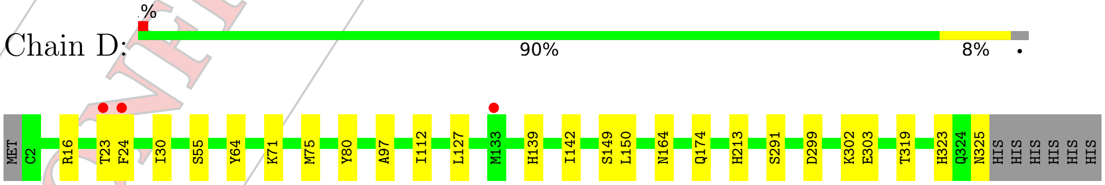

CONFIDENTIAL VALIDATION REPORT

4 Data and refinement statistics (i)

Property	Value	Source
Space group	P 1 21 1	Depositor
Cell constants<br>a, b, c, α, β, γ	84.56Å 92.95Å 87.06Å<br>90.00° 107.22° 90.00°	Depositor
Resolution (Å)	46.48 – 2.15<br>46.48 – 2.15	Depositor<br>EDS
% Data completeness<br>(in resolution range)	98.2 (46.48-2.15)<br>98.2 (46.48-2.15)	Depositor<br>EDS
R_{merge}	0.06	Depositor
R_{sym}	(Not available)	Depositor
$< I/\sigma(I) >$ [1]	2.79 (at 2.16Å)	Xtriage
Refinement program	PHENIX 1.17.1_3660, PHENIX 1.17.1_3660	Depositor
R, R_{free}	0.151 , 0.205<br>0.151 , 0.205	Depositor<br>DCC
R_{free} test set	2002 reflections (2.91%)	wwPDB-VP
Wilson B-factor (Å^2)	29.6	Xtriage
Anisotropy	0.432	Xtriage
Bulk solvent k_{sol}(e/Å^3), B_{sol}(Å^2)	0.36 , 43.5	EDS
L-test for twinning[2]	$< \|L\| > = 0.50$, $< L^2 > = 0.33$	Xtriage
Estimated twinning fraction	0.010 for l,-k,h	Xtriage
F_o,F_c correlation	0.97	EDS
Total number of atoms	11181	wwPDB-VP
Average B, all atoms (Å^2)	31.0	wwPDB-VP

Xtriage's analysis on translational NCS is as follows: *The largest off-origin peak in the Patterson function is 5.81% of the height of the origin peak. No significant pseudotranslation is detected.*

[1] Intensities estimated from amplitudes.

[2] Theoretical values of $< |L| >$, $< L^2 >$ for acentric reflections are 0.5, 0.333 respectively for untwinned datasets, and 0.375, 0.2 for perfectly twinned datasets.

CONFIDENTIAL VALIDATION REPORT

5 Model quality (i)

5.1 Standard geometry (i)

The Z score for a bond length (or angle) is the number of standard deviations the observed value is removed from the expected value. A bond length (or angle) with $|Z| > 5$ is considered an outlier worth inspection. RMSZ is the root-mean-square of all Z scores of the bond lengths (or angles).

Mol	Chain	Bond lengths		Bond angles	
		RMSZ	#$\|Z\|$ >5	RMSZ	#$\|Z\|$ >5
1	A	0.41	0/2661	0.57	0/3603
1	B	0.40	0/2648	0.58	0/3587
1	C	0.42	0/2677	0.57	0/3627
1	D	0.39	0/2653	0.56	0/3596
All	All	0.40	0/10639	0.57	0/14413

There are no bond length outliers.

There are no bond angle outliers.

There are no chirality outliers.

There are no planarity outliers.

5.2 Too-close contacts (i)

In the following table, the Non-H and H(model) columns list the number of non-hydrogen atoms and hydrogen atoms in the chain respectively. The H(added) column lists the number of hydrogen atoms added and optimized by MolProbity. The Clashes column lists the number of clashes within the asymmetric unit, whereas Symm-Clashes lists symmetry-related clashes.

Mol	Chain	Non-H	H(model)	H(added)	Clashes	Symm-Clashes
1	A	2596	0	2477	18	0
1	B	2584	0	2458	16	0
1	C	2611	0	2476	20	0
1	D	2588	0	2452	14	0
2	A	207	0	0	1	0
2	B	177	0	0	2	0
2	C	211	0	0	1	0
2	D	207	0	0	1	0
All	All	11181	0	9863	63	0

The all-atom clashscore is defined as the number of clashes found per 1000 atoms (including hydrogen atoms). The all-atom clashscore for this structure is 3.

CONFIDENTIAL VALIDATION REPORT

All (63) close contacts within the same asymmetric unit are listed below, sorted by their clash magnitude.

Atom-1	Atom-2	Interatomic distance (Å)	Clash overlap (Å)
1:B:58:MET:HG3	1:B:63:LEU:HD13	1.53	0.90
1:A:23:THR:HG22	1:A:25:GLY:H	1.42	0.84
1:C:47:LYS:NZ	2:C:401:HOH:O	2.15	0.79
1:C:23:THR:HB	1:C:26:GLN:HE22	1.49	0.77
1:C:10:LYS:HE2	1:C:239:GLU:HA	1.68	0.74
1:A:30:ILE:HD13	1:A:303:GLU:HG3	1.70	0.74
1:C:23:THR:HG22	1:C:273:ILE:HD12	1.71	0.71
1:A:174[B]:GLN:H	1:A:174[B]:GLN:CD	1.93	0.70
1:D:174[B]:GLN:H	1:D:174[B]:GLN:CD	2.00	0.64
1:C:33:ARG:HB3	1:C:47:LYS:HE3	1.81	0.63
1:B:40:ARG:NH1	2:B:402:HOH:O	2.33	0.60
1:C:299:ASP:HB3	1:C:302:LYS:HG3	1.83	0.60
1:A:84:ALA:HB3	1:A:137:SER:HB3	1.82	0.59
1:A:196:ASN:HB3	1:C:189:SER:HB2	1.84	0.59
1:B:6:ILE:O	1:B:232:LYS:HE2	2.03	0.58
1:B:320:LYS:NZ	2:B:403:HOH:O	2.38	0.55
1:A:317:ASP:OD1	1:A:318:LYS:HE2	2.06	0.55
1:D:23:THR:HG21	2:D:555:HOH:O	2.08	0.53
1:A:196:ASN:CB	1:C:189:SER:HB2	2.39	0.53
1:D:149:SER:OG	1:D:164:ASN:HB3	2.11	0.50
1:B:145:LYS:HG2	1:B:280:LEU:HB3	1.93	0.49
1:D:97:ALA:HB2	1:D:127:LEU:HB2	1.93	0.49
1:D:291:SER:O	1:D:319:THR:HG22	2.13	0.49
1:A:178:GLN:OE1	1:A:222:GLU:HG2	2.12	0.49
1:C:80:TYR:CE2	1:C:83:ASN:HB3	2.47	0.49
1:C:240:THR:HG22	1:C:243:GLU:H	1.78	0.49
1:A:257:GLN:NE2	1:A:262:ASP:OD2	2.41	0.48
1:B:299:ASP:O	1:C:325:ASN:ND2	2.47	0.48
1:C:23:THR:OG1	1:C:24:PHE:N	2.47	0.47
1:A:258:GLN:HB2	1:A:261:LEU:HD22	1.96	0.46
1:C:23:THR:CG2	1:C:273:ILE:HD12	2.42	0.46
1:D:55:SER:HB2	1:D:64:TYR:CD1	2.51	0.45
1:A:117:ASP:O	1:A:120:SER:OG	2.33	0.45
1:D:30:ILE:HD12	1:D:303:GLU:HG3	1.99	0.45
1:D:291:SER:HB3	1:D:319:THR:HG23	1.98	0.45
1:A:156:LYS:HG3	2:A:533:HOH:O	2.16	0.44
1:C:14:PHE:HB3	1:C:280:LEU:HD21	1.98	0.44
1:A:299:ASP:HB3	1:A:302:LYS:HG3	1.98	0.44
1:D:112:ILE:HG23	1:D:150:LEU:HD23	1.99	0.44
1:A:68:THR:HG21	1:A:278:THR:HG21	2.00	0.44

Continued on next page...

CONFIDENTIAL VALIDATION REPORT

Continued from previous page...

Atom-1	Atom-2	Interatomic distance (Å)	Clash overlap (Å)
1:B:40:ARG:NH2	1:B:129:PHE:O	2.51	0.44
1:C:288:THR:HB	1:C:293:LYS:HA	1.99	0.44
1:A:6:ILE:HD12	1:A:143:ALA:HB1	1.98	0.43
1:A:220:ASP:OD1	1:D:213:HIS:HD2	2.00	0.43
1:B:261:LEU:HG	1:C:230:PHE:CE2	2.54	0.43
1:B:307:SER:OG	1:B:308:SER:N	2.52	0.43
1:B:31:THR:HG21	1:B:37:PHE:CZ	2.54	0.42
1:D:299:ASP:HB3	1:D:302:LYS:HD2	2.01	0.42
1:B:13:TYR:HA	1:B:278:THR:O	2.19	0.42
1:B:80:TYR:CE2	1:B:83:ASN:HB3	2.54	0.42
1:B:145:LYS:HD2	1:B:281:ASP:HA	2.01	0.42
1:B:30:ILE:HD13	1:B:303:GLU:HG3	2.02	0.42
1:B:178:GLN:OE1	1:B:222:GLU:HG2	2.20	0.42
1:B:9:PRO:HA	1:B:237:ILE:HD13	2.01	0.42
1:C:13:TYR:HA	1:C:278:THR:O	2.20	0.41
1:D:80:TYR:OH	1:D:174[A]:GLN:HG2	2.19	0.41
1:A:9:PRO:HA	1:A:237:ILE:HD12	2.02	0.41
1:A:75:MET:HG3	1:A:142:ILE:HG12	2.02	0.41
1:D:75:MET:HG3	1:D:142:ILE:HG12	2.03	0.41
1:C:17:ASN:HB3	1:C:272:THR:HG23	2.03	0.41
1:C:35:TYR:O	1:C:48:HIS:NE2	2.46	0.40
1:C:30:ILE:CD1	1:C:303:GLU:HG3	2.51	0.40
1:D:71:LYS:HA	1:D:71:LYS:HD2	1.95	0.40

There are no symmetry-related clashes.

5.3 Torsion angles (i)

5.3.1 Protein backbone (i)

In the following table, the Percentiles column shows the percent Ramachandran outliers of the chain as a percentile score with respect to all X-ray entries followed by that with respect to entries of similar resolution.

The Analysed column shows the number of residues for which the backbone conformation was analysed, and the total number of residues.

Mol	Chain	Analysed	Favoured	Allowed	Outliers	Percentiles	
1	A	323/331 (98%)	313 (97%)	10 (3%)	0	100	100
1	B	323/331 (98%)	314 (97%)	8 (2%)	1 (0%)	41	37

Continued on next page...

CONFIDENTIAL VALIDATION REPORT

Continued from previous page...

Mol	Chain	Analysed	Favoured	Allowed	Outliers	Percentiles	
1	C	325/331 (98%)	315 (97%)	9 (3%)	1 (0%)	41	37
1	D	323/331 (98%)	314 (97%)	9 (3%)	0	100	100
All	All	1294/1324 (98%)	1256 (97%)	36 (3%)	2 (0%)	47	46

All (2) Ramachandran outliers are listed below:

Mol	Chain	Res	Type
1	B	59	ASP
1	C	24	PHE

5.3.2 Protein sidechains ⓘ

In the following table, the Percentiles column shows the percent sidechain outliers of the chain as a percentile score with respect to all X-ray entries followed by that with respect to entries of similar resolution.

The Analysed column shows the number of residues for which the sidechain conformation was analysed, and the total number of residues.

Mol	Chain	Analysed	Rotameric	Outliers	Percentiles	
1	A	289/298 (97%)	282 (98%)	7 (2%)	49	51
1	B	286/298 (96%)	282 (99%)	4 (1%)	67	72
1	C	290/298 (97%)	287 (99%)	3 (1%)	76	81
1	D	287/298 (96%)	282 (98%)	5 (2%)	60	65
All	All	1152/1192 (97%)	1133 (98%)	19 (2%)	60	67

All (19) residues with a non-rotameric sidechain are listed below:

Mol	Chain	Res	Type
1	A	16	ARG
1	A	101	PHE
1	A	139	HIS
1	A	182	LEU
1	A	202	ASN
1	A	315	MET
1	A	318	LYS
1	B	16	ARG
1	B	139	HIS
1	B	307	SER

Continued on next page...

CONFIDENTIAL VALIDATION REPORT

Continued from previous page...

Mol	Chain	Res	Type
1	B	320	LYS
1	C	16	ARG
1	C	101	PHE
1	C	139	HIS
1	D	16	ARG
1	D	24	PHE
1	D	139	HIS
1	D	323	HIS
1	D	325	ASN

Sometimes sidechains can be flipped to improve hydrogen bonding and reduce clashes. All (3) such sidechains are listed below:

Mol	Chain	Res	Type
1	B	79	ASN
1	C	26	GLN
1	D	213	HIS

5.3.3 RNA ⓘ

There are no RNA molecules in this entry.

5.4 Non-standard residues in protein, DNA, RNA chains ⓘ

There are no non-standard protein/DNA/RNA residues in this entry.

5.5 Carbohydrates ⓘ

There are no monosaccharides in this entry.

5.6 Ligand geometry ⓘ

There are no ligands in this entry.

5.7 Other polymers ⓘ

There are no such residues in this entry.

CONFIDENTIAL VALIDATION REPORT

5.8 Polymer linkage issues (i)

There are no chain breaks in this entry.

CONFIDENTIAL VALIDATION REPORT

6 Fit of model and data (i)

6.1 Protein, DNA and RNA chains (i)

In the following table, the column labelled '#RSRZ> 2' contains the number (and percentage) of RSRZ outliers, followed by percent RSRZ outliers for the chain as percentile scores relative to all X-ray entries and entries of similar resolution. The OWAB column contains the minimum, median, 95th percentile and maximum values of the occupancy-weighted average B-factor per residue. The column labelled 'Q< 0.9' lists the number of (and percentage) of residues with an average occupancy less than 0.9.

Mol	Chain	Analysed	<RSRZ>	#RSRZ>2			OWAB(Å^2)	Q<0.9
1	A	324/331 (97%)	-0.40	0	100	100	20, 29, 44, 56	0
1	B	324/331 (97%)	-0.23	2 (0%)	89	91	21, 32, 49, 72	0
1	C	325/331 (98%)	-0.29	2 (0%)	89	91	21, 30, 44, 59	0
1	D	324/331 (97%)	-0.40	3 (0%)	84	88	21, 29, 44, 60	0
All	All	1297/1324 (97%)	-0.33	7 (0%)	91	93	20, 30, 45, 72	0

All (7) RSRZ outliers are listed below:

Mol	Chain	Res	Type	RSRZ
1	D	23	THR	3.9
1	C	22	ILE	3.4
1	C	24	PHE	2.7
1	B	59	ASP	2.5
1	B	24	PHE	2.4
1	D	133	MET	2.4
1	D	24	PHE	2.2

6.2 Non-standard residues in protein, DNA, RNA chains (i)

There are no non-standard protein/DNA/RNA residues in this entry.

6.3 Carbohydrates (i)

There are no monosaccharides in this entry.

6.4 Ligands (i)

There are no ligands in this entry.

CONFIDENTIAL VALIDATION REPORT

6.5 Other polymers (i)

There are no such residues in this entry.

CONFIDENTIAL VALIDATION REPORT
