# Supplementary material for: Bile salt hydrolases shape the bile acid landscape and restrict Clostridioides difficile growth in the murine gut
Source: Nat Microbiol. 2023 Mar 13;8(4):611–28. doi: 10.1038/s41564-023-01337-7 (PMC10066039; doi:10.1038/s41564-023-01337-7)
Supplement: Supplementary file 8 — 7SVG PDB validation report. [file 41564_2023_1337_MOESM8_ESM.pdf]

Full wwPDB X-ray Structure Validation Report (i)

Nov 22, 2021 – 11:31 AM EST

PDB ID	:	7SVG
Title	:	Bile Salt Hydrolase A from Lactobacillus gasseri with chenodeoxycholate and taurine bound
Deposited on	:	2021-11-19
Resolution	:	1.35 Å(reported)

This is a Full wwPDB X-ray Structure Validation Report.

This report is produced by the wwPDB biocuration pipeline after annotation of the structure.

We welcome your comments at *validation@mail.wwpdb.org*
A user guide is available at
https://www.wwpdb.org/validation/2017/XrayValidationReportHelp
with specific help available everywhere you see the (i) symbol.

CONFIDENTIAL VALIDATION REPORT

The following versions of software and data (see references (i)) were used in the production of this report:

MolProbity	:	4.02b-467
Mogul	:	1.8.5 (274361), CSD as541be (2020)
Xtriage (Phenix)	:	1.13
EDS	:	2.23.2
buster-report	:	1.1.7 (2018)
Percentile statistics	:	20191225.v01 (using entries in the PDB archive December 25th 2019)
Refmac	:	5.8.0158
CCP4	:	7.0.044 (Gargrove)
Ideal geometry (proteins)	:	Engh & Huber (2001)
Ideal geometry (DNA, RNA)	:	Parkinson et al. (1996)
Validation Pipeline (wwPDB-VP)	:	2.23.2

1 Overall quality at a glance (i)

The following experimental techniques were used to determine the structure:
X-RAY DIFFRACTION

The reported resolution of this entry is 1.35 Å.

Percentile scores (ranging between 0-100) for global validation metrics of the entry are shown in the following graphic. The table shows the number of entries on which the scores are based.

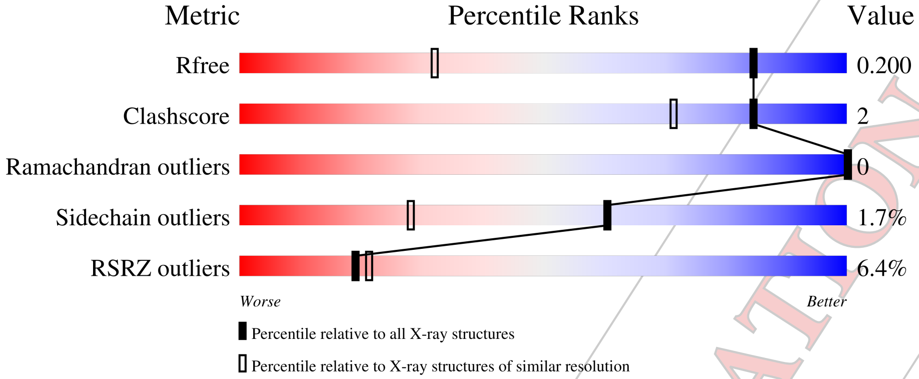

Metric	Whole archive (#Entries)	Similar resolution (#Entries, resolution range(Å))
R_{free}	130704	1509 (1.38-1.34)
Clashscore	141614	1551 (1.38-1.34)
Ramachandran outliers	138981	1530 (1.38-1.34)
Sidechain outliers	138945	1530 (1.38-1.34)
RSRZ outliers	127900	1487 (1.38-1.34)

The table below summarises the geometric issues observed across the polymeric chains and their fit to the electron density. The red, orange, yellow and green segments of the lower bar indicate the fraction of residues that contain outliers for >=3, 2, 1 and 0 types of geometric quality criteria respectively. A grey segment represents the fraction of residues that are not modelled. The numeric value for each fraction is indicated below the corresponding segment, with a dot representing fractions <=5% The upper red bar (where present) indicates the fraction of residues that have poor fit to the electron density. The numeric value is given above the bar.

Mol	Chain	Length	Quality of chain
1	A	322	6% / 94% • •
1	B	322	9% / 91% 6% • •
1	C	322	7% / 93% • • •
1	D	322	4% / 92% 5% • •

The following table lists non-polymeric compounds, carbohydrate monomers and non-standard

CONFIDENTIAL VALIDATION REPORT

residues in protein, DNA, RNA chains that are outliers for geometric or electron-density-fit criteria:

Mol	Type	Chain	Res	Chirality	Geometry	Clashes	Electron density
2	TAU	A	501	-	X	-	-

CONFIDENTIAL VALIDATION REPORT

2 Entry composition (i)

There are 4 unique types of molecules in this entry. The entry contains 11398 atoms, of which 117 are hydrogens and 0 are deuteriums.

In the tables below, the ZeroOcc column contains the number of atoms modelled with zero occupancy, the AltConf column contains the number of residues with at least one atom in alternate conformation and the Trace column contains the number of residues modelled with at most 2 atoms.

- Molecule 1 is a protein called Choloylglycine hydrolase.

Mol	Chain	Residues	Atoms					ZeroOcc	AltConf	Trace
			Total	C	N	O	S			
1	A	316	2459	1577	394	479	9	0	0	0
1	B	315	2453	1574	392	478	9	0	0	0
1	C	315	2434	1562	389	474	9	0	0	0
1	D	315	2437	1564	389	475	9	0	0	0

There are 24 discrepancies between the modelled and reference sequences:

Chain	Residue	Modelled	Actual	Comment	Reference
A	317	HIS	-	expression tag	UNP A0A8A6B131
A	318	HIS	-	expression tag	UNP A0A8A6B131
A	319	HIS	-	expression tag	UNP A0A8A6B131
A	320	HIS	-	expression tag	UNP A0A8A6B131
A	321	HIS	-	expression tag	UNP A0A8A6B131
A	322	HIS	-	expression tag	UNP A0A8A6B131
B	317	HIS	-	expression tag	UNP A0A8A6B131
B	318	HIS	-	expression tag	UNP A0A8A6B131
B	319	HIS	-	expression tag	UNP A0A8A6B131
B	320	HIS	-	expression tag	UNP A0A8A6B131
B	321	HIS	-	expression tag	UNP A0A8A6B131
B	322	HIS	-	expression tag	UNP A0A8A6B131
C	317	HIS	-	expression tag	UNP A0A8A6B131
C	318	HIS	-	expression tag	UNP A0A8A6B131
C	319	HIS	-	expression tag	UNP A0A8A6B131
C	320	HIS	-	expression tag	UNP A0A8A6B131
C	321	HIS	-	expression tag	UNP A0A8A6B131
C	322	HIS	-	expression tag	UNP A0A8A6B131
D	317	HIS	-	expression tag	UNP A0A8A6B131
D	318	HIS	-	expression tag	UNP A0A8A6B131
D	319	HIS	-	expression tag	UNP A0A8A6B131

Continued on next page...

CONFIDENTIAL VALIDATION REPORT

Continued from previous page...

Chain	Residue	Modelled	Actual	Comment	Reference
D	320	HIS	-	expression tag	UNP A0A8A6B131
D	321	HIS	-	expression tag	UNP A0A8A6B131
D	322	HIS	-	expression tag	UNP A0A8A6B131

- Molecule 2 is 2-AMINOETHANESULFONIC ACID (three-letter code: TAU) (formula: $C_2H_7NO_3S$) (labeled as "Ligand of Interest" by depositor).

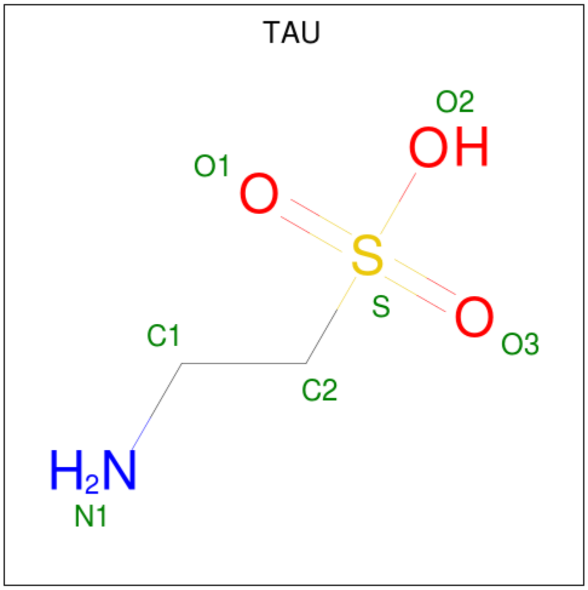

Mol	Chain	Residues	Atoms	ZeroOcc	AltConf
2	A	1	Total C N O S<br>7 2 1 3 1	0	0
2	B	1	Total C N O S<br>7 2 1 3 1	0	0
2	C	1	Total C N O S<br>7 2 1 3 1	0	0
2	D	1	Total C N O S<br>7 2 1 3 1	0	0

- Molecule 3 is CHENODEOXYCHOLIC ACID (three-letter code: JN3) (formula: $C_{24}H_{40}O_4$) (labeled as "Ligand of Interest" by depositor).

CONFIDENTIAL VALIDATION REPORT

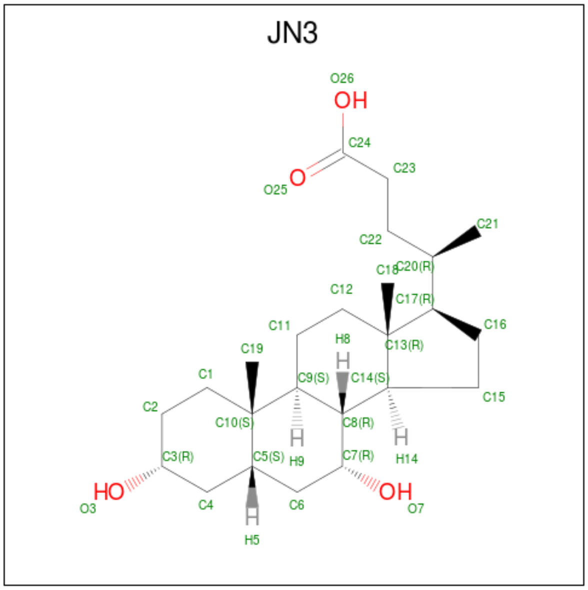

Mol	Chain	Residues	Atoms	ZeroOcc	AltConf
3	A	1	Total C H O<br>67 24 39 4	0	0
3	A	1	Total C H O<br>67 24 39 4	0	0
3	D	1	Total C H O<br>67 24 39 4	0	0

- Molecule 4 is water.

Mol	Chain	Residues	Atoms	ZeroOcc	AltConf
4	A	359	Total O<br>359 359	0	0
4	B	311	Total O<br>311 311	0	0
4	C	351	Total O<br>351 351	0	0
4	D	365	Total O<br>365 365	0	0

CONFIDENTIAL VALIDATION REPORT

3 Residue-property plots (i)

These plots are drawn for all protein, RNA, DNA and oligosaccharide chains in the entry. The first graphic for a chain summarises the proportions of the various outlier classes displayed in the second graphic. The second graphic shows the sequence view annotated by issues in geometry and electron density. Residues are color-coded according to the number of geometric quality criteria for which they contain at least one outlier: green = 0, yellow = 1, orange = 2 and red = 3 or more. A red dot above a residue indicates a poor fit to the electron density (RSRZ > 2). Stretches of 2 or more consecutive residues without any outlier are shown as a green connector. Residues present in the sample, but not in the model, are shown in grey.

• Molecule 1: Choloylglycine hydrolase

Chain A:

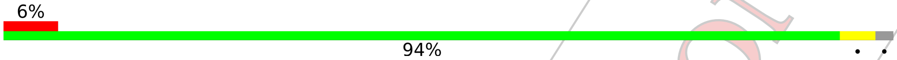

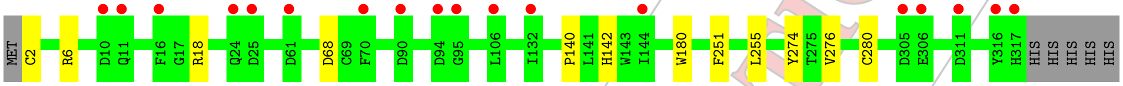

• Molecule 1: Choloylglycine hydrolase

Chain B:

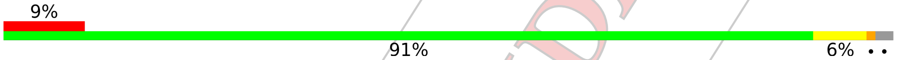

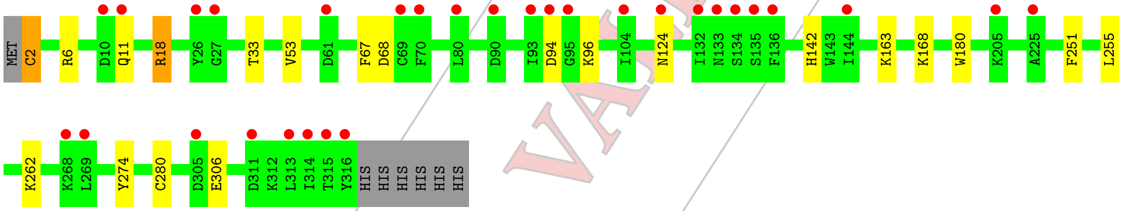

• Molecule 1: Choloylglycine hydrolase

Chain C:

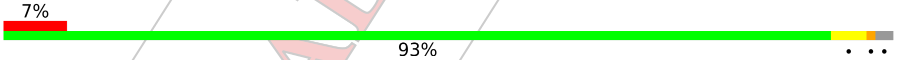

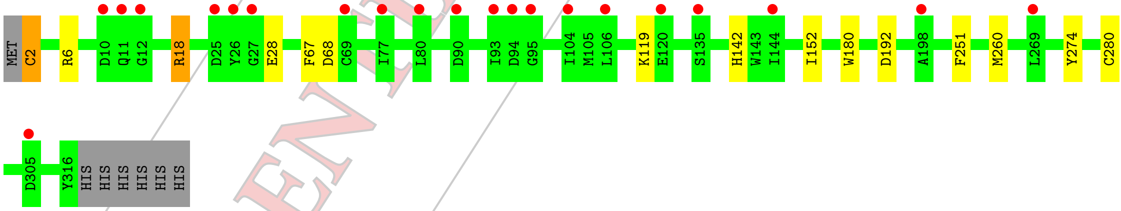

• Molecule 1: Choloylglycine hydrolase

Chain D:

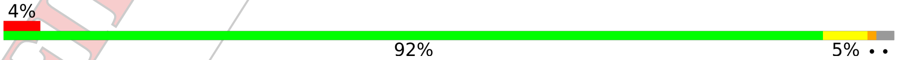

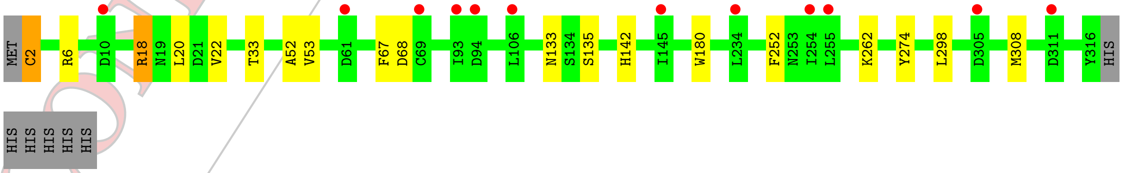

CONFIDENTIAL VALIDATION REPORT

4 Data and refinement statistics (i)

Property	Value	Source
Space group	P 1 21 1	Depositor
Cell constants<br>a, b, c, α, β, γ	59.15Å 153.12Å 94.98Å<br>90.00° 105.80° 90.00°	Depositor
Resolution (Å)	29.34 – 1.35<br>29.34 – 1.35	Depositor<br>EDS
% Data completeness<br>(in resolution range)	92.9 (29.34-1.35)<br>92.9 (29.34-1.35)	Depositor<br>EDS
R_{merge}	0.10	Depositor
R_{sym}	(Not available)	Depositor
$< I/\sigma(I) >$ [1]	0.99 (at 1.35Å)	Xtriage
Refinement program	PHENIX 1.19.2_4158, PHENIX 1.19.2_4158	Depositor
R, R_{free}	0.178 , 0.200<br>0.178 , 0.200	Depositor<br>DCC
R_{free} test set	1965 reflections (0.60%)	wwPDB-VP
Wilson B-factor (Å^2)	20.1	Xtriage
Anisotropy	0.215	Xtriage
Bulk solvent k_{sol}(e/Å^3), B_{sol}(Å^2)	0.35 , 42.5	EDS
L-test for twinning[2]	$< \|L\| > = 0.50$, $< L^2 > = 0.33$	Xtriage
Estimated twinning fraction	0.018 for h,-k,-h-l	Xtriage
F_o,F_c correlation	0.98	EDS
Total number of atoms	11398	wwPDB-VP
Average B, all atoms (Å^2)	25.0	wwPDB-VP

Xtriage's analysis on translational NCS is as follows: *The largest off-origin peak in the Patterson function is 4.16% of the height of the origin peak. No significant pseudotranslation is detected.*

[1] Intensities estimated from amplitudes.

[2] Theoretical values of $< |L| >$, $< L^2 >$ for acentric reflections are 0.5, 0.333 respectively for untwinned datasets, and 0.375, 0.2 for perfectly twinned datasets.

CONFIDENTIAL VALIDATION REPORT

5 Model quality (i)

5.1 Standard geometry (i)

Bond lengths and bond angles in the following residue types are not validated in this section: OCS, TAU, JN3

The Z score for a bond length (or angle) is the number of standard deviations the observed value is removed from the expected value. A bond length (or angle) with $|Z| > 5$ is considered an outlier worth inspection. RMSZ is the root-mean-square of all Z scores of the bond lengths (or angles).

Mol	Chain	Bond lengths		Bond angles	
		RMSZ	$\#\|Z\| > 5$	RMSZ	$\#\|Z\| > 5$
1	A	0.35	0/2512	0.60	0/3422
1	B	0.35	0/2505	0.57	0/3411
1	C	0.36	0/2486	0.58	0/3390
1	D	0.36	0/2489	0.60	0/3393
All	All	0.36	0/9992	0.59	0/13616

There are no bond length outliers.

There are no bond angle outliers.

There are no chirality outliers.

There are no planarity outliers.

5.2 Too-close contacts (i)

In the following table, the Non-H and H(model) columns list the number of non-hydrogen atoms and hydrogen atoms in the chain respectively. The H(added) column lists the number of hydrogen atoms added and optimized by MolProbity. The Clashes column lists the number of clashes within the asymmetric unit, whereas Symm-Clashes lists symmetry-related clashes.

Mol	Chain	Non-H	H(model)	H(added)	Clashes	Symm-Clashes
1	A	2459	0	2351	5	0
1	B	2453	0	2355	13	0
1	C	2434	0	2316	10	0
1	D	2437	0	2323	13	0
2	A	7	0	7	1	0
2	B	7	0	7	1	0
2	C	7	0	7	2	0
2	D	7	0	7	1	0
3	A	56	78	78	4	0

Continued on next page...

CONFIDENTIAL VALIDATION REPORT

Continued from previous page...

Mol	Chain	Non-H	H(model)	H(added)	Clashes	Symm-Clashes
3	D	28	39	39	6	0
4	A	359	0	0	1	0
4	B	311	0	0	4	0
4	C	351	0	0	1	0
4	D	365	0	0	0	0
All	All	11281	117	9490	45	0

The all-atom clashscore is defined as the number of clashes found per 1000 atoms (including hydrogen atoms). The all-atom clashscore for this structure is 2.

All (45) close contacts within the same asymmetric unit are listed below, sorted by their clash magnitude.

Atom-1	Atom-2	Interatomic distance (Å)	Clash overlap (Å)
1:D:20:LEU:HD11	3:D:402:JN3:H212	1.64	0.79
1:B:2:OCS:H	2:B:401:TAU:H1N2	1.48	0.62
3:D:402:JN3:H183	3:D:402:JN3:H213	1.83	0.60
1:B:96:LYS:HD3	1:B:124:ASN:ND2	2.16	0.60
1:C:28:GLU:CD	1:C:28:GLU:H	2.06	0.59
3:A:503:JN3:H121	3:A:503:JN3:H212	1.85	0.58
3:A:502:JN3:H183	3:A:502:JN3:H212	1.88	0.54
1:D:22:VAL:HG12	3:D:402:JN3:H231	1.91	0.52
1:C:119:LYS:HE2	1:C:152:ILE:HG21	1.90	0.52
1:D:2:OCS:OD3	1:D:18:ARG:NH2	2.44	0.49
3:D:402:JN3:H213	3:D:402:JN3:C18	2.42	0.48
1:D:262:LYS:HE2	1:D:274:TYR:CE1	2.47	0.48
1:C:192:ASP:OD1	4:C:501:HOH:O	2.20	0.47
1:C:2:OCS:OD3	1:C:18:ARG:NH2	2.46	0.47
1:C:260:MET:HB3	1:C:274:TYR:HA	1.97	0.47
1:C:251:PHE:CD1	1:C:280:CYS:HB2	2.49	0.47
1:A:274:TYR:CE2	1:A:276:VAL:HG12	2.50	0.47
1:D:252:PHE:CZ	1:D:298:LEU:HD21	2.50	0.47
1:B:180:TRP:CD1	1:C:180:TRP:CD1	3.03	0.47
1:B:168:LYS:NZ	4:B:509:HOH:O	2.48	0.46
1:D:20:LEU:CD1	3:D:402:JN3:H212	2.38	0.46
1:C:2:OCS:OD2	2:C:401:TAU:N1	2.49	0.46
1:A:180:TRP:CD1	1:D:180:TRP:CD1	3.04	0.46
1:D:2:OCS:OD2	2:D:401:TAU:N1	2.48	0.46
1:D:52:ALA:CB	1:D:308:MET:HG2	2.46	0.46
1:B:262:LYS:HE3	4:B:672:HOH:O	2.17	0.45
1:D:67:PHE:CD1	1:D:67:PHE:N	2.83	0.45
1:B:262:LYS:HE2	1:B:274:TYR:CE1	2.51	0.45

Continued on next page...

CONFIDENTIAL VALIDATION REPORT

Continued from previous page...

Atom-1	Atom-2	Interatomic distance (Å)	Clash overlap (Å)
1:C:2:OCS:H	2:C:401:TAU:H1N2	1.65	0.44
1:B:2:OCS:OD2	1:B:18:ARG:NH2	2.50	0.44
1:D:33:THR:HB	1:D:53:VAL:HG22	2.00	0.43
1:C:67:PHE:CD1	1:C:67:PHE:N	2.85	0.43
1:D:133:ASN:OD1	1:D:135:SER:HB2	2.19	0.43
1:B:255:LEU:HD23	1:B:255:LEU:HA	1.89	0.43
2:A:501:TAU:H1C1	4:A:912:HOH:O	2.18	0.42
1:A:140:PRO:HD3	3:A:502:JN3:H222	2.02	0.41
1:B:33:THR:HB	1:B:53:VAL:HG22	2.02	0.41
1:D:20:LEU:HD11	3:D:402:JN3:C21	2.43	0.41
3:A:502:JN3:H112	3:A:502:JN3:H12	1.88	0.41
1:B:251:PHE:CD1	1:B:280:CYS:HB2	2.56	0.41
1:B:67:PHE:N	1:B:67:PHE:CD1	2.89	0.40
1:A:251:PHE:CD1	1:A:280:CYS:HB2	2.56	0.40
1:B:306:GLU:OE1	4:B:501:HOH:O	2.22	0.40
1:A:255:LEU:HD23	1:A:255:LEU:HA	1.84	0.40
1:B:163:LYS:NZ	4:B:506:HOH:O	2.44	0.40

There are no symmetry-related clashes.

5.3 Torsion angles (i)

5.3.1 Protein backbone (i)

In the following table, the Percentiles column shows the percent Ramachandran outliers of the chain as a percentile score with respect to all X-ray entries followed by that with respect to entries of similar resolution.

The Analysed column shows the number of residues for which the backbone conformation was analysed, and the total number of residues.

Mol	Chain	Analysed	Favoured	Allowed	Outliers	Percentiles	
1	A	314/322 (98%)	304 (97%)	10 (3%)	0	100	100
1	B	313/322 (97%)	303 (97%)	10 (3%)	0	100	100
1	C	313/322 (97%)	303 (97%)	10 (3%)	0	100	100
1	D	313/322 (97%)	305 (97%)	8 (3%)	0	100	100
All	All	1253/1288 (97%)	1215 (97%)	38 (3%)	0	100	100

There are no Ramachandran outliers to report.

CONFIDENTIAL VALIDATION REPORT

5.3.2 Protein sidechains (i)

In the following table, the Percentiles column shows the percent sidechain outliers of the chain as a percentile score with respect to all X-ray entries followed by that with respect to entries of similar resolution.

The Analysed column shows the number of residues for which the sidechain conformation was analysed, and the total number of residues.

Mol	Chain	Analysed	Rotameric	Outliers	Percentiles	
1	A	262/272 (96%)	258 (98%)	4 (2%)	65	33
1	B	262/272 (96%)	256 (98%)	6 (2%)	50	16
1	C	257/272 (94%)	253 (98%)	4 (2%)	62	30
1	D	258/272 (95%)	254 (98%)	4 (2%)	62	30
All	All	1039/1088 (96%)	1021 (98%)	18 (2%)	60	28

All (18) residues with a non-rotameric sidechain are listed below:

Mol	Chain	Res	Type
1	A	6	ARG
1	A	18	ARG
1	A	68	ASP
1	A	142	HIS
1	B	6	ARG
1	B	11	GLN
1	B	18	ARG
1	B	68	ASP
1	B	94	ASP
1	B	142	HIS
1	C	6	ARG
1	C	18	ARG
1	C	68	ASP
1	C	142	HIS
1	D	6	ARG
1	D	18	ARG
1	D	68	ASP
1	D	142	HIS

Sometimes sidechains can be flipped to improve hydrogen bonding and reduce clashes. All (3) such sidechains are listed below:

Mol	Chain	Res	Type
1	B	124	ASN

Continued on next page...

CONFIDENTIAL VALIDATION REPORT

Continued from previous page...

Mol	Chain	Res	Type
1	B	202	ASN
1	C	202	ASN

5.3.3 RNA (i)

There are no RNA molecules in this entry.

5.4 Non-standard residues in protein, DNA, RNA chains (i)

4 non-standard protein/DNA/RNA residues are modelled in this entry.

In the following table, the Counts columns list the number of bonds (or angles) for which Mogul statistics could be retrieved, the number of bonds (or angles) that are observed in the model and the number of bonds (or angles) that are defined in the Chemical Component Dictionary. The Link column lists molecule types, if any, to which the group is linked. The Z score for a bond length (or angle) is the number of standard deviations the observed value is removed from the expected value. A bond length (or angle) with $|Z| > 2$ is considered an outlier worth inspection. RMSZ is the root-mean-square of all Z scores of the bond lengths (or angles).

Mol	Type	Chain	Res	Link	Bond lengths			Bond angles		
					Counts	RMSZ	$\#\|Z\| > 2$	Counts	RMSZ	$\#\|Z\| > 2$
1	OCS	D	2	1	7,8,9	0.92	0	6,11,13	1.87	1 (16%)
1	OCS	C	2	1	7,8,9	0.87	0	6,11,13	1.63	1 (16%)
1	OCS	A	2	1	7,8,9	0.86	0	6,11,13	1.83	1 (16%)
1	OCS	B	2	1	7,8,9	0.85	0	6,11,13	1.72	1 (16%)

In the following table, the Chirals column lists the number of chiral outliers, the number of chiral centers analysed, the number of these observed in the model and the number defined in the Chemical Component Dictionary. Similar counts are reported in the Torsion and Rings columns. '-' means no outliers of that kind were identified.

Mol	Type	Chain	Res	Link	Chirals	Torsions	Rings
1	OCS	D	2	1	-	2/4/7/9	-
1	OCS	C	2	1	-	3/4/7/9	-
1	OCS	A	2	1	-	2/4/7/9	-
1	OCS	B	2	1	-	2/4/7/9	-

There are no bond length outliers.

All (4) bond angle outliers are listed below:

CONFIDENTIAL VALIDATION REPORT

Mol	Chain	Res	Type	Atoms	Z	Observed(°)	Ideal(°)
1	A	2	OCS	OD3-SG-CB	3.76	111.41	106.94
1	B	2	OCS	OD3-SG-CB	3.58	111.19	106.94
1	D	2	OCS	OD1-SG-CB	3.54	111.14	106.94
1	C	2	OCS	OD1-SG-CB	2.91	110.40	106.94

There are no chirality outliers.

All (9) torsion outliers are listed below:

Mol	Chain	Res	Type	Atoms
1	C	2	OCS	CA-CB-SG-OD2
1	D	2	OCS	CA-CB-SG-OD2
1	B	2	OCS	CA-CB-SG-OD1
1	B	2	OCS	CA-CB-SG-OD3
1	C	2	OCS	CA-CB-SG-OD1
1	C	2	OCS	CA-CB-SG-OD3
1	A	2	OCS	CA-CB-SG-OD3
1	D	2	OCS	CA-CB-SG-OD1
1	A	2	OCS	CA-CB-SG-OD1

There are no ring outliers.

3 monomers are involved in 7 short contacts:

Mol	Chain	Res	Type	Clashes	Symm-Clashes
1	D	2	OCS	2	0
1	C	2	OCS	3	0
1	B	2	OCS	2	0

5.5 Carbohydrates (i)

There are no monosaccharides in this entry.

5.6 Ligand geometry (i)

7 ligands are modelled in this entry.

In the following table, the Counts columns list the number of bonds (or angles) for which Mogul statistics could be retrieved, the number of bonds (or angles) that are observed in the model and the number of bonds (or angles) that are defined in the Chemical Component Dictionary. The Link column lists molecule types, if any, to which the group is linked. The Z score for a bond length (or angle) is the number of standard deviations the observed value is removed from the expected value. A bond length (or angle) with $|Z| > 2$ is considered an outlier worth inspection. RMSZ is the root-mean-square of all Z scores of the bond lengths (or angles).

CONFIDENTIAL VALIDATION REPORT

Mol	Type	Chain	Res	Link	Bond lengths			Bond angles		
					Counts	RMSZ	#|Z| > 2	Counts	RMSZ	#|Z| > 2
2	TAU	B	401	-	6,6,6	1.66	2 (33%)	6,8,8	2.96	4 (66%)
3	JN3	D	402	-	28,31,31	0.62	0	46,49,49	1.48	5 (10%)
2	TAU	C	401	-	6,6,6	1.77	3 (50%)	6,8,8	2.50	4 (66%)
3	JN3	A	503	-	28,31,31	0.59	0	46,49,49	1.11	3 (6%)
3	JN3	A	502	-	28,31,31	0.68	0	46,49,49	1.27	6 (13%)
2	TAU	D	401	-	6,6,6	1.82	2 (33%)	6,8,8	2.39	4 (66%)
2	TAU	A	501	-	6,6,6	1.74	2 (33%)	6,8,8	2.29	4 (66%)

In the following table, the Chirals column lists the number of chiral outliers, the number of chiral centers analysed, the number of these observed in the model and the number defined in the Chemical Component Dictionary. Similar counts are reported in the Torsion and Rings columns. '-' means no outliers of that kind were identified.

Mol	Type	Chain	Res	Link	Chirals	Torsions	Rings
2	TAU	B	401	-	-	0/4/4/4	-
3	JN3	D	402	-	-	6/7/70/70	0/4/4/4
2	TAU	C	401	-	-	0/4/4/4	-
3	JN3	A	503	-	-	0/7/70/70	0/4/4/4
3	JN3	A	502	-	-	6/7/70/70	0/4/4/4
2	TAU	D	401	-	-	1/4/4/4	-
2	TAU	A	501	-	-	4/4/4/4	-

All (9) bond length outliers are listed below:

Mol	Chain	Res	Type	Atoms	Z	Observed(Å)	Ideal(Å)
2	D	401	TAU	C2-S	3.24	1.82	1.77
2	A	501	TAU	C2-S	3.11	1.81	1.77
2	C	401	TAU	C2-S	3.01	1.81	1.77
2	B	401	TAU	C2-S	2.73	1.81	1.77
2	D	401	TAU	O3-S	2.30	1.51	1.45
2	B	401	TAU	O3-S	2.26	1.51	1.45
2	C	401	TAU	O1-S	2.18	1.51	1.45
2	C	401	TAU	O3-S	2.12	1.51	1.45
2	A	501	TAU	O1-S	2.09	1.51	1.45

All (30) bond angle outliers are listed below:

Mol	Chain	Res	Type	Atoms	Z	Observed(^{o})	Ideal(^{o})
3	D	402	JN3	C21-C20-C22	5.08	118.32	110.36
3	D	402	JN3	C13-C17-C20	4.04	125.82	119.49

Continued on next page...

CONFIDENTIAL VALIDATION REPORT

Continued from previous page...

Mol	Chain	Res	Type	Atoms	Z	Observed(^{o})	Ideal(^{o})
2	B	401	TAU	O1-S-C2	4.04	111.78	106.92
2	B	401	TAU	O3-S-C2	3.76	111.44	106.92
2	D	401	TAU	O2-S-O1	-3.75	102.12	111.27
2	B	401	TAU	O2-S-O1	-3.74	102.14	111.27
2	C	401	TAU	O2-S-O1	-3.36	103.06	111.27
2	C	401	TAU	O1-S-C2	3.24	110.82	106.92
2	C	401	TAU	O3-S-C2	3.04	110.58	106.92
2	A	501	TAU	O2-S-O3	-2.91	104.16	111.27
2	A	501	TAU	O1-S-C2	2.78	110.26	106.92
2	A	501	TAU	O2-S-C2	2.74	110.20	105.77
3	A	502	JN3	C22-C20-C17	-2.72	104.67	110.28
2	D	401	TAU	O2-S-C2	2.66	110.07	105.77
3	A	503	JN3	C1-C10-C5	2.65	111.69	107.77
2	B	401	TAU	O2-S-C2	2.65	110.05	105.77
2	A	501	TAU	O3-S-C2	2.62	110.07	106.92
3	D	402	JN3	C16-C17-C13	-2.59	100.72	103.84
2	D	401	TAU	O3-S-C2	2.54	109.98	106.92
2	D	401	TAU	O1-S-C2	2.54	109.97	106.92
3	A	502	JN3	C1-C10-C5	2.49	111.45	107.77
3	A	502	JN3	C4-C5-C10	2.44	115.25	112.66
2	C	401	TAU	O2-S-C2	2.41	109.67	105.77
3	A	503	JN3	C23-C22-C20	-2.39	111.50	114.72
3	A	503	JN3	C21-C20-C17	-2.33	109.35	112.92
3	D	402	JN3	C1-C10-C5	2.30	111.17	107.77
3	A	502	JN3	C9-C8-C7	-2.28	109.15	111.88
3	A	502	JN3	C4-C3-C2	-2.19	107.94	110.55
3	A	502	JN3	C17-C13-C14	-2.13	97.55	100.07
3	D	402	JN3	C19-C10-C1	-2.12	104.84	108.26

There are no chirality outliers.

All (17) torsion outliers are listed below:

Mol	Chain	Res	Type	Atoms
2	A	501	TAU	C1-C2-S-O1
2	D	401	TAU	N1-C1-C2-S
3	D	402	JN3	C21-C20-C22-C23
3	D	402	JN3	C16-C17-C20-C22
3	A	502	JN3	C13-C17-C20-C21
3	A	502	JN3	C16-C17-C20-C22
3	D	402	JN3	C17-C20-C22-C23
3	D	402	JN3	C13-C17-C20-C22
3	A	502	JN3	C17-C20-C22-C23

Continued on next page...

CONFIDENTIAL VALIDATION REPORT

Continued from previous page...

Mol	Chain	Res	Type	Atoms
3	A	502	JN3	C21-C20-C22-C23
3	D	402	JN3	C20-C22-C23-C24
3	D	402	JN3	C16-C17-C20-C21
2	A	501	TAU	C1-C2-S-O2
3	A	502	JN3	C16-C17-C20-C21
3	A	502	JN3	C13-C17-C20-C22
2	A	501	TAU	C1-C2-S-O3
2	A	501	TAU	N1-C1-C2-S

There are no ring outliers.

7 monomers are involved in 15 short contacts:

Mol	Chain	Res	Type	Clashes	Symm-Clashes
2	B	401	TAU	1	0
3	D	402	JN3	6	0
2	C	401	TAU	2	0
3	A	503	JN3	1	0
3	A	502	JN3	3	0
2	D	401	TAU	1	0
2	A	501	TAU	1	0

The following is a two-dimensional graphical depiction of Mogul quality analysis of bond lengths, bond angles, torsion angles, and ring geometry for all instances of the Ligand of Interest. In addition, ligands with molecular weight > 250 and outliers as shown on the validation Tables will also be included. For torsion angles, if less then 5% of the Mogul distribution of torsion angles is within 10 degrees of the torsion angle in question, then that torsion angle is considered an outlier. Any bond that is central to one or more torsion angles identified as an outlier by Mogul will be highlighted in the graph. For rings, the root-mean-square deviation (RMSD) between the ring in question and similar rings identified by Mogul is calculated over all ring torsion angles. If the average RMSD is greater than 60 degrees and the minimal RMSD between the ring in question and any Mogul-identified rings is also greater than 60 degrees, then that ring is considered an outlier. The outliers are highlighted in purple. The color gray indicates Mogul did not find sufficient equivalents in the CSD to analyse the geometry.

CONFIDENTIAL VALIDATION REPORT

Ligand TAU B 401

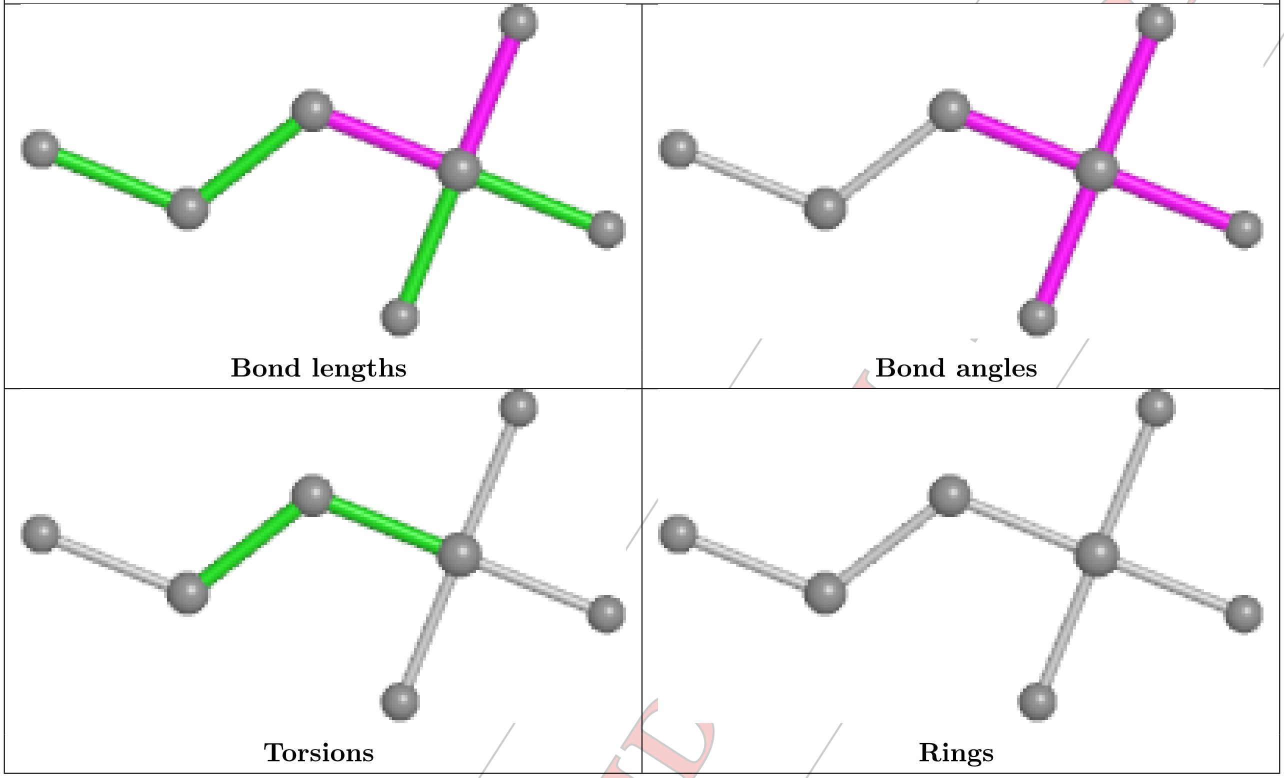

Ligand JN3 D 402

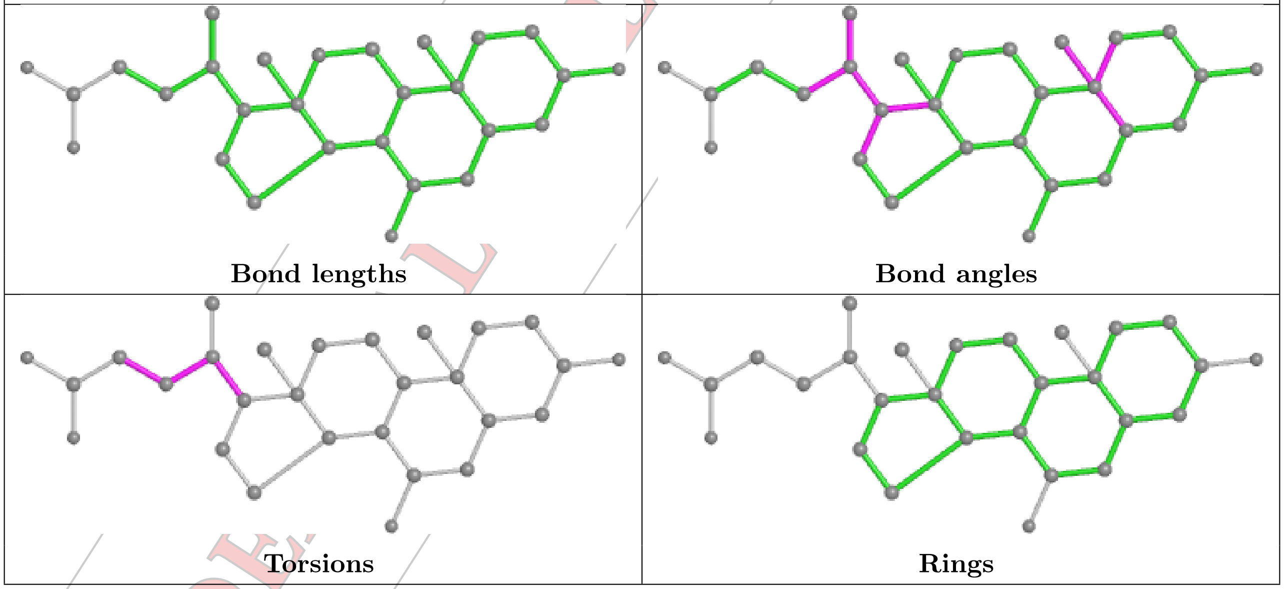

Ligand TAU C 401

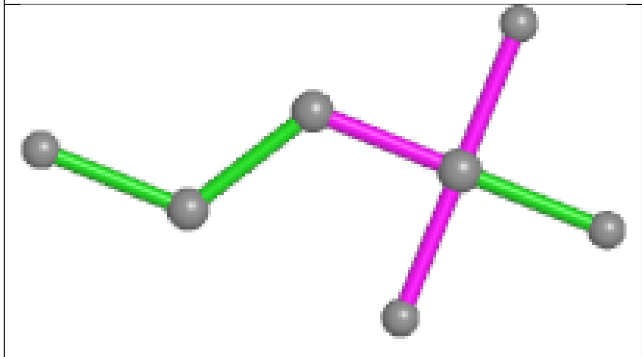

Bond lengths

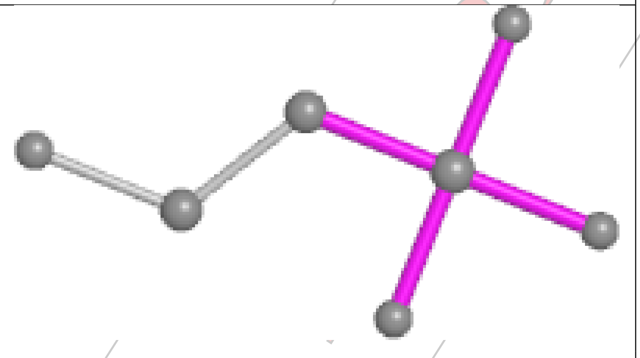

Bond angles

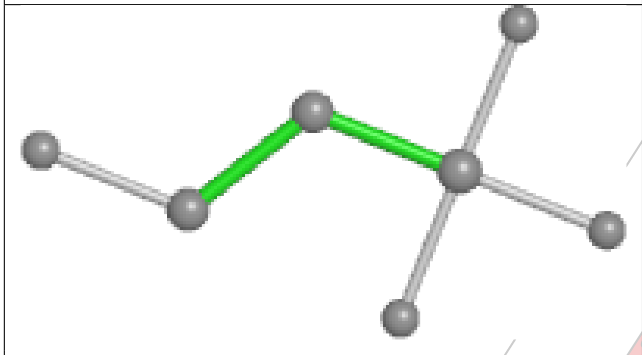

Torsions

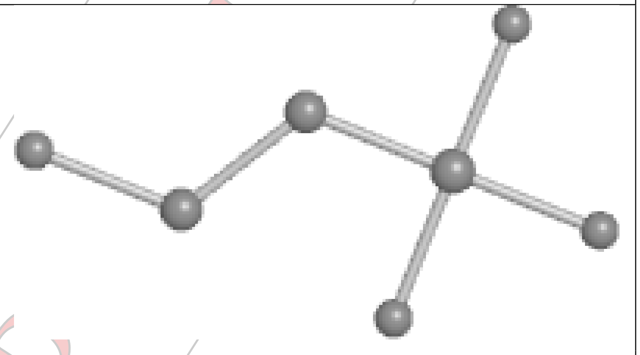

Rings

Ligand JN3 A 503

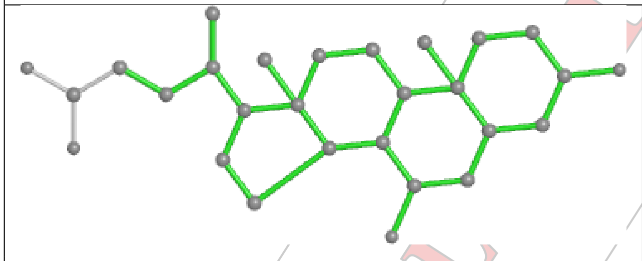

Bond lengths

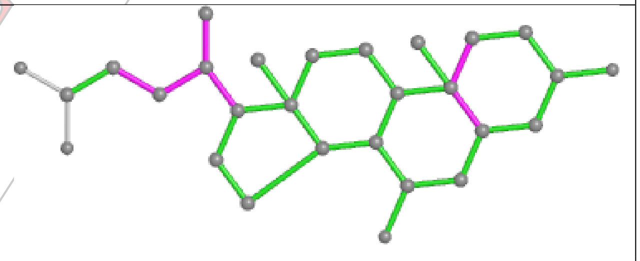

Bond angles

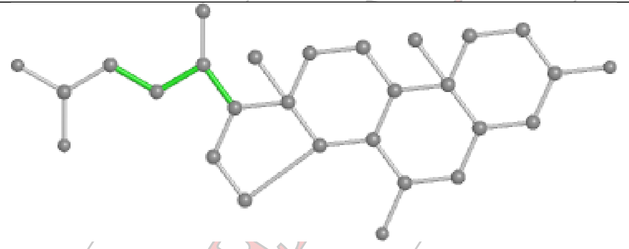

Torsions

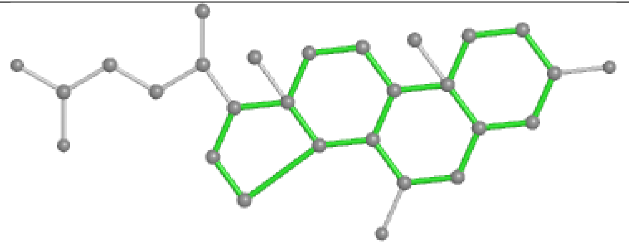

Rings

Ligand JN3 A 502

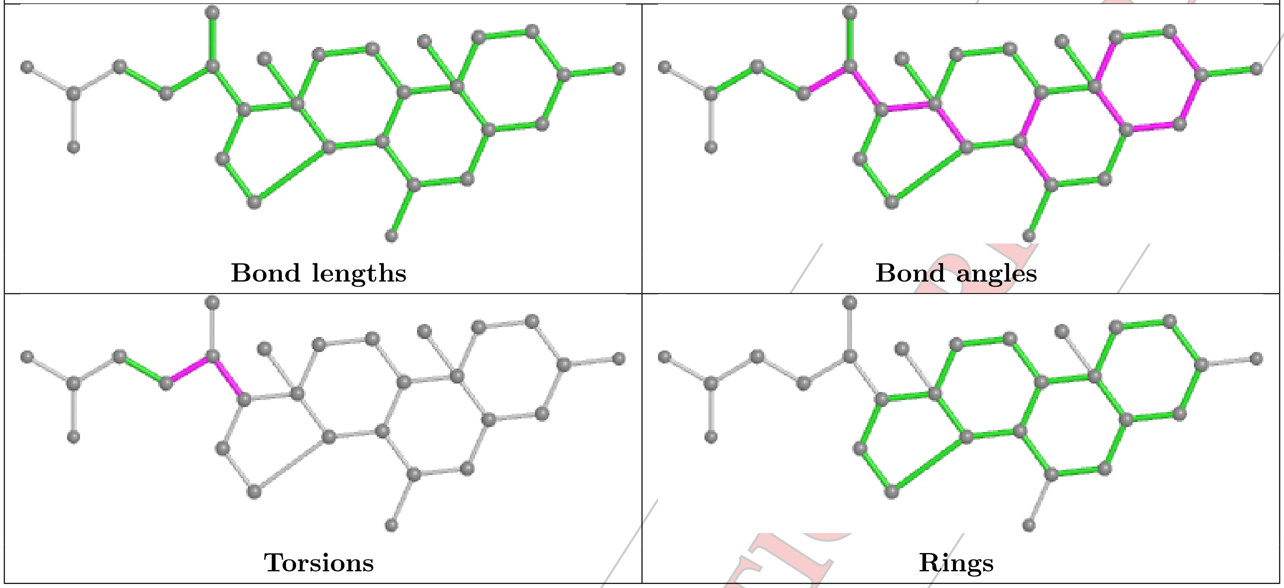

Ligand TAU D 401

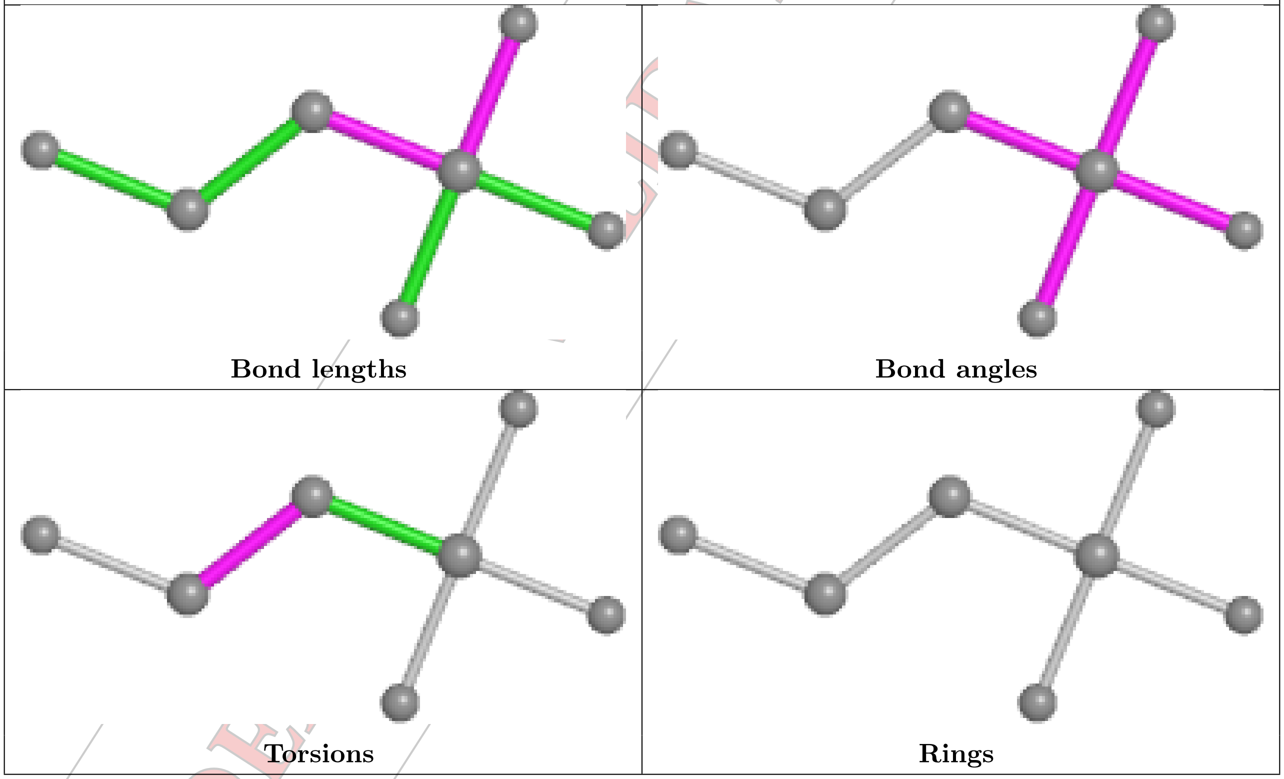

Ligand TAU A 501

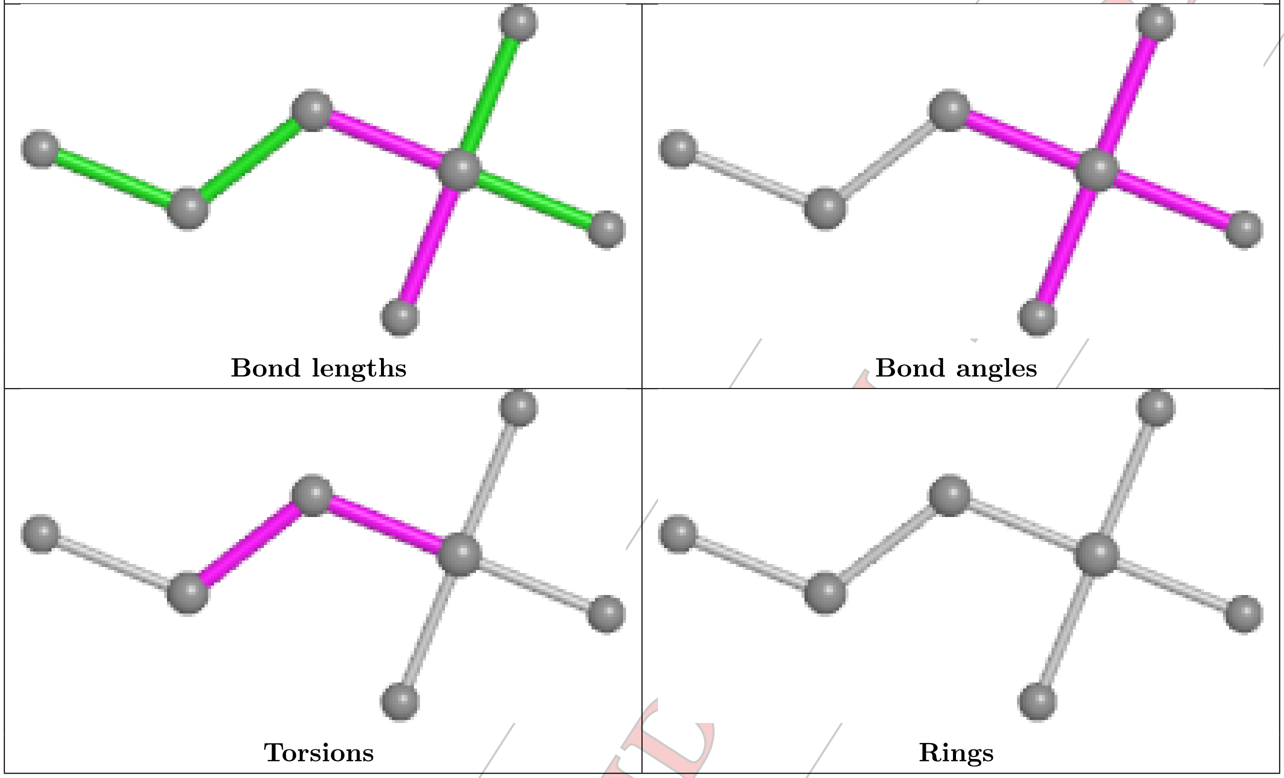

5.7 Other polymers (i)

There are no such residues in this entry.

5.8 Polymer linkage issues (i)

There are no chain breaks in this entry.

CONFIDENTIAL VALI

6 Fit of model and data (i)

6.1 Protein, DNA and RNA chains (i)

In the following table, the column labelled '#RSRZ> 2' contains the number (and percentage) of RSRZ outliers, followed by percent RSRZ outliers for the chain as percentile scores relative to all X-ray entries and entries of similar resolution. The OWAB column contains the minimum, median, 95th percentile and maximum values of the occupancy-weighted average B-factor per residue. The column labelled 'Q< 0.9' lists the number of (and percentage) of residues with an average occupancy less than 0.9.

Mol	Chain	Analysed	<RSRZ>	#RSRZ>2			OWAB(Å^2)	Q<0.9
1	A	315/322 (97%)	0.46	18 (5%)	23	26	17, 22, 32, 43	0
1	B	314/322 (97%)	0.62	30 (9%)	8	10	18, 24, 38, 46	0
1	C	314/322 (97%)	0.43	21 (6%)	17	20	18, 23, 33, 42	0
1	D	314/322 (97%)	0.35	12 (3%)	40	45	17, 22, 31, 40	0
All	All	1257/1288 (97%)	0.47	81 (6%)	19	21	17, 22, 34, 46	0

All (81) RSRZ outliers are listed below:

Mol	Chain	Res	Type	RSRZ
1	B	135	SER	6.2
1	A	317	HIS	6.1
1	B	93	ILE	6.0
1	B	136	PHE	5.7
1	B	94	ASP	5.4
1	C	26	TYR	4.6
1	B	95	GLY	4.6
1	B	134	SER	4.6
1	A	311	ASP	4.2
1	B	90	ASP	4.1
1	B	26	TYR	3.7
1	A	94	ASP	3.7
1	C	269	LEU	3.6
1	D	94	ASP	3.4
1	B	10	ASP	3.4
1	C	27	GLY	3.3
1	B	311	ASP	3.3
1	B	69	CYS	3.2
1	C	93	ILE	3.2
1	C	95	GLY	3.1
1	A	95	GLY	3.0

Continued on next page...

CONFIDENTIAL VALIDATION REPORT

Continued from previous page...

Mol	Chain	Res	Type	RSRZ
1	B	61	ASP	3.0
1	B	11	GLN	3.0
1	D	10	ASP	2.9
1	C	94	ASP	2.9
1	B	27	GLY	2.9
1	A	144	ILE	2.9
1	C	90	ASP	2.7
1	A	25	ASP	2.7
1	C	305	ASP	2.7
1	B	316	TYR	2.7
1	C	135	SER	2.6
1	C	77	ILE	2.6
1	D	311	ASP	2.6
1	D	106	LEU	2.6
1	A	10	ASP	2.6
1	B	305	ASP	2.6
1	C	106	LEU	2.6
1	B	124	ASN	2.6
1	B	269	LEU	2.6
1	D	61	ASP	2.5
1	A	90	ASP	2.5
1	B	314	ILE	2.5
1	C	104	ILE	2.5
1	C	144	ILE	2.4
1	A	24	GLN	2.4
1	A	70	PHE	2.4
1	D	145	ILE	2.4
1	C	69	CYS	2.4
1	A	132	ILE	2.3
1	A	306	GLU	2.3
1	D	69	CYS	2.3
1	D	255	LEU	2.3
1	C	10	ASP	2.3
1	B	205	LYS	2.3
1	A	11	GLN	2.3
1	B	315	THR	2.3
1	C	12	GLY	2.3
1	C	11	GLN	2.2
1	C	198	ALA	2.2
1	B	268	LYS	2.2
1	A	61	ASP	2.2
1	A	305	ASP	2.2

Continued on next page...

CONFIDENTIAL VALIDATION REPORT

Continued from previous page...

Mol	Chain	Res	Type	RSRZ
1	C	80	LEU	2.2
1	B	104	ILE	2.2
1	D	93	ILE	2.2
1	A	16	PHE	2.1
1	A	106	LEU	2.1
1	D	234	LEU	2.1
1	B	144	ILE	2.1
1	D	254	ILE	2.1
1	B	70	PHE	2.1
1	B	132	ILE	2.1
1	A	316	TYR	2.0
1	C	25	ASP	2.0
1	D	305	ASP	2.0
1	B	313	LEU	2.0
1	B	225	ALA	2.0
1	B	133	ASN	2.0
1	C	120	GLU	2.0
1	B	80	LEU	2.0

6.2 Non-standard residues in protein, DNA, RNA chains (i)

In the following table, the Atoms column lists the number of modelled atoms in the group and the number defined in the chemical component dictionary. The B-factors column lists the minimum, median, 95th percentile and maximum values of B factors of atoms in the group. The column labelled 'Q< 0.9' lists the number of atoms with occupancy less than 0.9.

Mol	Type	Chain	Res	Atoms	RSCC	RSR	B-factors(Å^2)	Q<0.9
1	OCS	A	2	9/10	0.98	0.11	18,19,24,24	3
1	OCS	B	2	9/10	0.98	0.10	19,22,25,26	3
1	OCS	C	2	9/10	0.98	0.09	20,21,25,26	3
1	OCS	D	2	9/10	0.98	0.09	17,21,25,26	3

6.3 Carbohydrates (i)

There are no monosaccharides in this entry.

6.4 Ligands (i)

In the following table, the Atoms column lists the number of modelled atoms in the group and the number defined in the chemical component dictionary. The B-factors column lists the minimum,

CONFIDENTIAL VALIDATION REPORT

median, 95th percentile and maximum values of B factors of atoms in the group. The column labelled 'Q< 0.9' lists the number of atoms with occupancy less than 0.9.

Mol	Type	Chain	Res	Atoms	RSCC	RSR	B-factors($Å^2$)	Q<0.9
3	JN3	A	502	28/28	0.78	0.15	20,28,37,43	67
3	JN3	D	402	28/28	0.78	0.14	22,30,38,41	67
3	JN3	A	503	28/28	0.79	0.16	24,32,39,42	67
2	TAU	B	401	7/7	0.96	0.13	22,24,29,30	7
2	TAU	A	501	7/7	0.97	0.11	21,24,31,32	7
2	TAU	D	401	7/7	0.98	0.09	24,27,33,33	0
2	TAU	C	401	7/7	0.98	0.11	22,25,29,31	7

The following is a graphical depiction of the model fit to experimental electron density of all instances of the Ligand of Interest. In addition, ligands with molecular weight > 250 and outliers as shown on the geometry validation Tables will also be included. Each fit is shown from different orientation to approximate a three-dimensional view.

Electron density around JN3 A 502:

2mF$_o$-DF$_c$ (at 0.7 rmsd) in gray
mF$_o$-DF$_c$ (at 3 rmsd) in purple (negative)
and green (positive)

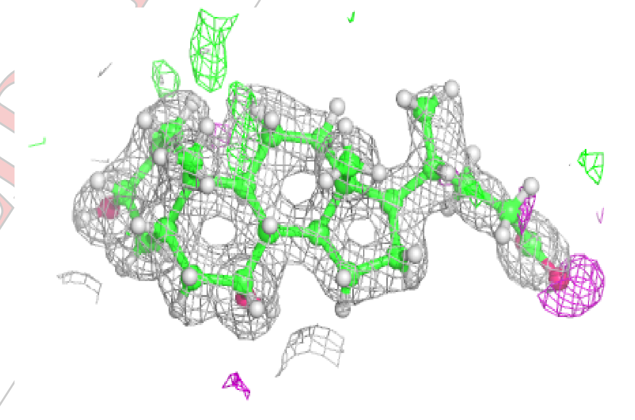

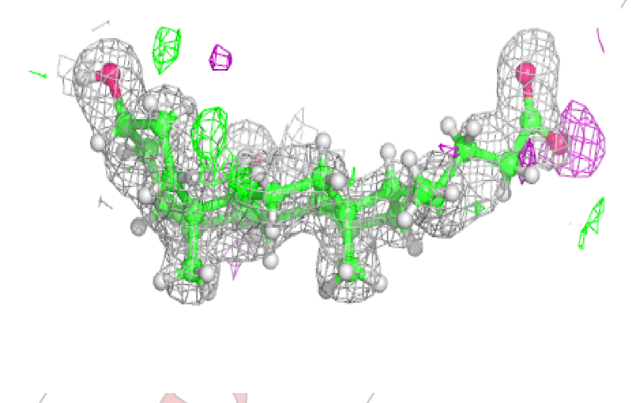

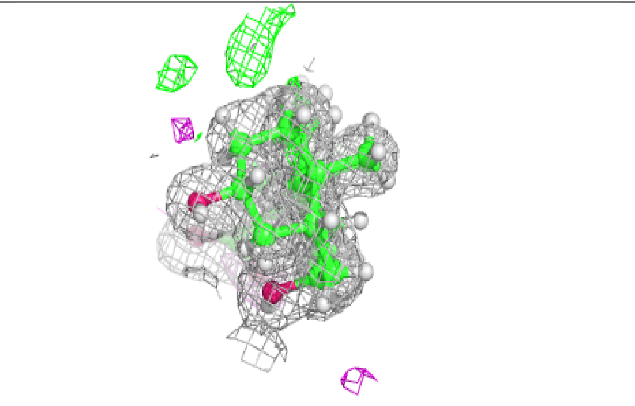

CONFIDENTIAL VALIDATION REPORT

Electron density around JN3 D 402:

$2mF_o$-DF_c (at 0.7 rmsd) in gray
mF_o-DF_c (at 3 rmsd) in purple (negative)
and green (positive)

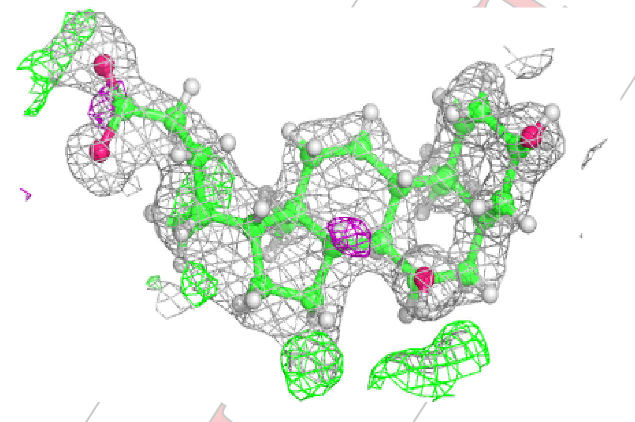

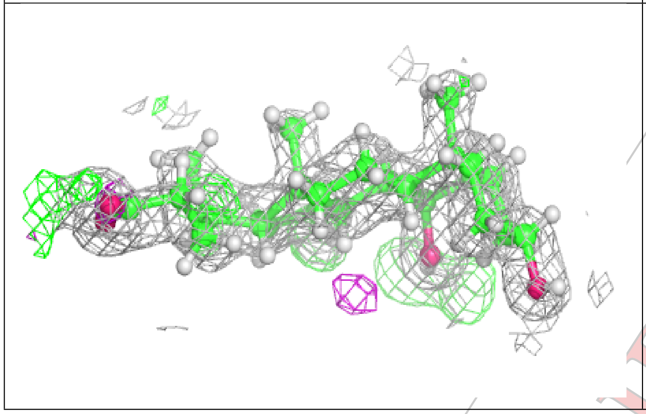

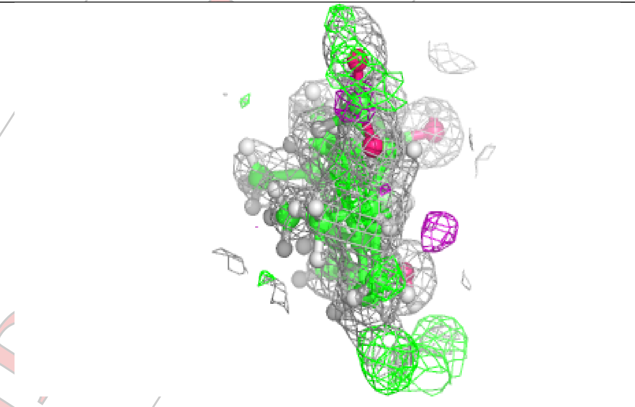

Electron density around JN3 A 503:

$2mF_o$-DF_c (at 0.7 rmsd) in gray
mF_o-DF_c (at 3 rmsd) in purple (negative)
and green (positive)

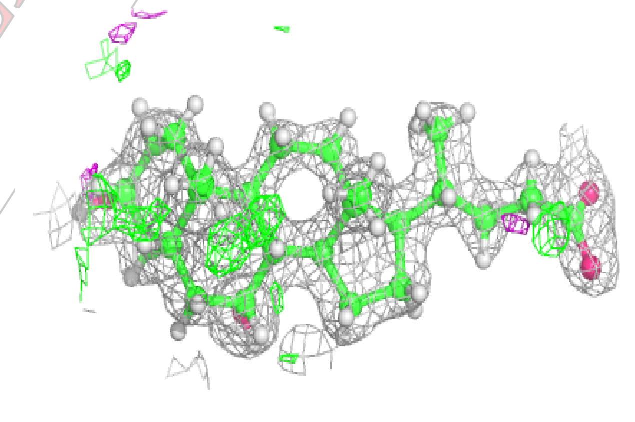

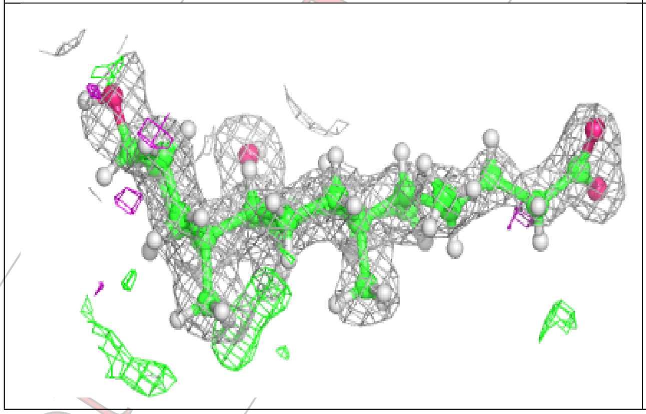

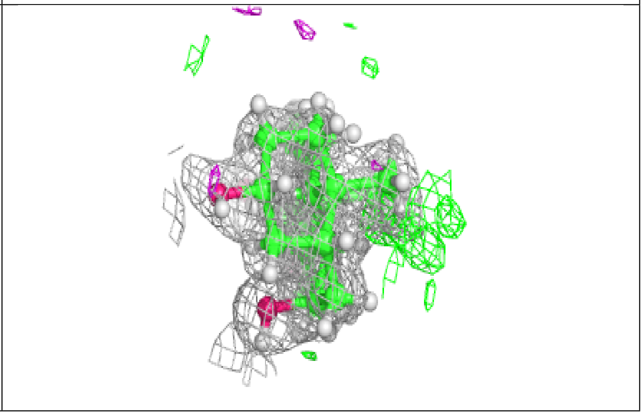

Electron density around TAU B 401:

$2mF_o$-DF_c (at 0.7 rmsd) in gray
mF_o-DF_c (at 3 rmsd) in purple (negative)
and green (positive)

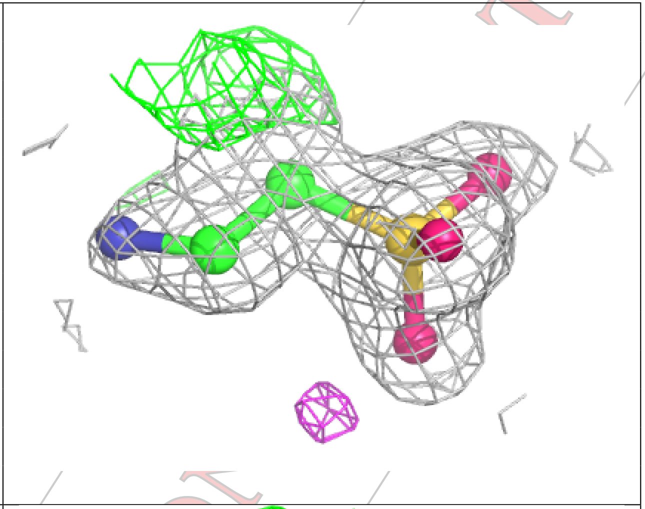

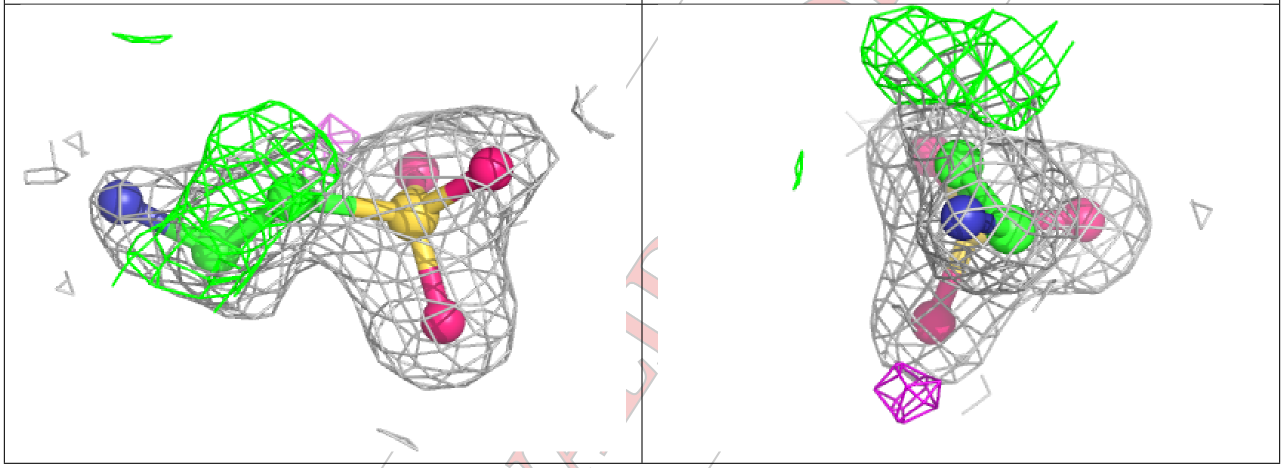

CONFIDENTIAL

Electron density around TAU A 501:

$2mF_o$-DF_c (at 0.7 rmsd) in gray
mF_o-DF_c (at 3 rmsd) in purple (negative)
and green (positive)

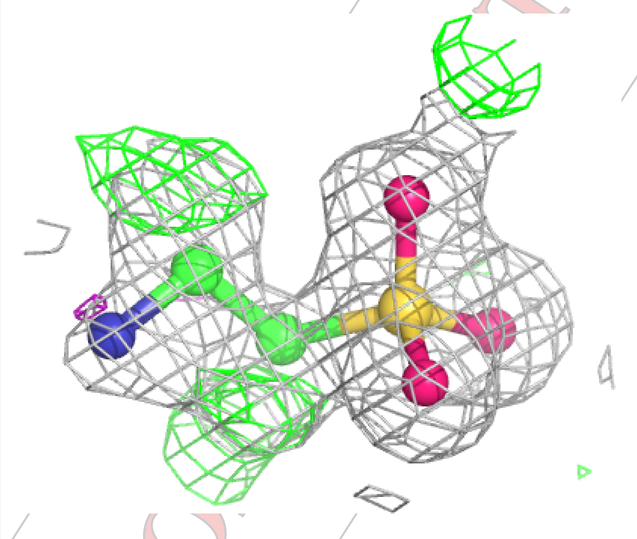

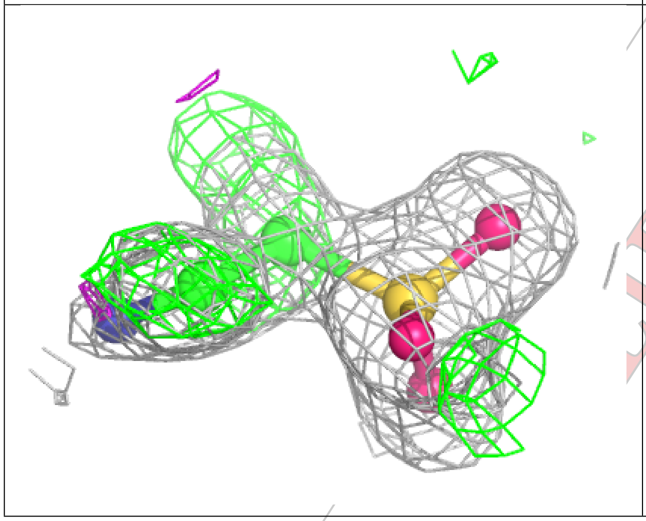

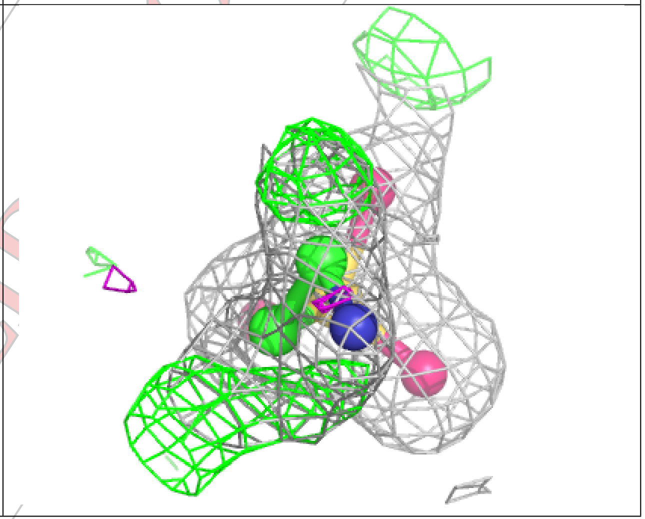

CONFIDENTIAL

Electron density around TAU D 401:

2mF$_o$-DF$_c$ (at 0.7 rmsd) in gray
mF$_o$-DF$_c$ (at 3 rmsd) in purple (negative)
and green (positive)

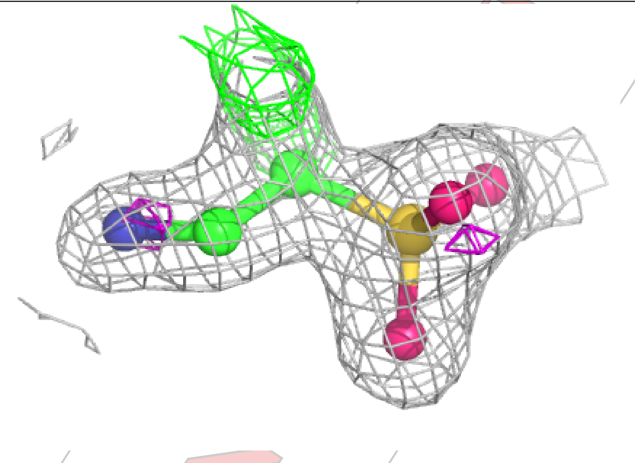

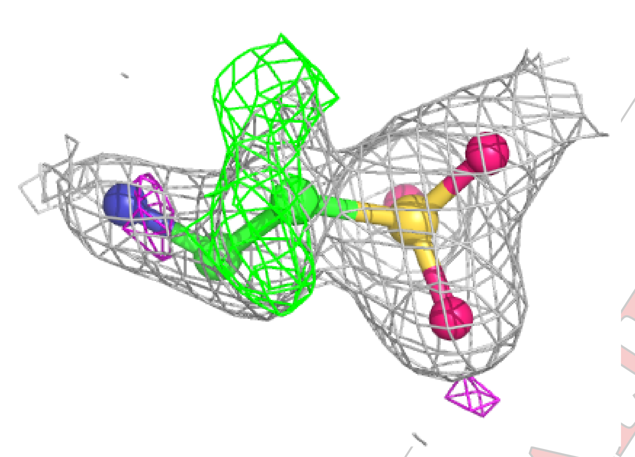

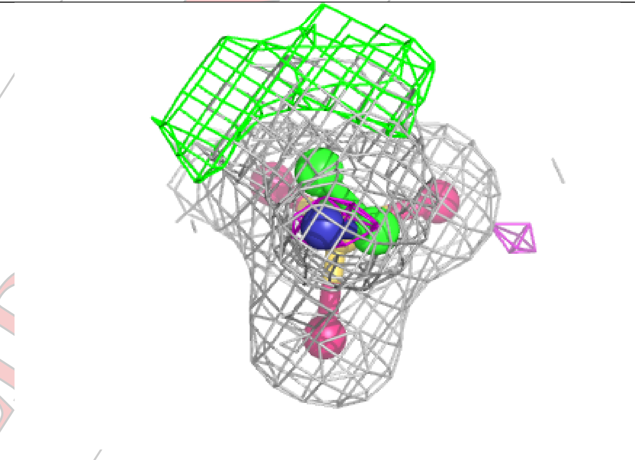

Electron density around TAU C 401:

2mF$_o$-DF$_c$ (at 0.7 rmsd) in gray
mF$_o$-DF$_c$ (at 3 rmsd) in purple (negative)
and green (positive)

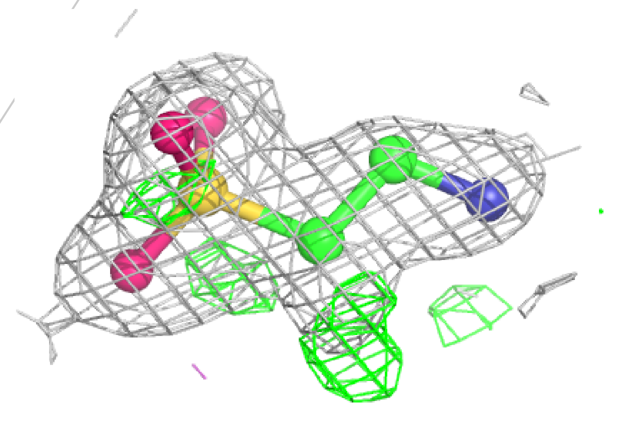

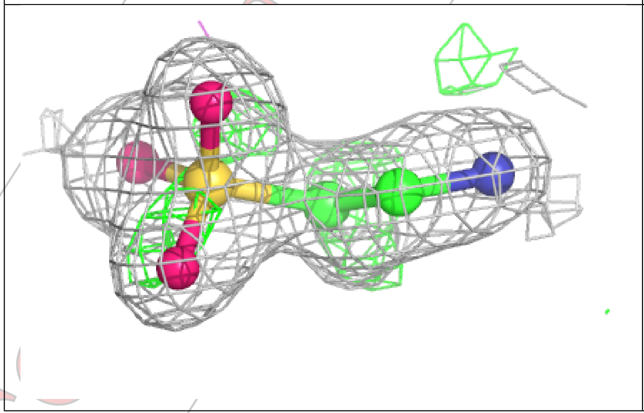

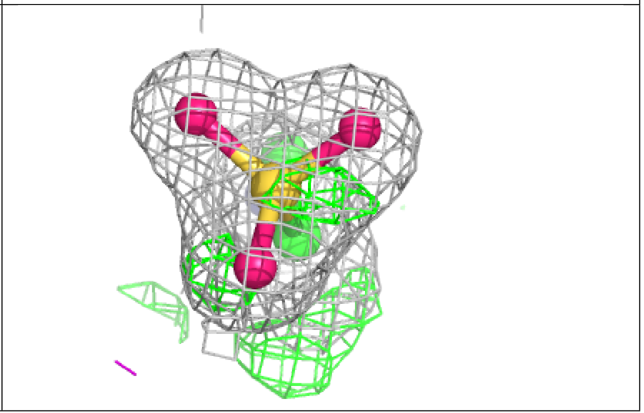

6.5 Other polymers (i)

There are no such residues in this entry.

CONFIDENTIAL VALIDATION REPORT
