# Supplementary material for: Bile salt hydrolases shape the bile acid landscape and restrict Clostridioides difficile growth in the murine gut
Source: Nat Microbiol. 2023 Mar 13;8(4):611–28. doi: 10.1038/s41564-023-01337-7 (PMC10066039; doi:10.1038/s41564-023-01337-7)
Supplement: Supplementary file 9 — 7SVH PDB validation report. [file 41564_2023_1337_MOESM9_ESM.pdf]

Full wwPDB X-ray Structure Validation Report (i)

Nov 22, 2021 – 11:53 AM EST

PDB ID	:	7SVH
Title	:	Bile Salt Hydrolase B from Lactobacillus gasseri
Deposited on	:	2021-11-19
Resolution	:	1.56 Å(reported)

This is a Full wwPDB X-ray Structure Validation Report.

This report is produced by the wwPDB biocuration pipeline after annotation of the structure.

We welcome your comments at *validation@mail.wwpdb.org*
A user guide is available at
https://www.wwpdb.org/validation/2017/XrayValidationReportHelp
with specific help available everywhere you see the (i) symbol.

CONFIDENTIAL VALIDATION REPORT

The following versions of software and data (see references (i)) were used in the production of this report:

MolProbity	:	4.02b-467
Xtriage (Phenix)	:	1.13
EDS	:	2.23.2
Percentile statistics	:	20191225.v01 (using entries in the PDB archive December 25th 2019)
Refmac	:	5.8.0158
CCP4	:	7.0.044 (Gargrove)
Ideal geometry (proteins)	:	Engh & Huber (2001)
Ideal geometry (DNA, RNA)	:	Parkinson et al. (1996)
Validation Pipeline (wwPDB-VP)	:	2.23.2

1 Overall quality at a glance (i)

The following experimental techniques were used to determine the structure:
X-RAY DIFFRACTION

The reported resolution of this entry is 1.56 Å.

Percentile scores (ranging between 0-100) for global validation metrics of the entry are shown in the following graphic. The table shows the number of entries on which the scores are based.

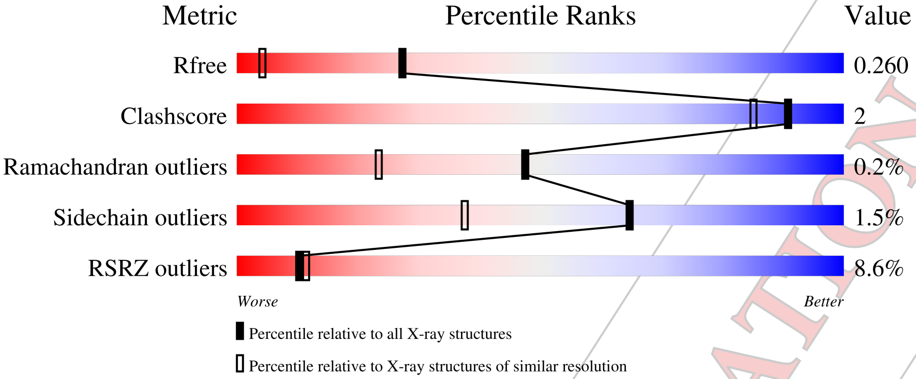

Metric	Whole archive (#Entries)	Similar resolution (#Entries, resolution range(Å))
R_{free}	130704	1483 (1.56-1.56)
Clashscore	141614	1529 (1.56-1.56)
Ramachandran outliers	138981	1498 (1.56-1.56)
Sidechain outliers	138945	1495 (1.56-1.56)
RSRZ outliers	127900	1465 (1.56-1.56)

The table below summarises the geometric issues observed across the polymeric chains and their fit to the electron density. The red, orange, yellow and green segments of the lower bar indicate the fraction of residues that contain outliers for >=3, 2, 1 and 0 types of geometric quality criteria respectively. A grey segment represents the fraction of residues that are not modelled. The numeric value for each fraction is indicated below the corresponding segment, with a dot representing fractions <=5% The upper red bar (where present) indicates the fraction of residues that have poor fit to the electron density. The numeric value is given above the bar.

Mol	Chain	Length	Quality of chain
1	A	331	7% / 92% 6% •
1	B	331	10% / 92% 5% •
1	C	331	4% / 95% 5% •
1	D	331	5% / 92% 5% •
1	E	331	11% / 90% 8% •

Continued on next page...

CONFIDENTIAL VALIDATION REPORT

Continued from previous page...

Mol	Chain	Length	Quality of chain
1	F	331	7% 94% 5% •
1	G	331	13% 91% 6% •
1	H	331	10% 92% 5% •

CONFIDENTIAL VALIDATION REPORT

2 Entry composition (i)

There are 3 unique types of molecules in this entry. The entry contains 22086 atoms, of which 0 are hydrogens and 0 are deuteriums.

In the tables below, the ZeroOcc column contains the number of atoms modelled with zero occupancy, the AltConf column contains the number of residues with at least one atom in alternate conformation and the Trace column contains the number of residues modelled with at most 2 atoms.

- Molecule 1 is a protein called Choloylglycine hydrolase.

Mol	Chain	Residues	Atoms					ZeroOcc	AltConf	Trace
1	A	324	Total 2515	C 1606	N 403	O 494	S 12	0	0	0
1	B	324	Total 2520	C 1609	N 405	O 494	S 12	0	0	0
1	C	329	Total 2553	C 1633	N 418	O 490	S 12	0	0	0
1	D	321	Total 2497	C 1598	N 401	O 486	S 12	0	0	0
1	E	324	Total 2521	C 1610	N 407	O 492	S 12	0	0	0
1	F	326	Total 2531	C 1615	N 409	O 495	S 12	0	0	0
1	G	321	Total 2494	C 1594	N 401	O 487	S 12	0	0	0
1	H	321	Total 2481	C 1589	N 399	O 481	S 12	0	0	0

There are 48 discrepancies between the modelled and reference sequences:

Chain	Residue	Modelled	Actual	Comment	Reference
A	326	HIS	-	expression tag	UNP A0A833FHE1
A	327	HIS	-	expression tag	UNP A0A833FHE1
A	328	HIS	-	expression tag	UNP A0A833FHE1
A	329	HIS	-	expression tag	UNP A0A833FHE1
A	330	HIS	-	expression tag	UNP A0A833FHE1
A	331	HIS	-	expression tag	UNP A0A833FHE1
B	326	HIS	-	expression tag	UNP A0A833FHE1
B	327	HIS	-	expression tag	UNP A0A833FHE1
B	328	HIS	-	expression tag	UNP A0A833FHE1
B	329	HIS	-	expression tag	UNP A0A833FHE1
B	330	HIS	-	expression tag	UNP A0A833FHE1
B	331	HIS	-	expression tag	UNP A0A833FHE1
C	326	HIS	-	expression tag	UNP A0A833FHE1

Continued on next page...

CONFIDENTIAL VALIDATION REPORT

Continued from previous page...

Chain	Residue	Modelled	Actual	Comment	Reference
C	327	HIS	-	expression tag	UNP A0A833FHE1
C	328	HIS	-	expression tag	UNP A0A833FHE1
C	329	HIS	-	expression tag	UNP A0A833FHE1
C	330	HIS	-	expression tag	UNP A0A833FHE1
C	331	HIS	-	expression tag	UNP A0A833FHE1
D	326	HIS	-	expression tag	UNP A0A833FHE1
D	327	HIS	-	expression tag	UNP A0A833FHE1
D	328	HIS	-	expression tag	UNP A0A833FHE1
D	329	HIS	-	expression tag	UNP A0A833FHE1
D	330	HIS	-	expression tag	UNP A0A833FHE1
D	331	HIS	-	expression tag	UNP A0A833FHE1
E	326	HIS	-	expression tag	UNP A0A833FHE1
E	327	HIS	-	expression tag	UNP A0A833FHE1
E	328	HIS	-	expression tag	UNP A0A833FHE1
E	329	HIS	-	expression tag	UNP A0A833FHE1
E	330	HIS	-	expression tag	UNP A0A833FHE1
E	331	HIS	-	expression tag	UNP A0A833FHE1
F	326	HIS	-	expression tag	UNP A0A833FHE1
F	327	HIS	-	expression tag	UNP A0A833FHE1
F	328	HIS	-	expression tag	UNP A0A833FHE1
F	329	HIS	-	expression tag	UNP A0A833FHE1
F	330	HIS	-	expression tag	UNP A0A833FHE1
F	331	HIS	-	expression tag	UNP A0A833FHE1
G	326	HIS	-	expression tag	UNP A0A833FHE1
G	327	HIS	-	expression tag	UNP A0A833FHE1
G	328	HIS	-	expression tag	UNP A0A833FHE1
G	329	HIS	-	expression tag	UNP A0A833FHE1
G	330	HIS	-	expression tag	UNP A0A833FHE1
G	331	HIS	-	expression tag	UNP A0A833FHE1
H	326	HIS	-	expression tag	UNP A0A833FHE1
H	327	HIS	-	expression tag	UNP A0A833FHE1
H	328	HIS	-	expression tag	UNP A0A833FHE1
H	329	HIS	-	expression tag	UNP A0A833FHE1
H	330	HIS	-	expression tag	UNP A0A833FHE1
H	331	HIS	-	expression tag	UNP A0A833FHE1

- Molecule 2 is MAGNESIUM ION (three-letter code: MG) (formula: Mg).

Mol	Chain	Residues	Atoms	ZeroOcc	AltConf
2	C	1	Total Mg<br>1 1	0	0
2	D	1	Total Mg<br>1 1	0	0

Continued on next page...

CONFIDENTIAL VALIDATION REPORT

Continued from previous page...

Mol	Chain	Residues	Atoms	ZeroOcc	AltConf
2	F	1	Total Mg<br>1 1	0	0
2	H	1	Total Mg<br>1 1	0	0

- Molecule 3 is water.

Mol	Chain	Residues	Atoms	ZeroOcc	AltConf
3	A	277	Total O<br>277 277	0	0
3	B	269	Total O<br>269 269	0	0
3	C	280	Total O<br>280 280	0	0
3	D	256	Total O<br>256 256	0	0
3	E	243	Total O<br>243 243	0	0
3	F	255	Total O<br>255 255	0	0
3	G	194	Total O<br>194 194	0	0
3	H	196	Total O<br>196 196	0	0

CONFIDENTIAL VALIDATION REPORT

3 Residue-property plots (i)

These plots are drawn for all protein, RNA, DNA and oligosaccharide chains in the entry. The first graphic for a chain summarises the proportions of the various outlier classes displayed in the second graphic. The second graphic shows the sequence view annotated by issues in geometry and electron density. Residues are color-coded according to the number of geometric quality criteria for which they contain at least one outlier: green = 0, yellow = 1, orange = 2 and red = 3 or more. A red dot above a residue indicates a poor fit to the electron density (RSRZ > 2). Stretches of 2 or more consecutive residues without any outlier are shown as a green connector. Residues present in the sample, but not in the model, are shown in grey.

- Molecule 1: Choloylglycine hydrolase

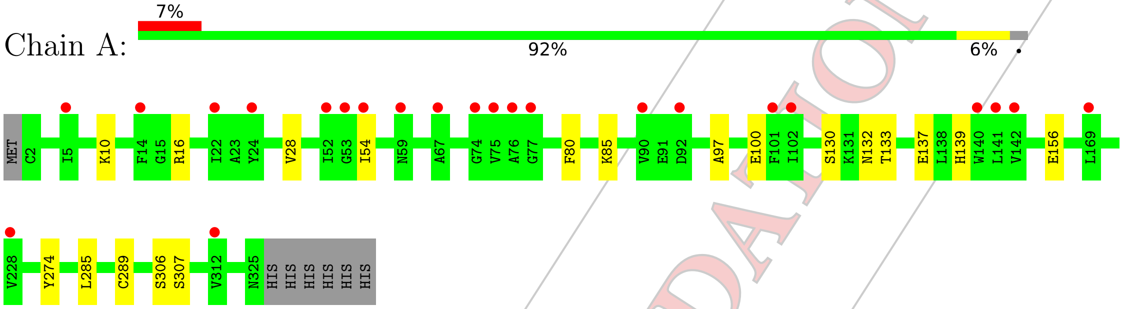

- Molecule 1: Choloylglycine hydrolase

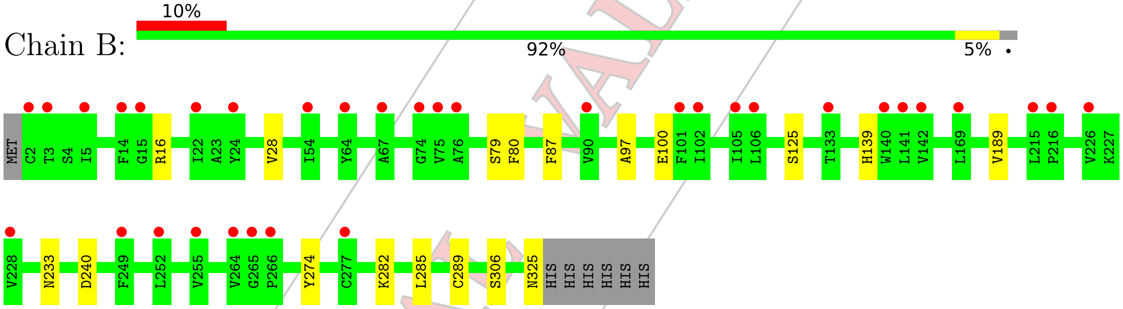

- Molecule 1: Choloylglycine hydrolase

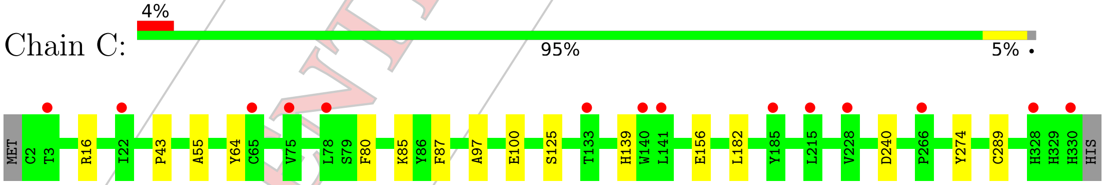

- Molecule 1: Choloylglycine hydrolase

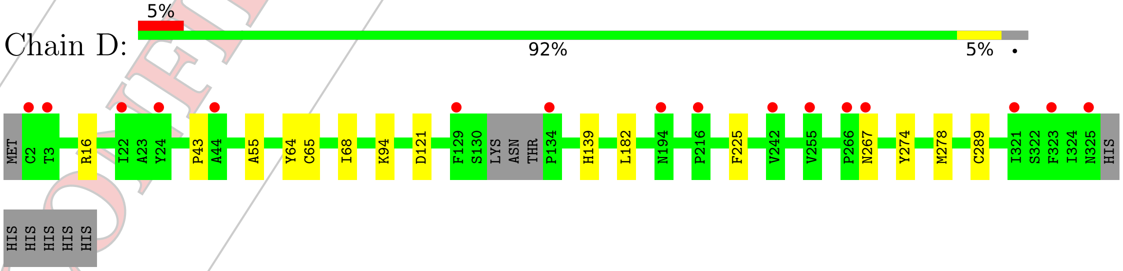

CONFIDENTIAL VALIDATION REPORT

- Molecule 1: Choloylglycine hydrolase

Chain E:

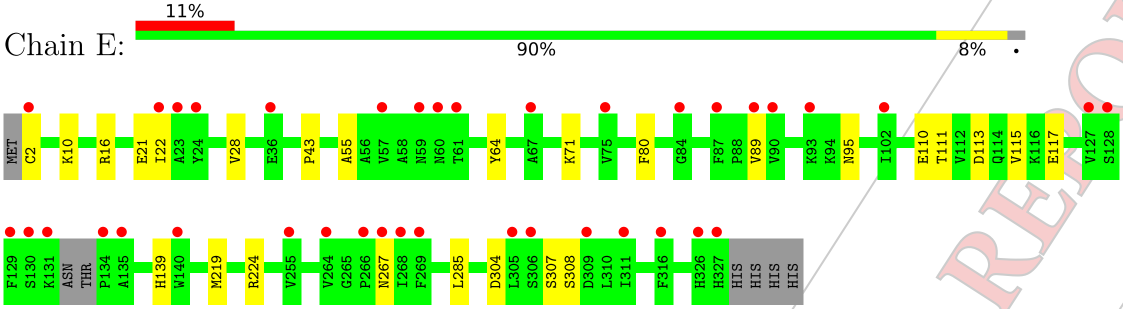

- Molecule 1: Choloylglycine hydrolase

Chain F:

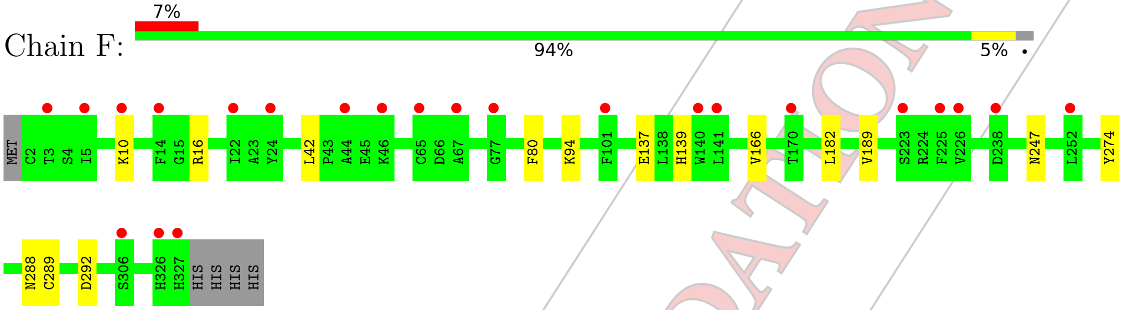

- Molecule 1: Choloylglycine hydrolase

Chain G:

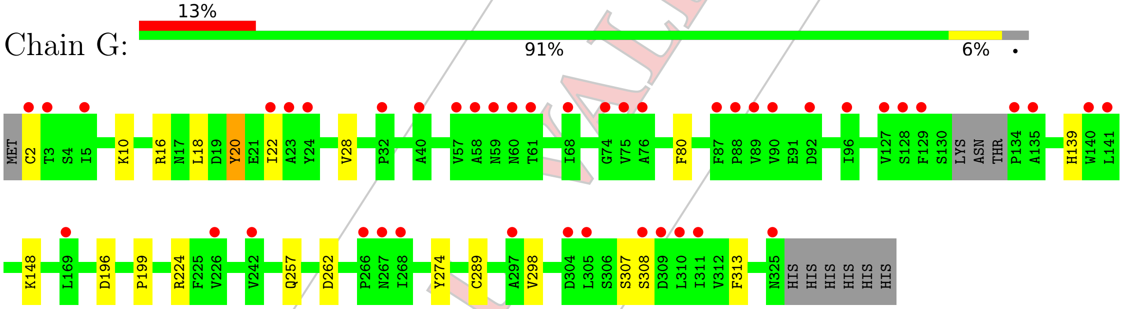

- Molecule 1: Choloylglycine hydrolase

Chain H:

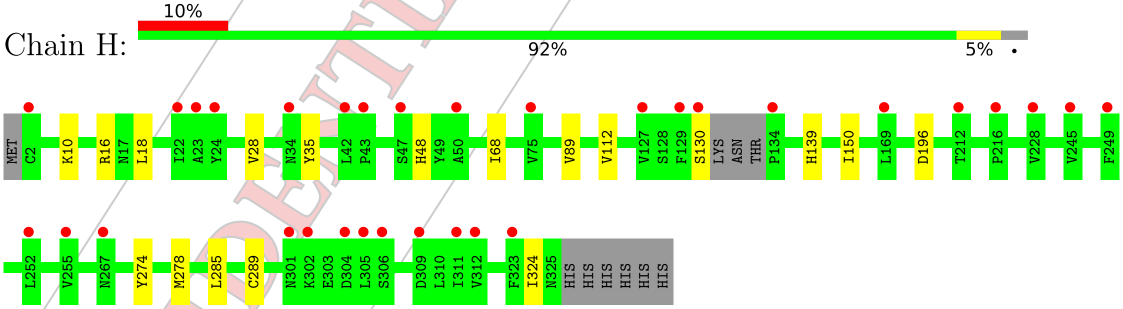

CONFIDENTIAL VALIDATION REPORT

4 Data and refinement statistics (i)

Property	Value	Source
Space group	P 1 21 1	Depositor
Cell constants<br>a, b, c, α, β, γ	87.35Å 167.38Å 88.15Å<br>90.00° 98.98° 90.00°	Depositor
Resolution (Å)	29.02 – 1.56<br>29.02 – 1.56	Depositor<br>EDS
% Data completeness<br>(in resolution range)	96.8 (29.02-1.56)<br>96.8 (29.02-1.56)	Depositor<br>EDS
R_{merge}	0.06	Depositor
R_{sym}	(Not available)	Depositor
$< I/\sigma(I) >$ [1]	1.00 (at 1.56Å)	Xtriage
Refinement program	PHENIX 1.17.1_3660, PHENIX 1.17.1_3660	Depositor
R, R_{free}	0.221 , 0.260<br>0.221 , 0.260	Depositor<br>DCC
R_{free} test set	1955 reflections (0.57%)	wwPDB-VP
Wilson B-factor (Å^2)	24.4	Xtriage
Anisotropy	0.425	Xtriage
Bulk solvent k_{sol}(e/Å^3), B_{sol}(Å^2)	0.36 , 48.2	EDS
L-test for twinning[2]	$< \|L\| >$ = 0.50, $< L^2 >$ = 0.34	Xtriage
Estimated twinning fraction	0.000 for l,-k,h	Xtriage
F_o,F_c correlation	0.97	EDS
Total number of atoms	22086	wwPDB-VP
Average B, all atoms (Å^2)	32.0	wwPDB-VP

Xtriage's analysis on translational NCS is as follows: *The analyses of the Patterson function reveals a significant off-origin peak that is 83.38 % of the origin peak, indicating pseudo-translational symmetry. The chance of finding a peak of this or larger height randomly in a structure without pseudo-translational symmetry is equal to 2.2014e-07. The detected translational NCS is most likely also responsible for the elevated intensity ratio.*

[1] Intensities estimated from amplitudes.

[2] Theoretical values of $< |L| >$, $< L^2 >$ for acentric reflections are 0.5, 0.333 respectively for untwinned datasets, and 0.375, 0.2 for perfectly twinned datasets.

CONFIDENTIAL VALIDATION REPORT

5 Model quality (i)

5.1 Standard geometry (i)

Bond lengths and bond angles in the following residue types are not validated in this section: MG

The Z score for a bond length (or angle) is the number of standard deviations the observed value is removed from the expected value. A bond length (or angle) with $|Z| > 5$ is considered an outlier worth inspection. RMSZ is the root-mean-square of all Z scores of the bond lengths (or angles).

Mol	Chain	Bond lengths		Bond angles	
		RMSZ	$\#\|Z\| > 5$	RMSZ	$\#\|Z\| > 5$
1	A	0.37	0/2575	0.53	0/3505
1	B	0.36	0/2580	0.54	0/3510
1	C	0.36	0/2617	0.53	0/3560
1	D	0.37	0/2556	0.53	0/3473
1	E	0.36	0/2582	0.54	0/3511
1	F	0.36	0/2593	0.53	0/3530
1	G	0.35	0/2553	0.53	0/3471
1	H	0.34	0/2540	0.51	0/3454
All	All	0.36	0/20596	0.53	0/28014

There are no bond length outliers.

There are no bond angle outliers.

There are no chirality outliers.

There are no planarity outliers.

5.2 Too-close contacts (i)

In the following table, the Non-H and H(model) columns list the number of non-hydrogen atoms and hydrogen atoms in the chain respectively. The H(added) column lists the number of hydrogen atoms added and optimized by MolProbity. The Clashes column lists the number of clashes within the asymmetric unit, whereas Symm-Clashes lists symmetry-related clashes.

Mol	Chain	Non-H	H(model)	H(added)	Clashes	Symm-Clashes
1	A	2515	0	2384	8	0
1	B	2520	0	2400	11	0
1	C	2553	0	2427	6	0
1	D	2497	0	2388	9	0
1	E	2521	0	2390	11	0

Continued on next page...

CONFIDENTIAL VALIDATION REPORT

Continued from previous page...

Mol	Chain	Non-H	H(model)	H(added)	Clashes	Symm-Clashes
1	F	2531	0	2395	7	0
1	G	2494	0	2377	9	0
1	H	2481	0	2363	10	0
2	C	1	0	0	0	0
2	D	1	0	0	0	0
2	F	1	0	0	0	0
2	H	1	0	0	0	0
3	A	277	0	0	2	1
3	B	269	0	0	5	0
3	C	280	0	0	1	1
3	D	256	0	0	4	0
3	E	243	0	0	0	0
3	F	255	0	0	2	0
3	G	194	0	0	1	0
3	H	196	0	0	3	0
All	All	22086	0	19124	66	1

The all-atom clashscore is defined as the number of clashes found per 1000 atoms (including hydrogen atoms). The all-atom clashscore for this structure is 2.

All (66) close contacts within the same asymmetric unit are listed below, sorted by their clash magnitude.

Atom-1	Atom-2	Interatomic distance (Å)	Clash overlap (Å)
1:B:282:LYS:NZ	3:B:402:HOH:O	2.15	0.80
1:D:65:CYS:SG	3:D:742:HOH:O	2.40	0.78
1:E:21:GLU:HG2	1:E:22:ILE:HG13	1.70	0.73
1:G:2:CYS:O	1:G:224:ARG:NH2	2.23	0.72
1:F:247:ASN:OD1	3:F:502:HOH:O	2.15	0.64
1:E:2:CYS:O	1:E:224:ARG:NH2	2.30	0.64
1:B:79:SER:OG	3:B:403:HOH:O	2.15	0.63
1:F:137:GLU:OE1	3:F:503:HOH:O	2.16	0.63
1:E:113:ASP:O	1:E:117:GLU:HG2	2.00	0.62
1:A:137:GLU:OE1	3:A:401:HOH:O	2.16	0.60
1:A:306:SER:OG	3:A:402:HOH:O	2.16	0.60
1:A:85:LYS:HD3	1:A:156:GLU:HA	1.87	0.57
1:H:89:VAL:O	3:H:501:HOH:O	2.18	0.57
1:F:42:LEU:HD21	1:F:94:LYS:HG3	1.87	0.56
1:C:43:PRO:O	3:C:501:HOH:O	2.18	0.55
1:B:306:SER:OG	3:B:404:HOH:O	2.18	0.55
1:B:240:ASP:OD2	3:B:405:HOH:O	2.18	0.54
1:D:267:ASN:ND2	3:D:507:HOH:O	2.34	0.53

Continued on next page...

CONFIDENTIAL VALIDATION REPORT

Continued from previous page...

Atom-1	Atom-2	Interatomic distance (Å)	Clash overlap (Å)
1:G:148:LYS:HE2	3:G:580:HOH:O	2.08	0.53
1:E:71:LYS:HG3	1:E:110:GLU:HG2	1.92	0.52
1:B:28:VAL:HG13	1:B:285:LEU:HD23	1.92	0.50
1:D:121:ASP:HB3	1:E:43:PRO:HG3	1.95	0.49
1:D:182:LEU:HG	1:D:225:PHE:HE2	1.77	0.49
1:H:274:TYR:HA	1:H:289:CYS:HA	1.96	0.48
1:F:189:VAL:HG12	1:H:196:ASP:HB2	1.95	0.47
1:H:130:SER:O	3:H:502:HOH:O	2.20	0.47
1:B:325:ASN:O	3:B:406:HOH:O	2.21	0.47
1:F:166:VAL:HG11	1:F:182:LEU:HD11	1.96	0.46
1:D:68:ILE:HD13	1:D:278:MET:HB3	1.98	0.46
1:E:55:ALA:HB2	1:E:64:TYR:CD1	2.51	0.46
1:G:20:TYR:CD2	1:G:22:ILE:HG22	2.50	0.46
1:A:97:ALA:HB3	1:A:100:GLU:HG2	1.98	0.46
1:E:219:MET:O	1:E:224:ARG:HD3	2.16	0.46
1:D:55:ALA:HB2	1:D:64:TYR:CD1	2.51	0.45
1:H:112:VAL:HG13	1:H:150:ILE:HG12	1.98	0.45
1:B:274:TYR:HA	1:B:289:CYS:HA	1.98	0.45
1:C:274:TYR:HA	1:C:289:CYS:HA	1.99	0.45
1:A:28:VAL:HG13	1:A:285:LEU:HD23	1.99	0.45
1:H:35:TYR:HA	3:H:532:HOH:O	2.16	0.45
1:A:274:TYR:HA	1:A:289:CYS:HA	1.98	0.45
1:H:35:TYR:O	1:H:48:HIS:NE2	2.43	0.44
1:F:274:TYR:HA	1:F:289:CYS:HA	2.00	0.44
1:E:28:VAL:HG13	1:E:285:LEU:HD23	1.99	0.44
1:B:97:ALA:HB3	1:B:100:GLU:HG2	2.00	0.44
1:D:43:PRO:O	3:D:502:HOH:O	2.21	0.43
1:C:97:ALA:HB3	1:C:100:GLU:HG2	2.01	0.43
1:G:257:GLN:NE2	1:G:262:ASP:OD2	2.41	0.43
1:E:111:THR:O	1:E:115:VAL:HG23	2.19	0.42
1:E:89:VAL:HG13	1:E:95:ASN:CG	2.39	0.42
1:G:28:VAL:HB	1:G:313:PHE:HB2	2.00	0.42
1:H:28:VAL:HG13	1:H:285:LEU:HD23	2.00	0.42
1:C:87:PHE:O	1:C:125:SER:HA	2.20	0.42
1:G:274:TYR:HA	1:G:289:CYS:HA	2.01	0.42
1:B:233:ASN:OD1	1:G:199:PRO:HD2	2.20	0.42
1:C:85:LYS:HD3	1:C:156:GLU:HA	2.01	0.42
1:F:288:ASN:HB2	1:F:292:ASP:O	2.20	0.42
1:E:304:ASP:O	1:E:307:SER:OG	2.30	0.42
1:D:274:TYR:HA	1:D:289:CYS:HA	2.02	0.41
1:C:55:ALA:HB2	1:C:64:TYR:CD1	2.56	0.41

Continued on next page...

CONFIDENTIAL VALIDATION REPORT

Continued from previous page...

Atom-1	Atom-2	Interatomic distance (Å)	Clash overlap (Å)
1:G:298:VAL:HG22	1:H:324:ILE:HB	2.03	0.41
1:A:54:ILE:N	1:A:54:ILE:HD12	2.36	0.41
1:H:68:ILE:HD13	1:H:278:MET:HB3	2.03	0.41
1:A:130:SER:O	1:A:133:THR:HG22	2.22	0.40
1:B:189:VAL:HG12	1:G:196:ASP:HB2	2.04	0.40
1:B:87:PHE:O	1:B:125:SER:HA	2.21	0.40
1:D:94:LYS:NZ	3:D:522:HOH:O	2.51	0.40

All (1) symmetry-related close contacts are listed below. The label for Atom-2 includes the symmetry operator and encoded unit-cell translations to be applied.

Atom-1	Atom-2	Interatomic distance (Å)	Clash overlap (Å)
3:A:634:HOH:O	3:C:747:HOH:O[1_556]	2.16	0.04

5.3 Torsion angles ⓘ

5.3.1 Protein backbone ⓘ

In the following table, the Percentiles column shows the percent Ramachandran outliers of the chain as a percentile score with respect to all X-ray entries followed by that with respect to entries of similar resolution.

The Analysed column shows the number of residues for which the backbone conformation was analysed, and the total number of residues.

Mol	Chain	Analysed	Favoured	Allowed	Outliers	Percentiles	
1	A	322/331 (97%)	314 (98%)	7 (2%)	1 (0%)	41	19
1	B	322/331 (97%)	314 (98%)	8 (2%)	0	100	100
1	C	327/331 (99%)	321 (98%)	6 (2%)	0	100	100
1	D	317/331 (96%)	310 (98%)	7 (2%)	0	100	100
1	E	320/331 (97%)	310 (97%)	9 (3%)	1 (0%)	41	19
1	F	324/331 (98%)	317 (98%)	6 (2%)	1 (0%)	41	19
1	G	317/331 (96%)	308 (97%)	7 (2%)	2 (1%)	25	7
1	H	317/331 (96%)	305 (96%)	11 (4%)	1 (0%)	41	19
All	All	2566/2648 (97%)	2499 (97%)	61 (2%)	6 (0%)	47	23

All (6) Ramachandran outliers are listed below:

CONFIDENTIAL VALIDATION REPORT

Mol	Chain	Res	Type
1	E	10	LYS
1	F	10	LYS
1	G	10	LYS
1	G	307	SER
1	H	10	LYS
1	A	10	LYS

5.3.2 Protein sidechains (i)

In the following table, the Percentiles column shows the percent sidechain outliers of the chain as a percentile score with respect to all X-ray entries followed by that with respect to entries of similar resolution.

The Analysed column shows the number of residues for which the sidechain conformation was analysed, and the total number of residues.

Mol	Chain	Analysed	Rotameric	Outliers	Percentiles	
1	A	275/291 (94%)	270 (98%)	5 (2%)	59	31
1	B	277/291 (95%)	274 (99%)	3 (1%)	73	53
1	C	277/291 (95%)	272 (98%)	5 (2%)	59	31
1	D	274/291 (94%)	272 (99%)	2 (1%)	84	69
1	E	276/291 (95%)	271 (98%)	5 (2%)	59	31
1	F	277/291 (95%)	274 (99%)	3 (1%)	73	53
1	G	274/291 (94%)	268 (98%)	6 (2%)	52	23
1	H	270/291 (93%)	267 (99%)	3 (1%)	73	53
All	All	2200/2328 (94%)	2168 (98%)	32 (2%)	65	37

All (32) residues with a non-rotameric sidechain are listed below:

Mol	Chain	Res	Type
1	A	16	ARG
1	A	80	PHE
1	A	132	ASN
1	A	139	HIS
1	A	307	SER
1	B	16	ARG
1	B	80	PHE
1	B	139	HIS
1	C	16	ARG
1	C	80	PHE

Continued on next page...

CONFIDENTIAL VALIDATION REPORT

Continued from previous page...

Mol	Chain	Res	Type
1	C	139	HIS
1	C	182	LEU
1	C	240	ASP
1	D	16	ARG
1	D	139	HIS
1	E	16	ARG
1	E	80	PHE
1	E	139	HIS
1	E	267	ASN
1	E	308	SER
1	F	16	ARG
1	F	80	PHE
1	F	139	HIS
1	G	16	ARG
1	G	18	LEU
1	G	20	TYR
1	G	80	PHE
1	G	139	HIS
1	G	308	SER
1	H	16	ARG
1	H	18	LEU
1	H	139	HIS

Sometimes sidechains can be flipped to improve hydrogen bonding and reduce clashes. All (3) such sidechains are listed below:

Mol	Chain	Res	Type
1	G	59	ASN
1	G	83	GLN
1	H	59	ASN

5.3.3 RNA (i)

There are no RNA molecules in this entry.

5.4 Non-standard residues in protein, DNA, RNA chains (i)

There are no non-standard protein/DNA/RNA residues in this entry.

CONFIDENTIAL VALIDATION REPORT

5.5 Carbohydrates (i)

There are no monosaccharides in this entry.

5.6 Ligand geometry (i)

Of 4 ligands modelled in this entry, 4 are monoatomic - leaving 0 for Mogul analysis.

There are no bond length outliers.

There are no bond angle outliers.

There are no chirality outliers.

There are no torsion outliers.

There are no ring outliers.

No monomer is involved in short contacts.

5.7 Other polymers (i)

There are no such residues in this entry.

5.8 Polymer linkage issues (i)

There are no chain breaks in this entry.

CONFIDENTIAL VALIDATION REPORT

6 Fit of model and data (i)

6.1 Protein, DNA and RNA chains (i)

In the following table, the column labelled '#RSRZ> 2' contains the number (and percentage) of RSRZ outliers, followed by percent RSRZ outliers for the chain as percentile scores relative to all X-ray entries and entries of similar resolution. The OWAB column contains the minimum, median, 95th percentile and maximum values of the occupancy-weighted average B-factor per residue. The column labelled 'Q< 0.9' lists the number of (and percentage) of residues with an average occupancy less than 0.9.

Mol	Chain	Analysed	<RSRZ>	#RSRZ>2			OWAB(Å^2)	Q<0.9
1	A	324/331 (97%)	0.28	23 (7%)	16	18	21, 28, 40, 49	0
1	B	324/331 (97%)	0.40	34 (10%)	6	6	22, 29, 41, 52	0
1	C	329/331 (99%)	0.30	14 (4%)	35	40	22, 30, 41, 48	0
1	D	321/331 (96%)	0.33	16 (4%)	28	33	24, 31, 41, 55	0
1	E	324/331 (97%)	0.61	38 (11%)	4	4	24, 33, 47, 67	0
1	F	326/331 (98%)	0.37	23 (7%)	16	18	24, 32, 43, 55	0
1	G	321/331 (96%)	0.67	44 (13%)	3	2	24, 33, 46, 57	0
1	H	321/331 (96%)	0.52	32 (9%)	7	8	26, 34, 45, 56	0
All	All	2590/2648 (97%)	0.43	224 (8%)	10	11	21, 31, 44, 67	0

All (224) RSRZ outliers are listed below:

Mol	Chain	Res	Type	RSRZ
1	G	22	ILE	7.2
1	E	327	HIS	5.8
1	H	129	PHE	5.8
1	E	22	ILE	5.7
1	A	24	TYR	5.3
1	E	129	PHE	5.1
1	E	24	TYR	4.9
1	E	306	SER	4.6
1	G	305	LEU	4.5
1	D	129	PHE	4.3
1	E	57	VAL	4.3
1	G	59	ASN	4.2
1	B	14	PHE	4.1
1	F	327	HIS	4.1
1	G	92	ASP	4.0
1	E	326	HIS	4.0

Continued on next page...

CONFIDENTIAL VALIDATION REPORT

Continued from previous page...

Mol	Chain	Res	Type	RSRZ
1	B	264	VAL	3.9
1	B	75	VAL	3.9
1	F	326	HIS	3.9
1	H	24	TYR	3.9
1	G	266	PRO	3.9
1	G	128	SER	3.9
1	E	23	ALA	3.9
1	E	134	PRO	3.9
1	E	75	VAL	3.8
1	D	22	ILE	3.7
1	G	267	ASN	3.7
1	E	90	VAL	3.7
1	A	22	ILE	3.7
1	E	131	LYS	3.6
1	A	90	VAL	3.6
1	G	90	VAL	3.6
1	E	267	ASN	3.6
1	H	22	ILE	3.6
1	G	61	THR	3.5
1	G	24	TYR	3.5
1	B	169	LEU	3.5
1	H	306	SER	3.4
1	H	305	LEU	3.4
1	B	22	ILE	3.3
1	G	242	VAL	3.3
1	A	102	ILE	3.3
1	G	134	PRO	3.3
1	H	134	PRO	3.3
1	D	255	VAL	3.3
1	B	76	ALA	3.3
1	C	328	HIS	3.3
1	C	330	HIS	3.2
1	E	305	LEU	3.2
1	G	311	ILE	3.2
1	G	308	SER	3.2
1	H	75	VAL	3.2
1	C	65	CYS	3.2
1	B	102	ILE	3.2
1	G	268	ILE	3.2
1	F	65	CYS	3.2
1	H	43	PRO	3.1
1	E	309	ASP	3.1

Continued on next page...

CONFIDENTIAL VALIDATION REPORT

Continued from previous page...

Mol	Chain	Res	Type	RSRZ
1	A	228	VAL	3.1
1	E	311	ILE	3.1
1	B	141	LEU	3.0
1	G	304	ASP	3.0
1	H	323	PHE	3.0
1	A	75	VAL	3.0
1	A	67	ALA	3.0
1	G	58	ALA	3.0
1	H	47	SER	3.0
1	D	242	VAL	3.0
1	A	141	LEU	3.0
1	G	87	PHE	3.0
1	G	309	ASP	3.0
1	E	60	ASN	3.0
1	A	101	PHE	2.9
1	G	76	ALA	2.9
1	D	266	PRO	2.9
1	C	22	ILE	2.9
1	E	268	ILE	2.9
1	B	90	VAL	2.9
1	A	14	PHE	2.9
1	B	67	ALA	2.9
1	G	57	VAL	2.9
1	G	2	CYS	2.8
1	H	267	ASN	2.8
1	F	140	TRP	2.8
1	F	46	LYS	2.8
1	B	140	TRP	2.8
1	B	101	PHE	2.8
1	C	133	THR	2.7
1	A	169	LEU	2.7
1	A	54	ILE	2.7
1	B	54	ILE	2.7
1	C	266	PRO	2.7
1	G	88	PRO	2.7
1	C	140	TRP	2.7
1	E	264	VAL	2.7
1	H	255	VAL	2.7
1	D	24	TYR	2.7
1	E	59	ASN	2.7
1	C	75	VAL	2.7
1	E	266	PRO	2.7

Continued on next page...

CONFIDENTIAL VALIDATION REPORT

Continued from previous page...

Mol	Chain	Res	Type	RSRZ
1	G	310	LEU	2.7
1	F	3	THR	2.6
1	C	228	VAL	2.6
1	D	267	ASN	2.6
1	D	134	PRO	2.6
1	H	2	CYS	2.6
1	E	135	ALA	2.6
1	B	265	GLY	2.6
1	F	24	TYR	2.6
1	G	74	GLY	2.6
1	E	130	SER	2.6
1	H	50	ALA	2.6
1	H	228	VAL	2.6
1	B	24	TYR	2.5
1	B	215	LEU	2.5
1	B	252	LEU	2.5
1	G	68	ILE	2.5
1	A	140	TRP	2.5
1	B	2	CYS	2.5
1	A	312	VAL	2.5
1	B	228	VAL	2.5
1	F	238	ASP	2.5
1	B	3	THR	2.5
1	B	5	ILE	2.5
1	D	44	ALA	2.4
1	E	269	PHE	2.4
1	E	2	CYS	2.4
1	H	304	ASP	2.4
1	G	297	ALA	2.4
1	G	141	LEU	2.4
1	H	252	LEU	2.4
1	E	87	PHE	2.4
1	E	316	PHE	2.4
1	F	101	PHE	2.4
1	F	225	PHE	2.4
1	D	2	CYS	2.4
1	B	74	GLY	2.4
1	H	42	LEU	2.4
1	B	226	VAL	2.4
1	E	67	ALA	2.4
1	F	67	ALA	2.4
1	G	96	ILE	2.4

Continued on next page...

CONFIDENTIAL VALIDATION REPORT

Continued from previous page...

Mol	Chain	Res	Type	RSRZ
1	H	309	ASP	2.4
1	F	141	LEU	2.4
1	E	127	VAL	2.3
1	G	89	VAL	2.3
1	B	133	THR	2.3
1	B	15	GLY	2.3
1	F	22	ILE	2.3
1	C	141	LEU	2.3
1	A	76	ALA	2.3
1	E	61	THR	2.3
1	H	34	ASN	2.3
1	F	14	PHE	2.3
1	A	5	ILE	2.3
1	G	32	PRO	2.3
1	G	169	LEU	2.3
1	G	325	ASN	2.3
1	A	77	GLY	2.3
1	D	325	ASN	2.3
1	G	3	THR	2.3
1	C	78	LEU	2.3
1	B	266	PRO	2.2
1	H	23	ALA	2.2
1	D	194	ASN	2.2
1	G	140	TRP	2.2
1	B	142	VAL	2.2
1	G	40	ALA	2.2
1	G	129	PHE	2.2
1	B	277	CYS	2.2
1	H	302	LYS	2.2
1	D	216	PRO	2.2
1	C	215	LEU	2.2
1	G	5	ILE	2.2
1	E	255	VAL	2.2
1	F	226	VAL	2.2
1	H	245	VAL	2.2
1	H	312	VAL	2.2
1	A	53	GLY	2.2
1	B	249	PHE	2.2
1	B	106	LEU	2.2
1	F	252	LEU	2.2
1	H	311	ILE	2.2
1	H	216	PRO	2.2

Continued on next page...

CONFIDENTIAL VALIDATION REPORT

Continued from previous page...

Mol	Chain	Res	Type	RSRZ
1	E	36	GLU	2.2
1	E	84	GLY	2.1
1	D	321	ILE	2.1
1	G	226	VAL	2.1
1	F	306	SER	2.1
1	A	92	ASP	2.1
1	D	3	THR	2.1
1	G	23	ALA	2.1
1	G	135	ALA	2.1
1	H	301	ASN	2.1
1	B	216	PRO	2.1
1	E	102	ILE	2.1
1	F	5	ILE	2.1
1	B	64	TYR	2.1
1	H	127	VAL	2.1
1	E	140	TRP	2.1
1	H	130	SER	2.1
1	D	323	PHE	2.1
1	F	77	GLY	2.1
1	G	60	ASN	2.1
1	A	52	ILE	2.1
1	F	10	LYS	2.1
1	E	89	VAL	2.1
1	E	93	LYS	2.1
1	H	212	THR	2.1
1	H	249	PHE	2.1
1	F	44	ALA	2.1
1	F	223	SER	2.0
1	C	185	TYR	2.0
1	B	255	VAL	2.0
1	G	75	VAL	2.0
1	G	127	VAL	2.0
1	A	74	GLY	2.0
1	F	170	THR	2.0
1	A	59	ASN	2.0
1	H	169	LEU	2.0
1	A	142	VAL	2.0
1	B	105	ILE	2.0
1	C	3	THR	2.0
1	E	128	SER	2.0

CONFIDENTIAL VALIDATION REPORT

6.2 Non-standard residues in protein, DNA, RNA chains (i)

There are no non-standard protein/DNA/RNA residues in this entry.

6.3 Carbohydrates (i)

There are no monosaccharides in this entry.

6.4 Ligands (i)

In the following table, the Atoms column lists the number of modelled atoms in the group and the number defined in the chemical component dictionary. The B-factors column lists the minimum, median, 95th percentile and maximum values of B factors of atoms in the group. The column labelled 'Q< 0.9' lists the number of atoms with occupancy less than 0.9.

Mol	Type	Chain	Res	Atoms	RSCC	RSR	B-factors(Å^2)	Q<0.9
2	MG	H	401	1/1	0.92	0.07	37,37,37,37	0
2	MG	C	401	1/1	0.94	0.05	34,34,34,34	0
2	MG	F	401	1/1	0.96	0.04	34,34,34,34	0
2	MG	D	401	1/1	0.97	0.13	38,38,38,38	0

6.5 Other polymers (i)

There are no such residues in this entry.

CONFIDENTIAL VALIDATION REPORT
