# Supplementary material for: Bile salt hydrolases shape the bile acid landscape and restrict Clostridioides difficile growth in the murine gut
Source: Nat Microbiol. 2023 Mar 13;8(4):611–28. doi: 10.1038/s41564-023-01337-7 (PMC10066039; doi:10.1038/s41564-023-01337-7)
Supplement: Supplementary file 10 — 7SVI PDB validation report. [file 41564_2023_1337_MOESM10_ESM.pdf]

Full wwPDB X-ray Structure Validation Report (i)

Jan 5, 2022 – 04:49 PM EST

PDB ID	:	7SVI
Title	:	Bile Salt Hydrolase C from Lactobacillus johnsonii
Deposited on	:	2021-11-19
Resolution	:	1.45 Å(reported)

This is a Full wwPDB X-ray Structure Validation Report.

This report is produced by the wwPDB biocuration pipeline after annotation of the structure.

We welcome your comments at *validation@mail.wwpdb.org*
A user guide is available at
https://www.wwpdb.org/validation/2017/XrayValidationReportHelp
with specific help available everywhere you see the (i) symbol.

CONFIDENTIAL VALIDATION REPORT

The following versions of software and data (see references (i)) were used in the production of this report:

MolProbity	:	4.02b-467
Xtriage (Phenix)	:	1.13
EDS	:	2.25
Percentile statistics	:	20191225.v01 (using entries in the PDB archive December 25th 2019)
Refmac	:	5.8.0158
CCP4	:	7.0.044 (Gargrove)
Ideal geometry (proteins)	:	Engh & Huber (2001)
Ideal geometry (DNA, RNA)	:	Parkinson et al. (1996)
Validation Pipeline (wwPDB-VP)	:	2.25

1 Overall quality at a glance (i)

The following experimental techniques were used to determine the structure:
X-RAY DIFFRACTION

The reported resolution of this entry is 1.45 Å.

Percentile scores (ranging between 0-100) for global validation metrics of the entry are shown in the following graphic. The table shows the number of entries on which the scores are based.

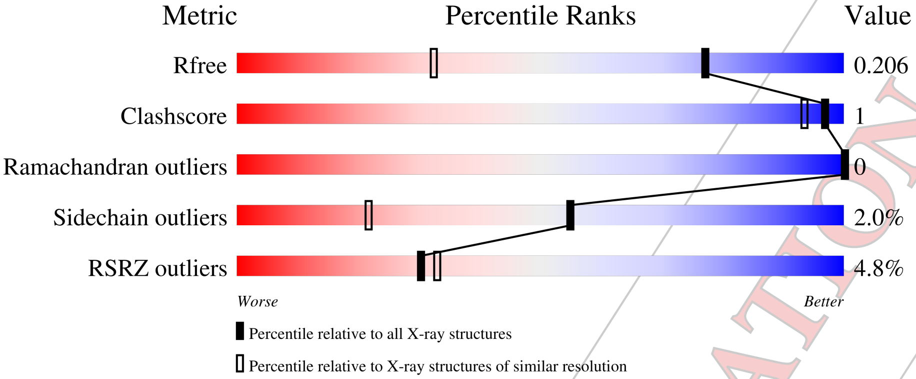

Metric	Whole archive (#Entries)	Similar resolution (#Entries, resolution range(Å))
R_{free}	130704	1156 (1.46-1.46)
Clashscore	141614	1202 (1.46-1.46)
Ramachandran outliers	138981	1178 (1.46-1.46)
Sidechain outliers	138945	1178 (1.46-1.46)
RSRZ outliers	127900	1139 (1.46-1.46)

The table below summarises the geometric issues observed across the polymeric chains and their fit to the electron density. The red, orange, yellow and green segments of the lower bar indicate the fraction of residues that contain outliers for >=3, 2, 1 and 0 types of geometric quality criteria respectively. A grey segment represents the fraction of residues that are not modelled. The numeric value for each fraction is indicated below the corresponding segment, with a dot representing fractions <=5% The upper red bar (where present) indicates the fraction of residues that have poor fit to the electron density. The numeric value is given above the bar.

Mol	Chain	Length	Quality of chain
1	A	322	4% / 97% •
1	B	322	2% / 97% •
1	C	322	3% / 96% •
1	D	322	10% / 94% • • •

CONFIDENTIAL VALIDATION REPORT

2 Entry composition (i)

There are 2 unique types of molecules in this entry. The entry contains 11399 atoms, of which 0 are hydrogens and 0 are deuteriums.

In the tables below, the ZeroOcc column contains the number of atoms modelled with zero occupancy, the AltConf column contains the number of residues with at least one atom in alternate conformation and the Trace column contains the number of residues modelled with at most 2 atoms.

- Molecule 1 is a protein called Choloylglycine hydrolase.

Mol	Chain	Residues	Atoms					ZeroOcc	AltConf	Trace
1	A	321	Total 2507	C 1606	N 412	O 480	S 9	0	0	0
1	B	321	Total 2500	C 1601	N 411	O 479	S 9	0	0	0
1	C	321	Total 2494	C 1596	N 410	O 479	S 9	0	0	0
1	D	317	Total 2447	C 1569	N 395	O 474	S 9	0	0	0

- Molecule 2 is water.

Mol	Chain	Residues	Atoms		ZeroOcc	AltConf
2	A	387	Total 387	O 387	0	0
2	B	403	Total 403	O 403	0	0
2	C	375	Total 375	O 375	0	0
2	D	286	Total 286	O 286	0	0

CONFIDENTIAL VALIDATION REPORT

3 Residue-property plots (i)

These plots are drawn for all protein, RNA, DNA and oligosaccharide chains in the entry. The first graphic for a chain summarises the proportions of the various outlier classes displayed in the second graphic. The second graphic shows the sequence view annotated by issues in geometry and electron density. Residues are color-coded according to the number of geometric quality criteria for which they contain at least one outlier: green = 0, yellow = 1, orange = 2 and red = 3 or more. A red dot above a residue indicates a poor fit to the electron density (RSRZ > 2). Stretches of 2 or more consecutive residues without any outlier are shown as a green connector. Residues present in the sample, but not in the model, are shown in grey.

- Molecule 1: Choloylglycine hydrolase

Chain A:

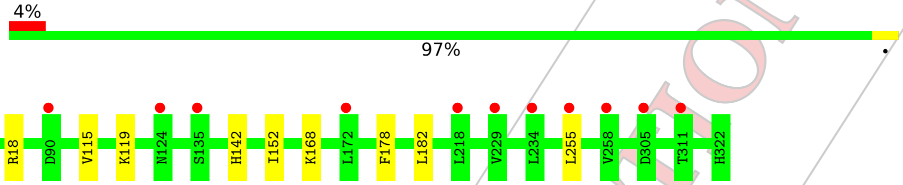

- Molecule 1: Choloylglycine hydrolase

Chain B:

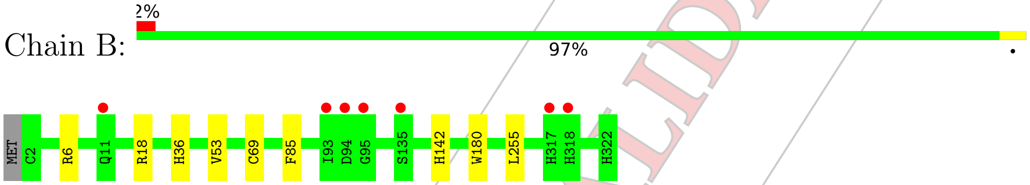

- Molecule 1: Choloylglycine hydrolase

Chain C:

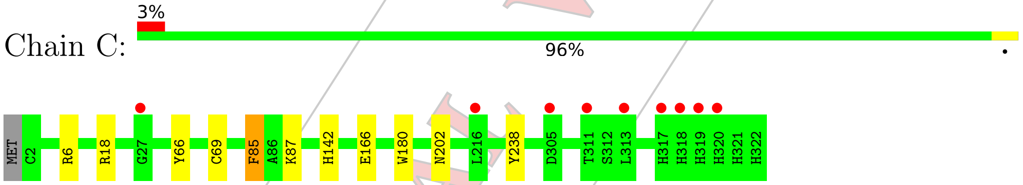

- Molecule 1: Choloylglycine hydrolase

Chain D:

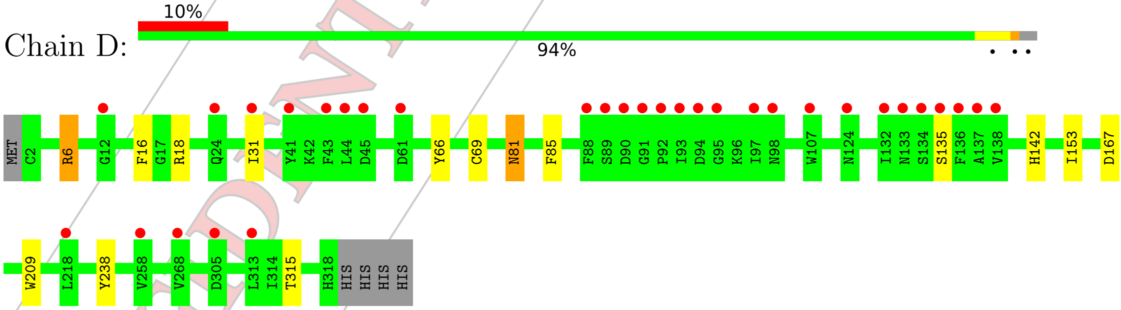

CONFIDENTIAL VALIDATION REPORT

4 Data and refinement statistics (i)

Property	Value	Source
Space group	P 2 21 21	Depositor
Cell constants<br>a, b, c, α, β, γ	58.07Å 80.88Å 324.54Å<br>90.00° 90.00° 90.00°	Depositor
Resolution (Å)	45.29 – 1.45<br>45.29 – 1.45	Depositor<br>EDS
% Data completeness<br>(in resolution range)	97.3 (45.29-1.45)<br>97.3 (45.29-1.45)	Depositor<br>EDS
R_{merge}	0.05	Depositor
R_{sym}	(Not available)	Depositor
$< I/\sigma(I) >$ [1]	1.07 (at 1.45Å)	Xtriage
Refinement program	PHENIX 1.17.1_3660, PHENIX 1.17.1_3660	Depositor
R, R_{free}	0.183 , 0.206<br>0.183 , 0.206	Depositor<br>DCC
R_{free} test set	2000 reflections (0.76%)	wwPDB-VP
Wilson B-factor (Å^2)	22.6	Xtriage
Anisotropy	0.350	Xtriage
Bulk solvent k_{sol}(e/Å^3), B_{sol}(Å^2)	0.33 , 38.0	EDS
L-test for twinning[2]	$< \|L\| > = 0.49$, $< L^2 > = 0.32$	Xtriage
Estimated twinning fraction	No twinning to report.	Xtriage
F_o,F_c correlation	0.97	EDS
Total number of atoms	11399	wwPDB-VP
Average B, all atoms (Å^2)	27.0	wwPDB-VP

Xtriage's analysis on translational NCS is as follows: *The largest off-origin peak in the Patterson function is 3.21% of the height of the origin peak. No significant pseudotranslation is detected.*

[1] Intensities estimated from amplitudes.

[2] Theoretical values of $< |L| >$, $< L^2 >$ for acentric reflections are 0.5, 0.333 respectively for untwinned datasets, and 0.375, 0.2 for perfectly twinned datasets.

CONFIDENTIAL VALIDATION REPORT

5 Model quality (i)

5.1 Standard geometry (i)

The Z score for a bond length (or angle) is the number of standard deviations the observed value is removed from the expected value. A bond length (or angle) with $|Z| > 5$ is considered an outlier worth inspection. RMSZ is the root-mean-square of all Z scores of the bond lengths (or angles).

Mol	Chain	Bond lengths		Bond angles	
		RMSZ	#$|Z|$ >5	RMSZ	#$|Z|$ >5
1	A	0.35	0/2576	0.58	0/3506
1	B	0.33	0/2569	0.56	0/3499
1	C	0.34	0/2563	0.55	0/3493
1	D	0.33	0/2511	0.54	0/3422
All	All	0.34	0/10219	0.56	0/13920

There are no bond length outliers.

There are no bond angle outliers.

There are no chirality outliers.

There are no planarity outliers.

5.2 Too-close contacts (i)

In the following table, the Non-H and H(model) columns list the number of non-hydrogen atoms and hydrogen atoms in the chain respectively. The H(added) column lists the number of hydrogen atoms added and optimized by MolProbity. The Clashes column lists the number of clashes within the asymmetric unit, whereas Symm-Clashes lists symmetry-related clashes.

Mol	Chain	Non-H	H(model)	H(added)	Clashes	Symm-Clashes
1	A	2507	0	2385	6	0
1	B	2500	0	2367	3	0
1	C	2494	0	2352	6	0
1	D	2447	0	2317	7	1
2	A	387	0	0	0	0
2	B	403	0	0	0	0
2	C	375	0	0	1	0
2	D	286	0	0	0	0
All	All	11399	0	9421	18	1

The all-atom clashscore is defined as the number of clashes found per 1000 atoms (including hydrogen atoms). The all-atom clashscore for this structure is 1.

CONFIDENTIAL VALIDATION REPORT

All (18) close contacts within the same asymmetric unit are listed below, sorted by their clash magnitude.

Atom-1	Atom-2	Interatomic distance (Å)	Clash overlap (Å)
1:C:166:GLU:OE1	2:C:401:HOH:O	2.14	0.66
1:D:31:ILE:HG12	1:D:315:THR:HG22	1.83	0.61
1:A:119:LYS:HD3	1:A:152:ILE:HG21	1.85	0.58
1:A:119:LYS:HG2	1:A:152:ILE:HD13	1.88	0.56
1:C:66:TYR:CG	1:C:69:CYS:HB3	2.52	0.45
1:A:115:VAL:O	1:A:119:LYS:HG3	2.17	0.44
1:D:153:ILE:HG12	1:D:167:ASP:HB2	2.00	0.44
1:D:66:TYR:CG	1:D:69:CYS:HB3	2.53	0.44
1:C:85:PHE:CE2	1:D:209:TRP:CZ3	3.06	0.43
1:B:180:TRP:CD1	1:C:180:TRP:CD1	3.08	0.42
1:B:255:LEU:HD23	1:B:255:LEU:HA	1.89	0.41
1:C:85:PHE:HE2	1:D:209:TRP:CZ3	2.39	0.41
1:A:168:LYS:HE3	1:C:202:ASN:O	2.20	0.41
1:A:178:PHE:CE2	1:A:182:LEU:HD11	2.55	0.41
1:D:6:ARG:HB3	1:D:16:PHE:CD1	2.56	0.41
1:B:53:VAL:CG1	1:B:69:CYS:HB2	2.51	0.41
1:A:255:LEU:HD23	1:A:255:LEU:HA	1.91	0.40
1:D:81:ASN:HD22	1:D:81:ASN:HA	1.75	0.40

All (1) symmetry-related close contacts are listed below. The label for Atom-2 includes the symmetry operator and encoded unit-cell translations to be applied.

Atom-1	Atom-2	Interatomic distance (Å)	Clash overlap (Å)
1:D:315:THR:OG1	1:D:315:THR:OG1[2_566]	2.05	0.15

5.3 Torsion angles (i)

5.3.1 Protein backbone (i)

In the following table, the Percentiles column shows the percent Ramachandran outliers of the chain as a percentile score with respect to all X-ray entries followed by that with respect to entries of similar resolution.

The Analysed column shows the number of residues for which the backbone conformation was analysed, and the total number of residues.

Mol	Chain	Analysed	Favoured	Allowed	Outliers	Percentiles	
1	A	319/322 (99%)	310 (97%)	9 (3%)	0	100	100

Continued on next page...

CONFIDENTIAL VALIDATION REPORT

Continued from previous page...

Mol	Chain	Analysed	Favoured	Allowed	Outliers	Percentiles	
1	B	319/322 (99%)	310 (97%)	9 (3%)	0	100	100
1	C	319/322 (99%)	310 (97%)	9 (3%)	0	100	100
1	D	315/322 (98%)	306 (97%)	9 (3%)	0	100	100
All	All	1272/1288 (99%)	1236 (97%)	36 (3%)	0	100	100

There are no Ramachandran outliers to report.

5.3.2 Protein sidechains (i)

In the following table, the Percentiles column shows the percent sidechain outliers of the chain as a percentile score with respect to all X-ray entries followed by that with respect to entries of similar resolution.

The Analysed column shows the number of residues for which the sidechain conformation was analysed, and the total number of residues.

Mol	Chain	Analysed	Rotameric	Outliers	Percentiles	
1	A	268/272 (98%)	265 (99%)	3 (1%)	73	48
1	B	266/272 (98%)	261 (98%)	5 (2%)	57	23
1	C	265/272 (97%)	259 (98%)	6 (2%)	50	17
1	D	259/272 (95%)	252 (97%)	7 (3%)	44	12
All	All	1058/1088 (97%)	1037 (98%)	21 (2%)	55	22

All (21) residues with a non-rotameric sidechain are listed below:

Mol	Chain	Res	Type
1	A	6	ARG
1	A	18	ARG
1	A	142	HIS
1	B	6	ARG
1	B	18	ARG
1	B	36	HIS
1	B	85	PHE
1	B	142	HIS
1	C	6	ARG
1	C	18	ARG
1	C	85	PHE
1	C	87	LYS
1	C	142	HIS

Continued on next page...

CONFIDENTIAL VALIDATION REPORT

Continued from previous page...

Mol	Chain	Res	Type
1	C	238	TYR
1	D	6	ARG
1	D	18	ARG
1	D	81	ASN
1	D	85	PHE
1	D	135	SER
1	D	142	HIS
1	D	238	TYR

Sometimes sidechains can be flipped to improve hydrogen bonding and reduce clashes. All (3) such sidechains are listed below:

Mol	Chain	Res	Type
1	A	114	HIS
1	A	124	ASN
1	D	81	ASN

5.3.3 RNA (i)

There are no RNA molecules in this entry.

5.4 Non-standard residues in protein, DNA, RNA chains (i)

There are no non-standard protein/DNA/RNA residues in this entry.

5.5 Carbohydrates (i)

There are no monosaccharides in this entry.

5.6 Ligand geometry (i)

There are no ligands in this entry.

5.7 Other polymers (i)

There are no such residues in this entry.

CONFIDENTIAL VALIDATION REPORT

5.8 Polymer linkage issues (i)

There are no chain breaks in this entry.

CONFIDENTIAL VALIDATION REPORT

6 Fit of model and data ⓘ

6.1 Protein, DNA and RNA chains ⓘ

In the following table, the column labelled '#RSRZ> 2' contains the number (and percentage) of RSRZ outliers, followed by percent RSRZ outliers for the chain as percentile scores relative to all X-ray entries and entries of similar resolution. The OWAB column contains the minimum, median, 95th percentile and maximum values of the occupancy-weighted average B-factor per residue. The column labelled 'Q< 0.9' lists the number of (and percentage) of residues with an average occupancy less than 0.9.

Mol	Chain	Analysed	<RSRZ>	#RSRZ>2			OWAB(Å^2)	Q<0.9
1	A	321/322 (99%)	0.42	13 (4%)	38	40	18, 23, 33, 43	0
1	B	321/322 (99%)	0.35	7 (2%)	62	65	18, 24, 35, 44	0
1	C	321/322 (99%)	0.44	9 (2%)	53	55	18, 25, 36, 47	0
1	D	317/322 (98%)	0.78	32 (10%)	7	8	19, 28, 44, 53	0
All	All	1280/1288 (99%)	0.49	61 (4%)	30	33	18, 25, 38, 53	0

All (61) RSRZ outliers are listed below:

Mol	Chain	Res	Type	RSRZ
1	D	93	ILE	5.6
1	D	136	PHE	5.3
1	D	134	SER	5.1
1	C	311	THR	5.1
1	D	94	ASP	4.5
1	D	135	SER	4.5
1	D	97	ILE	4.2
1	D	95	GLY	4.2
1	D	89	SER	3.9
1	D	91	GLY	3.9
1	B	318	HIS	3.8
1	D	92	PRO	3.7
1	B	11	GLN	3.5
1	B	93	ILE	3.4
1	D	90	ASP	3.3
1	C	317	HIS	3.2
1	B	317	HIS	3.2
1	D	124	ASN	3.1
1	D	132	ILE	3.0
1	C	216	LEU	2.9
1	D	313	LEU	2.9

Continued on next page...

CONFIDENTIAL VALIDATION REPORT

Continued from previous page...

Mol	Chain	Res	Type	RSRZ
1	C	319	HIS	2.9
1	D	43	PHE	2.7
1	A	258	VAL	2.7
1	D	41	TYR	2.7
1	B	94	ASP	2.7
1	C	318	HIS	2.6
1	A	229	VAL	2.6
1	C	27	GLY	2.6
1	D	305	ASP	2.6
1	D	218	LEU	2.5
1	D	98	ASN	2.5
1	A	90	ASP	2.4
1	A	124	ASN	2.4
1	C	320	HIS	2.4
1	A	172	LEU	2.4
1	B	135	SER	2.4
1	A	255	LEU	2.4
1	D	268	VAL	2.3
1	A	234	LEU	2.3
1	D	45	ASP	2.3
1	B	95	GLY	2.3
1	D	107	TRP	2.3
1	C	313	LEU	2.3
1	D	88	PHE	2.2
1	D	44	LEU	2.2
1	D	138	VAL	2.2
1	D	258	VAL	2.2
1	A	135	SER	2.2
1	D	12	GLY	2.2
1	D	137	ALA	2.2
1	D	61	ASP	2.2
1	D	133	ASN	2.2
1	A	311	THR	2.1
1	C	305	ASP	2.1
1	A	218	LEU	2.1
1	D	31	ILE	2.1
1	A	2	CYS	2.1
1	D	24	GLN	2.1
1	A	305	ASP	2.1
1	A	10	GLU	2.0

CONFIDENTIAL VALIDATION REPORT

6.2 Non-standard residues in protein, DNA, RNA chains (i)

There are no non-standard protein/DNA/RNA residues in this entry.

6.3 Carbohydrates (i)

There are no monosaccharides in this entry.

6.4 Ligands (i)

There are no ligands in this entry.

6.5 Other polymers (i)

There are no such residues in this entry.

CONFIDENTIAL VALIDATION REPORT
