# Supplementary material for: Bile salt hydrolases shape the bile acid landscape and restrict Clostridioides difficile growth in the murine gut
Source: Nat Microbiol. 2023 Mar 13;8(4):611–28. doi: 10.1038/s41564-023-01337-7 (PMC10066039; doi:10.1038/s41564-023-01337-7)
Supplement: Supplementary file 11 — 7SVJ PDB validation report. [file 41564_2023_1337_MOESM11_ESM.pdf]

Full wwPDB X-ray Structure Validation Report (i)

Nov 30, 2021 – 01:54 PM EST

PDB ID	:	7SVJ
Title	:	Bile Salt Hydrolase from Lactobacillus ingluviei
Deposited on	:	2021-11-19
Resolution	:	1.43 Å(reported)

This is a Full wwPDB X-ray Structure Validation Report.

This report is produced by the wwPDB biocuration pipeline after annotation of the structure.

We welcome your comments at *validation@mail.wwpdb.org*
A user guide is available at
https://www.wwpdb.org/validation/2017/XrayValidationReportHelp
with specific help available everywhere you see the (i) symbol.

CONFIDENTIAL VALIDATION REPORT

The following versions of software and data (see references (i)) were used in the production of this report:

MolProbity	:	4.02b-467
Mogul	:	1.8.5 (274361), CSD as541be (2020)
Xtriage (Phenix)	:	1.13
EDS	:	2.23.2
Percentile statistics	:	20191225.v01 (using entries in the PDB archive December 25th 2019)
Refmac	:	5.8.0158
CCP4	:	7.0.044 (Gargrove)
Ideal geometry (proteins)	:	Engh & Huber (2001)
Ideal geometry (DNA, RNA)	:	Parkinson et al. (1996)
Validation Pipeline (wwPDB-VP)	:	2.23.2

1 Overall quality at a glance (i)

The following experimental techniques were used to determine the structure:
X-RAY DIFFRACTION

The reported resolution of this entry is 1.43 Å.

Percentile scores (ranging between 0-100) for global validation metrics of the entry are shown in the following graphic. The table shows the number of entries on which the scores are based.

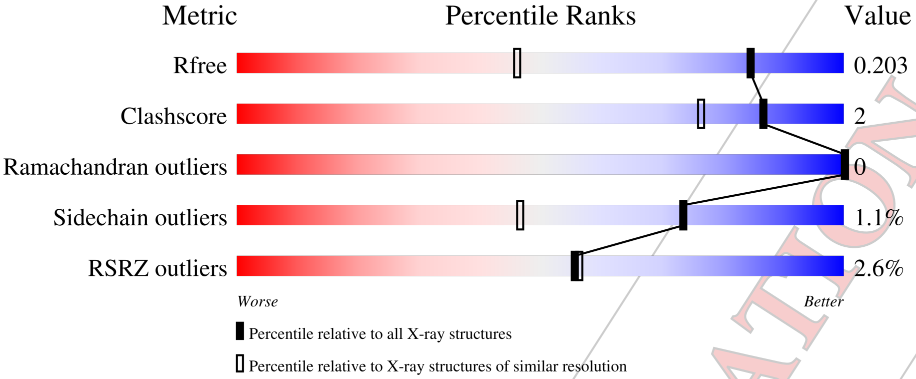

Metric	Whole archive (#Entries)	Similar resolution (#Entries, resolution range(Å))
R_{free}	130704	2021 (1.46-1.42)
Clashscore	141614	2086 (1.46-1.42)
Ramachandran outliers	138981	2047 (1.46-1.42)
Sidechain outliers	138945	2047 (1.46-1.42)
RSRZ outliers	127900	1993 (1.46-1.42)

The table below summarises the geometric issues observed across the polymeric chains and their fit to the electron density. The red, orange, yellow and green segments of the lower bar indicate the fraction of residues that contain outliers for >=3, 2, 1 and 0 types of geometric quality criteria respectively. A grey segment represents the fraction of residues that are not modelled. The numeric value for each fraction is indicated below the corresponding segment, with a dot representing fractions <=5% The upper red bar (where present) indicates the fraction of residues that have poor fit to the electron density. The numeric value is given above the bar.

Mol	Chain	Length	Quality of chain
1	A	333	5% / 92% 6% •
1	B	333	% / 94% • •

CONFIDENTIAL VALIDATION REPORT

2 Entry composition (i)

There are 6 unique types of molecules in this entry. The entry contains 5667 atoms, of which 0 are hydrogens and 0 are deuteriums.

In the tables below, the ZeroOcc column contains the number of atoms modelled with zero occupancy, the AltConf column contains the number of residues with at least one atom in alternate conformation and the Trace column contains the number of residues modelled with at most 2 atoms.

- Molecule 1 is a protein called Choloylglycine hydrolase.

Mol	Chain	Residues	Atoms					ZeroOcc	AltConf	Trace
			Total	C	N	O	S			
1	A	326	2561	1631	430	493	7	0	0	0
1	B	326	2571	1640	428	496	7	0	0	0

There are 12 discrepancies between the modelled and reference sequences:

Chain	Residue	Modelled	Actual	Comment	Reference
A	328	HIS	-	expression tag	UNP A0A0R1U3N2
A	329	HIS	-	expression tag	UNP A0A0R1U3N2
A	330	HIS	-	expression tag	UNP A0A0R1U3N2
A	331	HIS	-	expression tag	UNP A0A0R1U3N2
A	332	HIS	-	expression tag	UNP A0A0R1U3N2
A	333	HIS	-	expression tag	UNP A0A0R1U3N2
B	328	HIS	-	expression tag	UNP A0A0R1U3N2
B	329	HIS	-	expression tag	UNP A0A0R1U3N2
B	330	HIS	-	expression tag	UNP A0A0R1U3N2
B	331	HIS	-	expression tag	UNP A0A0R1U3N2
B	332	HIS	-	expression tag	UNP A0A0R1U3N2
B	333	HIS	-	expression tag	UNP A0A0R1U3N2

- Molecule 2 is TETRAETHYLENE GLYCOL (three-letter code: PG4) (formula: $C_8H_{18}O_5$).

CONFIDENTIAL VALIDATION REPORT

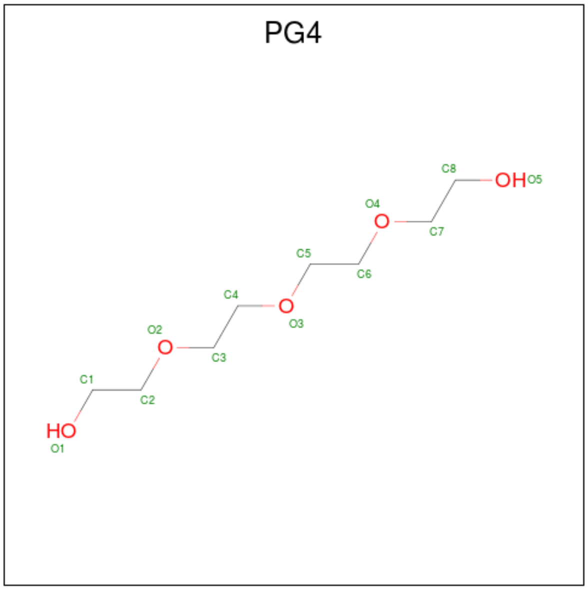

Mol	Chain	Residues	Atoms			ZeroOcc	AltConf
			Total	C	O		
2	A	1	13	8	5	0	0
			Total	C	O		
2	A	1	13	8	5	0	0
			Total	C	O		
2	B	1	13	8	5	0	0

- Molecule 3 is DI(HYDROXYETHYL)ETHER (three-letter code: PEG) (formula: $C_4H_{10}O_3$).

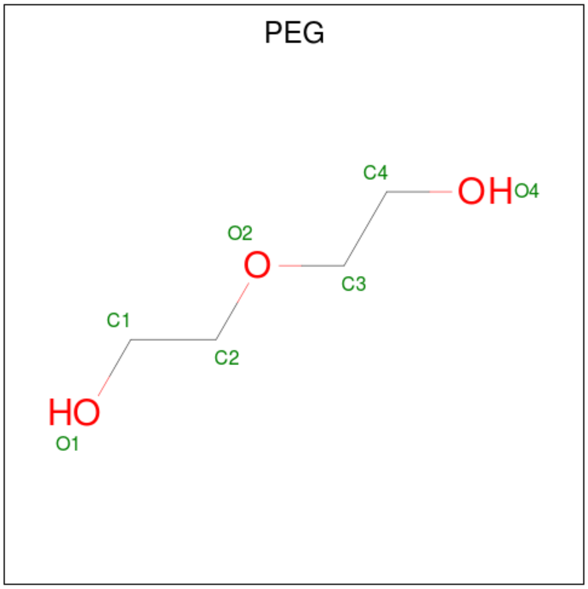

Mol	Chain	Residues	Atoms			ZeroOcc	AltConf
			Total	C	O		
3	B	1	7	4	3	0	0

CONFIDENTIAL VALIDATION REPORT

- Molecule 4 is TRIETHYLENE GLYCOL (three-letter code: PGE) (formula: $C_6H_{14}O_4$).

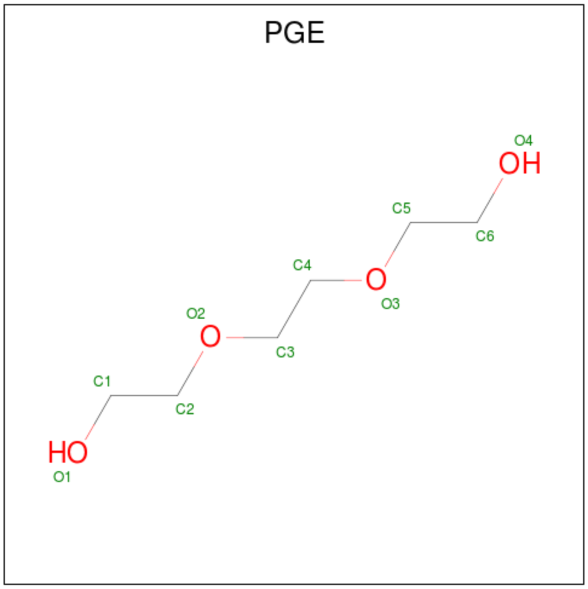

Mol	Chain	Residues	Atoms	ZeroOcc	AltConf
4	B	1	Total C O<br>10 6 4	0	0

- Molecule 5 is CALCIUM ION (three-letter code: CA) (formula: Ca).

Mol	Chain	Residues	Atoms	ZeroOcc	AltConf
5	B	2	Total Ca<br>2 2	0	0

- Molecule 6 is water.

Mol	Chain	Residues	Atoms	ZeroOcc	AltConf
6	A	221	Total O<br>221 221	0	0
6	B	256	Total O<br>256 256	0	0

CONFIDENTIAL VALIDATION REPORT

3 Residue-property plots (i)

These plots are drawn for all protein, RNA, DNA and oligosaccharide chains in the entry. The first graphic for a chain summarises the proportions of the various outlier classes displayed in the second graphic. The second graphic shows the sequence view annotated by issues in geometry and electron density. Residues are color-coded according to the number of geometric quality criteria for which they contain at least one outlier: green = 0, yellow = 1, orange = 2 and red = 3 or more. A red dot above a residue indicates a poor fit to the electron density (RSRZ > 2). Stretches of 2 or more consecutive residues without any outlier are shown as a green connector. Residues present in the sample, but not in the model, are shown in grey.

- Molecule 1: Choloylglycine hydrolase

Chain A:

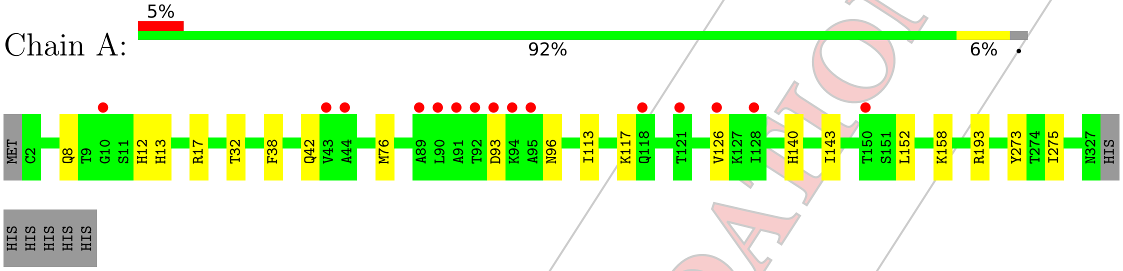

- Molecule 1: Choloylglycine hydrolase

Chain B:

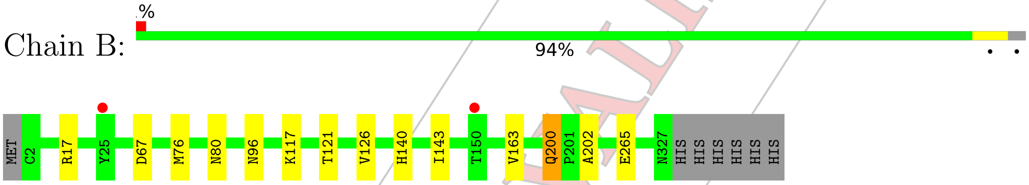

CONFIDENTIAL VALIDATION REPORT

4 Data and refinement statistics (i)

Property	Value	Source
Space group	I 1 2 1	Depositor
Cell constants<br>a, b, c, α, β, γ	90.64Å 43.81Å 155.06Å<br>90.00° 99.36° 90.00°	Depositor
Resolution (Å)	42.12 – 1.43<br>42.12 – 1.43	Depositor<br>EDS
% Data completeness<br>(in resolution range)	97.4 (42.12-1.43)<br>97.5 (42.12-1.43)	Depositor<br>EDS
R_{merge}	0.04	Depositor
R_{sym}	(Not available)	Depositor
$< I/\sigma(I) >$ [1]	1.26 (at 1.43Å)	Xtriage
Refinement program	PHENIX 1.17.1_3660, PHENIX 1.17.1_3660	Depositor
R, R_{free}	0.177 , 0.203<br>0.177 , 0.203	Depositor<br>DCC
R_{free} test set	1999 reflections (1.84%)	wwPDB-VP
Wilson B-factor (Å^2)	18.8	Xtriage
Anisotropy	0.492	Xtriage
Bulk solvent k_{sol}(e/Å^3), B_{sol}(Å^2)	0.38 , 41.4	EDS
L-test for twinning[2]	$< \|L\| > = 0.50$, $< L^2 > = 0.33$	Xtriage
Estimated twinning fraction	No twinning to report.	Xtriage
F_o,F_c correlation	0.98	EDS
Total number of atoms	5667	wwPDB-VP
Average B, all atoms (Å^2)	24.0	wwPDB-VP

Xtriage's analysis on translational NCS is as follows: *The largest off-origin peak in the Patterson function is 6.99% of the height of the origin peak. No significant pseudotranslation is detected.*

[1] Intensities estimated from amplitudes.

[2] Theoretical values of $< |L| >$, $< L^2 >$ for acentric reflections are 0.5, 0.333 respectively for untwinned datasets, and 0.375, 0.2 for perfectly twinned datasets.

CONFIDENTIAL VALIDATION REPORT

5 Model quality (i)

5.1 Standard geometry (i)

Bond lengths and bond angles in the following residue types are not validated in this section: PG4, PGE, PEG, CA

The Z score for a bond length (or angle) is the number of standard deviations the observed value is removed from the expected value. A bond length (or angle) with $|Z| > 5$ is considered an outlier worth inspection. RMSZ is the root-mean-square of all Z scores of the bond lengths (or angles).

Mol	Chain	Bond lengths		Bond angles	
		RMSZ	#|Z| >5	RMSZ	#|Z| >5
1	A	0.31	0/2630	0.55	0/3595
1	B	0.32	0/2641	0.57	0/3612
All	All	0.32	0/5271	0.56	0/7207

There are no bond length outliers.

There are no bond angle outliers.

There are no chirality outliers.

There are no planarity outliers.

5.2 Too-close contacts (i)

In the following table, the Non-H and H(model) columns list the number of non-hydrogen atoms and hydrogen atoms in the chain respectively. The H(added) column lists the number of hydrogen atoms added and optimized by MolProbity. The Clashes column lists the number of clashes within the asymmetric unit, whereas Symm-Clashes lists symmetry-related clashes.

Mol	Chain	Non-H	H(model)	H(added)	Clashes	Symm-Clashes
1	A	2561	0	2423	13	0
1	B	2571	0	2438	9	0
2	A	26	0	36	1	0
2	B	13	0	18	1	0
3	B	7	0	10	1	0
4	B	10	0	14	0	0
5	B	2	0	0	0	0
6	A	221	0	0	3	0
6	B	256	0	0	1	0
All	All	5667	0	4939	21	0

The all-atom clashscore is defined as the number of clashes found per 1000 atoms (including

CONFIDENTIAL VALIDATION REPORT

hydrogen atoms). The all-atom clashscore for this structure is 2.

All (21) close contacts within the same asymmetric unit are listed below, sorted by their clash magnitude.

Atom-1	Atom-2	Interatomic distance (Å)	Clash overlap (Å)
1:B:163:VAL:H	2:B:402:PG4:H52	1.52	0.73
1:B:76:MET:HG2	1:B:143:ILE:HG12	1.81	0.63
1:B:200:GLN:NE2	1:B:202:ALA:H	2.00	0.59
1:A:96:ASN:HB3	1:A:126:VAL:HG11	1.84	0.58
1:A:76:MET:HG2	1:A:143:ILE:HG12	1.86	0.56
1:B:117:LYS:O	1:B:121:THR:HG23	2.10	0.52
1:A:8:GLN:HG2	1:A:12:HIS:O	2.11	0.51
1:A:93:ASP:OD1	1:A:93:ASP:N	2.36	0.50
1:B:200:GLN:HE22	1:B:202:ALA:H	1.57	0.50
1:A:117:LYS:HE3	2:A:402:PG4:H72	1.94	0.50
1:B:96:ASN:HB3	1:B:126:VAL:HG11	1.96	0.47
1:A:158:LYS:NZ	6:A:502:HOH:O	2.28	0.46
1:A:113:ILE:HD12	1:A:152:LEU:HG	1.96	0.46
1:B:117:LYS:NZ	3:B:401:PEG:O4	2.48	0.46
1:A:158:LYS:HG3	6:A:522:HOH:O	2.15	0.46
1:A:193:ARG:NH2	1:B:265:GLU:OE1	2.49	0.45
1:A:273:TYR:CZ	1:A:275:ILE:HD13	2.55	0.42
1:A:8:GLN:HG3	1:A:13:HIS:CD2	2.55	0.42
1:A:42:GLN:NE2	6:A:512:HOH:O	2.50	0.41
1:B:80:ASN:ND2	6:B:504:HOH:O	2.35	0.41
1:A:32:THR:HG21	1:A:38:PHE:CZ	2.56	0.40

There are no symmetry-related clashes.

5.3 Torsion angles (i)

5.3.1 Protein backbone (i)

In the following table, the Percentiles column shows the percent Ramachandran outliers of the chain as a percentile score with respect to all X-ray entries followed by that with respect to entries of similar resolution.

The Analysed column shows the number of residues for which the backbone conformation was analysed, and the total number of residues.

Mol	Chain	Analysed	Favoured	Allowed	Outliers	Percentiles	
1	A	324/333 (97%)	316 (98%)	8 (2%)	0	100	100

Continued on next page...

CONFIDENTIAL VALIDATION REPORT

Continued from previous page...

Mol	Chain	Analysed	Favoured	Allowed	Outliers	Percentiles	
1	B	324/333 (97%)	317 (98%)	7 (2%)	0	100	100
All	All	648/666 (97%)	633 (98%)	15 (2%)	0	100	100

There are no Ramachandran outliers to report.

5.3.2 Protein sidechains (i)

In the following table, the Percentiles column shows the percent sidechain outliers of the chain as a percentile score with respect to all X-ray entries followed by that with respect to entries of similar resolution.

The Analysed column shows the number of residues for which the sidechain conformation was analysed, and the total number of residues.

Mol	Chain	Analysed	Rotameric	Outliers	Percentiles	
1	A	279/292 (96%)	277 (99%)	2 (1%)	84	64
1	B	282/292 (97%)	278 (99%)	4 (1%)	67	37
All	All	561/584 (96%)	555 (99%)	6 (1%)	73	47

All (6) residues with a non-rotameric sidechain are listed below:

Mol	Chain	Res	Type
1	A	17	ARG
1	A	140	HIS
1	B	17	ARG
1	B	67	ASP
1	B	140	HIS
1	B	200	GLN

Sometimes sidechains can be flipped to improve hydrogen bonding and reduce clashes. All (1) such sidechains are listed below:

Mol	Chain	Res	Type
1	B	200	GLN

5.3.3 RNA (i)

There are no RNA molecules in this entry.

CONFIDENTIAL VALIDATION REPORT

5.4 Non-standard residues in protein, DNA, RNA chains (i)

There are no non-standard protein/DNA/RNA residues in this entry.

5.5 Carbohydrates (i)

There are no monosaccharides in this entry.

5.6 Ligand geometry (i)

Of 7 ligands modelled in this entry, 2 are monoatomic - leaving 5 for Mogul analysis.

In the following table, the Counts columns list the number of bonds (or angles) for which Mogul statistics could be retrieved, the number of bonds (or angles) that are observed in the model and the number of bonds (or angles) that are defined in the Chemical Component Dictionary. The Link column lists molecule types, if any, to which the group is linked. The Z score for a bond length (or angle) is the number of standard deviations the observed value is removed from the expected value. A bond length (or angle) with $|Z| > 2$ is considered an outlier worth inspection. RMSZ is the root-mean-square of all Z scores of the bond lengths (or angles).

Mol	Type	Chain	Res	Link	Bond lengths			Bond angles		
					Counts	RMSZ	$\#\|Z\| > 2$	Counts	RMSZ	$\#\|Z\| > 2$
2	PG4	B	402	-	12,12,12	0.52	0	11,11,11	0.32	0
2	PG4	A	402	-	12,12,12	0.52	0	11,11,11	0.17	0
4	PGE	B	403	-	9,9,9	0.32	0	8,8,8	0.28	0
2	PG4	A	401	-	12,12,12	0.51	0	11,11,11	0.25	0
3	PEG	B	401	-	6,6,6	0.48	0	5,5,5	0.24	0

In the following table, the Chirals column lists the number of chiral outliers, the number of chiral centers analysed, the number of these observed in the model and the number defined in the Chemical Component Dictionary. Similar counts are reported in the Torsion and Rings columns. '-' means no outliers of that kind were identified.

Mol	Type	Chain	Res	Link	Chirals	Torsions	Rings
2	PG4	B	402	-	-	8/10/10/10	-
2	PG4	A	402	-	-	6/10/10/10	-
4	PGE	B	403	-	-	0/7/7/7	-
2	PG4	A	401	-	-	5/10/10/10	-
3	PEG	B	401	-	-	0/4/4/4	-

There are no bond length outliers.

There are no bond angle outliers.

CONFIDENTIAL VALIDATION REPORT

There are no chirality outliers.

All (19) torsion outliers are listed below:

Mol	Chain	Res	Type	Atoms
2	A	402	PG4	O2-C3-C4-O3
2	A	401	PG4	C6-C5-O3-C4
2	A	402	PG4	O1-C1-C2-O2
2	B	402	PG4	O4-C7-C8-O5
2	A	402	PG4	O4-C7-C8-O5
2	B	402	PG4	C3-C4-O3-C5
2	A	401	PG4	O3-C5-C6-O4
2	B	402	PG4	O1-C1-C2-O2
2	A	401	PG4	C8-C7-O4-C6
2	A	402	PG4	C8-C7-O4-C6
2	A	401	PG4	C4-C3-O2-C2
2	A	402	PG4	C3-C4-O3-C5
2	A	401	PG4	O4-C7-C8-O5
2	B	402	PG4	C8-C7-O4-C6
2	B	402	PG4	C1-C2-O2-C3
2	A	402	PG4	O3-C5-C6-O4
2	B	402	PG4	O2-C3-C4-O3
2	B	402	PG4	C5-C6-O4-C7
2	B	402	PG4	O3-C5-C6-O4

There are no ring outliers.

3 monomers are involved in 3 short contacts:

Mol	Chain	Res	Type	Clashes	Symm-Clashes
2	B	402	PG4	1	0
2	A	402	PG4	1	0
3	B	401	PEG	1	0

5.7 Other polymers (i)

There are no such residues in this entry.

5.8 Polymer linkage issues (i)

There are no chain breaks in this entry.

CONFIDENTIAL VALIDATION REPORT

6 Fit of model and data (i)

6.1 Protein, DNA and RNA chains (i)

In the following table, the column labelled '#RSRZ> 2' contains the number (and percentage) of RSRZ outliers, followed by percent RSRZ outliers for the chain as percentile scores relative to all X-ray entries and entries of similar resolution. The OWAB column contains the minimum, median, 95th percentile and maximum values of the occupancy-weighted average B-factor per residue. The column labelled 'Q< 0.9' lists the number of (and percentage) of residues with an average occupancy less than 0.9.

Mol	Chain	Analysed	<RSRZ>	#RSRZ>2			OWAB(Å^2)	Q<0.9
1	A	326/333 (97%)	-0.13	15 (4%)	32	33	16, 24, 39, 51	0
1	B	326/333 (97%)	-0.45	2 (0%)	89	90	15, 21, 32, 37	0
All	All	652/666 (97%)	-0.29	17 (2%)	56	56	15, 22, 36, 51	0

All (17) RSRZ outliers are listed below:

Mol	Chain	Res	Type	RSRZ
1	A	92	THR	5.8
1	A	93	ASP	4.0
1	A	10	GLY	3.6
1	A	150	THR	2.9
1	A	91	ALA	2.6
1	A	89	ALA	2.5
1	A	121	THR	2.4
1	A	94	LYS	2.4
1	A	128	ILE	2.4
1	B	150	THR	2.2
1	A	126	VAL	2.2
1	A	118	GLN	2.2
1	A	90	LEU	2.1
1	B	25	TYR	2.1
1	A	43	VAL	2.1
1	A	44	ALA	2.0
1	A	95	ALA	2.0

6.2 Non-standard residues in protein, DNA, RNA chains (i)

There are no non-standard protein/DNA/RNA residues in this entry.

CONFIDENTIAL VALIDATION REPORT

6.3 Carbohydrates (i)

There are no monosaccharides in this entry.

6.4 Ligands (i)

In the following table, the Atoms column lists the number of modelled atoms in the group and the number defined in the chemical component dictionary. The B-factors column lists the minimum, median, 95th percentile and maximum values of B factors of atoms in the group. The column labelled 'Q< 0.9' lists the number of atoms with occupancy less than 0.9.

Mol	Type	Chain	Res	Atoms	RSCC	RSR	B-factors($Å^2$)	Q<0.9
2	PG4	A	401	13/13	0.80	0.30	32,38,41,41	0
2	PG4	A	402	13/13	0.81	0.26	38,42,48,48	0
2	PG4	B	402	13/13	0.87	0.19	25,31,37,42	0
4	PGE	B	403	10/10	0.88	0.10	27,30,33,34	0
3	PEG	B	401	7/7	0.89	0.12	34,36,39,43	0
5	CA	B	404	1/1	0.97	0.04	20,20,20,20	1
5	CA	B	405	1/1	0.98	0.10	43,43,43,43	0

6.5 Other polymers (i)

There are no such residues in this entry.

CONFIDENTIAL VALIDATION REPORT
