# Supplementary material for: Bile salt hydrolases shape the bile acid landscape and restrict Clostridioides difficile growth in the murine gut
Source: Nat Microbiol. 2023 Mar 13;8(4):611–28. doi: 10.1038/s41564-023-01337-7 (PMC10066039; doi:10.1038/s41564-023-01337-7)
Supplement: Supplementary file 3 — Peer Review File [file 41564_2023_1337_MOESM3_ESM.doc]

Peer Review Information

**Journal: Nature Microbiology**

**Manuscript Title:** Bile salt hydrolases shape the bile acid landscape and restrict Clostridioides difficile growth in the murine gut

**Corresponding author name(s): Dr Casey Theriot**

| **Redactions – transferred manuscripts (mention of previous referee reports from elsewhere)** | This manuscript has been previously reviewed at another journal. This document only contains reviewer comments, rebuttal and decision letters for versions considered at Nature. Mentions of prior referee reports have been redacted |
| --- | --- |
|  |  |

**Reviewer Comments & Decisions:**

**Decision Letter, initial version:**

10th January 2023

Dear Casey,

Thank you for your patience as we’ve prepared the guidelines for final submission of your Nature Microbiology manuscript, "Bile salt hydrolases shape the intestinal bile acid landscape and restrict <i>C. difficile</i>" (NMICROBIOL-22112802-T). Please carefully follow the step-by-step instructions provided in the attached file, and add a response in each row of the table to indicate the changes that you have made. Please also check and comment on any additional marked-up edits we have proposed within the text. Ensuring that each point is addressed will help to ensure that your revised manuscript can be swiftly handed over to our production team.

We would like to start working on your revised paper, with all of the requested files and forms, as soon as possible (preferably within two weeks). Please get in contact with us if you anticipate delays.

When you upload your final materials, please include a point-by-point response to any remaining reviewer comments.

If you have not done so already, please alert us to any related manuscripts from your group that are under consideration or in press at other journals, or are being written up for submission to other journals (see: https://www.nature.com/nature-research/editorial-policies/plagiarism#policy-on-duplicate-publication for details).

In recognition of the time and expertise our reviewers provide to Nature Microbiology’s editorial process, we would like to formally acknowledge their contribution to the external peer review of your manuscript entitled "Bile salt hydrolases shape the intestinal bile acid landscape and restrict <i>C. difficile</i>". For those reviewers who give their assent, we will be publishing their names alongside the published article.

Nature Microbiology offers a Transparent Peer Review option for new original research manuscripts submitted after December 1st, 2019. As part of this initiative, we encourage our authors to support increased transparency into the peer review process by agreeing to have the reviewer comments, author rebuttal letters, and editorial decision letters published as a Supplementary item. When you submit your final files please clearly state in your cover letter whether or not you would like to participate in this initiative. Please note that failure to state your preference will result in delays in accepting your manuscript for publication.

**Cover suggestions**

As you prepare your final files we encourage you to consider whether you have any images or illustrations that may be appropriate for use on the cover of Nature Microbiology.

Covers should be both aesthetically appealing and scientifically relevant, and should be supplied at the best quality available. Due to the prominence of these images, we do not generally select images featuring faces, children, text, graphs, schematic drawings, or collages on our covers.

We accept TIFF, JPEG, PNG or PSD file formats (a layered PSD file would be ideal), and the image should be at least 300ppi resolution (preferably 600-1200 ppi), in CMYK colour mode.

If your image is selected, we may also use it on the journal website as a banner image, and may need to make artistic alterations to fit our journal style.

Please submit your suggestions, clearly labeled, along with your final files. We’ll be in touch if more information is needed.


Nature Microbiology has now transitioned to a unified Rights Collection system which will allow our Author Services team to quickly and easily collect the rights and permissions required to publish your work. Approximately 10 days after your paper is formally accepted, you will receive an email in providing you with a link to complete the grant of rights. If your paper is eligible for Open Access, our Author Services team will also be in touch regarding any additional information that may be required to arrange payment for your article.

Please note that you will not receive your proofs until the publishing agreement has been received through our system.

Please note that <i>Nature Microbiology</i> is a Transformative Journal (TJ). Authors may publish their research with us through the traditional subscription access route or make their paper immediately open access through payment of an article-processing charge (APC). Authors will not be required to make a final decision about access to their article until it has been accepted. <a href="https://www.springernature.com/gp/open-research/transformative-journals"> Find out more about Transformative Journals</a>

**Authors may need to take specific actions to achieve <a href="https://www.springernature.com/gp/open-research/funding/policy-compliance-faqs"> compliance</a> with funder and institutional open access mandates.** If your research is supported by a funder that requires immediate open access (e.g. according to <a href="https://www.springernature.com/gp/open-research/plan-s-compliance">Plan S principles</a>) then you should select the gold OA route, and we will direct you to the compliant route where possible. For authors selecting the subscription publication route, the journal’s standard licensing terms will need to be accepted, including <a href="https://www.nature.com/nature-portfolio/editorial-policies/self-archiving-and-license-to-publish">self-archiving policies</a>. Those licensing terms will supersede any other terms that the author or any third party may assert apply to any version of the manuscript.


For information regarding our different publishing models please see our <a href="https://www.springernature.com/gp/open-research/transformative-journals"> Transformative Journals </a> page. If you have any questions about costs, Open Access requirements, or our legal forms, please contact ASJournals@springernature.com.

[redacted]

If you have any further questions, please feel free to contact me.


[redacted]


Reviewer #1:
Remarks to the Author:


The authors have been responsive to the reviewers’ comments and the results presented in this study are novel. Bile acids modulate many aspects of the gut ecosystem and host health. The discovery of residues important for BSH specificity and the impact this may have on the BA pool is foundational and represents a significant contribution to the gut microbiome field, beyond C.difficile.

However, as presented there is a disconnect between the very exciting sequence structure-fuction work and the Cdiff studies where the focus is on the overall effect of BSH on Cdiff colonization without a clear connection to BSH specificity.

I think what it is missing is evidence showing that the BSH deconjugation specificity nicely demonstrated in vitro has implications for BA homeostasis in vivo. The ex vivo experiments are not conclusive on this, possibly due to complexity/nature of the human samples used. Also the mouse experiment presented in figure 5 lacks a control in which a BSH enzyme specific for glycine-conjugated BA is supplemented. If specificity is important in vivo I would expect to see that it would have a much smaller effect in the pool of unconjugated BA in the mouse gut where G-conjugated BA are a minor proportion, and potentially no effect of Cdiff colonization levels.

Other comments

a. Clinical data for Cdiff mouse experiment should be at least shown as suppl data
b. Not clear what is the replication of experiment 5G. Is the lag phase observed after several passage with Tyr-βMCA and Phe-βMCA ?

c. On the sentence: “Our results suggest that leveraging the BA metabolic functions of the microbiota to limit C. difficile colonization may present an alternative strategy that avoids antibiotic damage, the unspecified risks of FMTs, and the variability inherent to live biotherapeutic products.”

(i) Impact of BSH on the microbiome is not described. Are there other taxa besides Cdiff impacted by this treatment?
(ii) Looks that variability is high at least based on the ex vivo study

d. For both ex vivo and in vivo assays: it is great that unconjugated BA increase, suggesting that supplied BSH are active in the conditions tested; however no data is provided in terms of % activity from what would be expected based on the amount of enzyme supplied. For example effects are a lot smaller in the cecum. It is not clear whether supplied BSH gets there. Are BSH intrinsically resistant to proteases?

e. Fig 5B. Mann-Whitney (non-parametric test) may be better test to use than Welch’s t-test

f. lines 246-248: “conjugated BAs may suppress the expression of C. difficile’s toxin A……….” – what is the implication of this finding?


Reviewer #2:
Remarks to the Author:
The manuscript by Dr. Theriot et al. is an important contribution to the fields of microbiome, bile acid, and C. difficile research. I am confident this manuscript will be of broad interest to a variety of researchers across a range of disciplines. The manuscript is an interesting combination of structural and computational biology which with strong microbiology implications as it pertains to community function and C. difficile infection.

I have done my best to summarize the key concerns of each reviewer, the responses, and my interpretation; however, I think the authors have sufficiently addressed the previous comments and the manuscript as it stands has no fatal flaws that should prevent its publication in Nature Microbiology.

Reviewer #1

Concern 1: Lack of substrate specificity being determined by SRX vs GXG motif.
Response: Additional data and figures including perhaps most importantly panels in Figure 2 including panel F.

I think between the heterologous expression data (2F) and the mutants (2IK, 2JK) I am convinced of the significance of these motifs, at least in Lactobacillaceae BSHs.

Concern 2: Variable results and confounding inhibitory activity in ex vivo incubations performed with BSH-treated fecal extracts.
Response: These samples come from real humans with complex unobserved variables. Untargeted metabolomics data detected several antibiotics including vancomycin in the feces of recipients 4-6.

This variability does not surprise me in the least, in fact, I am almost surprised the experiment worked as well as it did. I think it serves as a useful proof of concept; however, more definitive evidence would likely be time/cost prohibitive. The detection of antimicrobials in Fig S8 Is useful, but the units of peak area do not lend themselves well to knowing if there was an inhibitory concentration of antimicrobial present. The bile acid profiles of subjects 1-3 are quite different than 4-6 and It seems somewhat odd that the authors did not quantify LCA, but I think that might be splitting hairs.

Concern 3: Animal model did not show effect on gross disease activity and reduction in C. difficile colonization is ~ 1-2 log.
Response: Model is intended to understand prevention rather than treatment and C. difficile load is appropriate way to address that question. Examples of published literature with similar changes.

I think the data is sufficiently compelling and can confirm from my own work that many microbiome directed interventions have similar effect sizes on C. difficile abundance. I think it would probably be possible to revise the model to find a gross effect on clinical signs, but that may be secondary to the main point of the manuscript.
Reviewer #2

Concern 1: Concerns about experiment replication in Figure 5 and how statistics were applied.
Response: Clarification of statistical treatment, reporting summary modifications.

I would propose that the authors should clearly identify that the data is pooled from multiple experiments in the legend of Figure 5 and explicitly identify the number of mice used and per experiment. I think they addressed the plotting discrepancy of missing dots adequately; however, it wasn’t clear how these ND values were treated in the statistical treatment. The authors should consider clarifying how they normalized/filled data before their statistical testing in the methods. The figure legend still makes reference to a one-tailed t-test for 5B. I think the ANOVA with Dunnett’s multiple comparison is suitable; however, I would encourage the authors to consider non-parametric statistical tests (mann whitney/ kruskal wallis) going forward as they would probably better fit the nature of the data and not be impacted by data normalization/psuedocounting not detected values.

Concern 2: Not identifying limitations adequately and overstating/misrepresentation of the results.
Response: additional discussion paragraph about limitations and textual changes for accuracy.

I think the response was appropriate.

Concern 3: Detection of GCA in mice where taurine conjugates are predominant.
Response: Clarification with references.

I am also aware of the presence of glycine conjugates in mice.

Concern 4: Absolutel quantification of MCBAs not performed as no authentic standards available but pure MCBAs used in experiments.
Response: None are available commercially with labels.

While the labelled MCBAs would be preferable as an internal standard for quantification, I am not aware of why a labelled version is absolutely required to generate a standard curve. Either way, I do not think this is a critical detail and to go back to do absolute quantification would be time and resource intensive.

Concern 5: Claim of 3-residue motif being generalizable weak.
Response: additional data as previously identified and text changes.

I think the modifications and text changes are sufficient.

Concern 6: authors do not see increase in DCA/LCA.
Response: Clarifications about LCA/DCA from literature.

I do not view this as a significant issue, and as the authors identify, it is more interesting to me that they are not increased.

Reviewer #3

Concern 1: motifs do not determine specificity and there are other structural considerations which have been ignored.
Response: pointing out experiments in figure 2, particularly the mutagenesis. Supplementary data as well relating to structure calculations.

I’m not sure about the chronology of the manuscript version I have versus these comments, but I think the data is sufficiently compelling.

Concern 2: novelty of Figure 3AB and variability of Fig 3EF.
Response: Pointing towards response to previous reviewer for EF and does not address AB.

I think there is some validity to criticizing the novelty of Fig 3AB, but these are important data none the less. Worst case scenario these could be moved to supplemental data. As previously discussed, the data from the 6 humans is sufficient in my opinion as a proof of concept.

Concern 3: novelty of MCBA formation in small intestine, lack of absolute bile acid quantification.
Response: Respectfully disagree re novelty, and point to previous reviewer response re: isotope labelled standards. Add limitation relating to lack of absolute quantification to discussion.

I would agree with the authors that MCBA formation is quite novel and as before, do not view the lack of absolutely quantification to be a major downfall of the manuscript.

Concern 4: MCBA concentrations not physiologically relevant.
Response: within range previously assayed by other groups.

The concentrations are not outrageous and we do not yet know the true physiologically relevant concentrations.

Concern 5: Lack of disease outcomes from animal model.
Response: as before for reviewer #1 concern #3.

Concern 6: Not established that BSH produces MCBAs.
Response: supported by two preprints (presumably in review).

I think this would be an editorial decision, but given it has been replicated in at least two different groups, it is likely safe to include; however, perhaps the language should be softened slightly.

Concern 7: tenuous link between structural biology and C. difficile work.
Response: text modifications to make link clearer

I think there is some validity to the reviewer’s comment; however, I don’t think this is sufficient to impede publication as both elements on their own are important contributions.

**Author Rebuttal to Initial comments**

**We thank the reviewers again for their thoughtful and positive comments. All of our responses to reviewer comments are below in blue bold font.**

Reviewer #1 (Remarks to the Author):

The authors have been responsive to the reviewers’ comments and the results presented in this study are novel. Bile acids modulate many aspects of the gut ecosystem and host health. The discovery of residues important for BSH specificity and the impact this may have on the BA pool is foundational and represents a significant contribution to the gut microbiome field, beyond C. difficile.  

However, as presented there is a disconnect between the very exciting sequence structure-fuction work and the Cdiff studies where the focus is on the overall effect of BSH on Cdiff colonization without a clear connection to BSH specificity. 

I think what it is missing is evidence showing that the BSH deconjugation specificity nicely demonstrated in vitro has implications for BA homeostasis in vivo. The ex vivo experiments are not conclusive on this, possibly due to complexity/nature of the human samples used. Also the mouse experiment presented in figure 5 lacks a control in which a BSH enzyme specific for glycine-conjugated BA is supplemented. If specificity is important in vivo I would expect to see that it would have a much smaller effect in the pool of unconjugated BA in the mouse gut where G-conjugated BA are a minor proportion, and potentially no effect of Cdiff colonization levels. 
**We thank the reviewer for their overall positive reception of our manuscript and for we enthusiastically agree that the implications for BSH activity in the gut extend outside of just *C. difficile*.**

**Testing the role of BSH specificity is a challenge without the appropriate models containing equal parts glycine and taurine conjugations to clearly answer this question. As the reviewer pointed out, the heterogeneity and complexity of human samples was not as conclusive as we hoped it would be. Nevertheless, it is reasonable to assume BSH substrate preferences in vitro reflect their capacity to act in vivo when in limited concentrations.**

**Taurine-preferring BSHs were used in vivo to match the taurine-skewed murine BA pool and they were delivered in excess to overcome gastric passage and degradation. Glycine-preferring BSHs, which do still have measurable taurine activity, would likely still work on the murine BA pool if also delivered in excess. Overall, we don’t believe that carefully titrating and dosing glycine-preferring BSHs to establish that they have a smaller effect in mice is within the scope of this study since our motivation was establish the impact of deconjugation and MCBA production on *C. difficile in vivo*.**

**We have added this statement to address this reviewer comment and also this limitation of this study:**

*“The importance of BSH substrate preferences is most relevant to the human BA pool where glycine and taurine-conjugated BAs dominate. The mouse remains a powerful model for studying BA dynamics in vivo but its taurine bias is limiting, especially as it related to BSH substrate preferences.”*

Other comments

a. Clinical data for Cdiff mouse experiment should be at least shown as suppl data

**We have now included a new Figure S7 showing the differences in weight loss during the CDI mouse model, that reflects the clinical signs of disease.**

b. Not clear what is the replication of experiment 5G. Is the lag phase observed after several passage with Tyr-βMCA and Phe-βMCA ?

**We have now separately colored the data points from our experimental Figure 5B.**

**This reviewer’s question about the lag phase in Figure 5G is a valid one, but we believe that investigating these specific phenotypes on the growth kinetics of *C. difficile* is out of the scope of this manuscript and will be focused on mechanistically in future studies.**

c. On the sentence: “Our results suggest that leveraging the BA metabolic functions of the microbiota to limit C. difficile colonization may present an alternative strategy that avoids antibiotic damage, the unspecified risks of FMTs, and the variability inherent to live biotherapeutic products.”
(i) Impact of BSH on the microbiome is not described. Are there other taxa besides Cdiff impacted by this treatment?

**Indeed it looks like there are other bacteria that are affected by MCBAs as seen in Rob Quinn’s most recent preprint here:**

<https://assets.researchsquare.com/files/rs-2050406/v1/16cc2659-e7b6-47a7-918f-2e24919dd695.pdf?c=1671066357>

**But, we did not focus on how they affected other members of the gut microbiota as our question was specific to how they affected *C. difficile* for this paper. Moving beyond this study it will be important to understand how BSHs affect the gut microbiota, but it will be challenging to parse out if it is the BSHs and or the bile acids they produce that are really driving the structural changes.**

**The cefoperazone mouse model is reproducible in that the predominant bacterial family member present after antibiotic treatment is the Enterococcaceae. Then when *C. difficile* is introduced it is able to colonize the gut. Other members of the gut microbiota do not start returning in this model until beyond day 2 post challenge, which was our clinical endpoint for this study. To address how these BSHs or rather the BAs they produce affect the returning gut microbiota the experiments need to be done for a longer duration. Additionally, many controls would need to be in place to actually understand how the BSHs or rather their BA products are able to shape the gut microbiota. At this point it is outside the scope of this paper, but something we are pursuing.**

(ii) Looks that variability is high at least based on the ex vivo study
**We agree there is inherent variability, likely reflecting the dynamics of the bile acid pool and patient sample variation demonstrated in Figure 3E, F and highlight this as a limitation of the study.**

d. For both ex vivo and in vivo assays: it is great that unconjugated BA increase, suggesting that supplied BSH are active in the conditions tested; however no data is provided in terms of % activity from what would be expected based on the amount of enzyme supplied. For example effects are a lot smaller in the cecum. It is not clear whether supplied BSH gets there. Are BSH intrinsically resistant to proteases?  
**We appreciate the reviewers comment here. The suggestion of calculating percent activity is challenging and cannot be extrapolated from the data we’ve collected. Since BSH activity in these complex samples results in further transformations, i.e. secondary bile acid transformations and reconjugation, calculating the percent of a given bile acids deconjugation is not doable without being able to track these other transformations as well.**

**The reduced effect of *C. difficile* inhibition in the cecum is likely attributed to the overall lower concentration of bile acids present there. It is not clear from our data if our delivered BSHs are present and active in the cecum since their activity in the small intestine is likely impacting the downstream bile acid pool of the large intestine. We do not know the status of our BSHs in the cecum and while their presence could be measured, quantifying their remaining activity or susceptibility to intestinal proteases would be a challenge.**

e. Fig 5B. Mann-Whitney (non-parametric test) may be better test to use than Welch’s t-test.
**We thank the reviewer for this suggestion. The one-tailed Mann-Whitney was performed and it does not change the level of statistical significance.**

f. lines 246-248: “conjugated BAs may suppress the expression of C. difficile’s toxin A……….” – what is the implication of this finding?
**We have now included additional explanation for the implication of these results:**

*“C. difficile toxins mediate the severity of CDI and their decreased expression may help ameliorate disease”*

Reviewer #2 (Remarks to the Author):

The manuscript by Dr. Theriot et al. is an important contribution to the fields of microbiome, bile acid, and C. difficile research. I am confident this manuscript will be of broad interest to a variety of researchers across a range of disciplines. The manuscript is an interesting combination of structural and computational biology which with strong microbiology implications as it pertains to community function and C. difficile infection.

I have done my best to summarize the key concerns of each reviewer, the responses, and my interpretation; however, I think the authors have sufficiently addressed the previous comments and the manuscript as it stands has no fatal flaws that should prevent its publication in Nature Microbiology.

**We thank the reviewer for their support and appreciation. We have addressed the comments below where necessary.**

Reviewer #1

Concern 1: Lack of substrate specificity being determined by SRX vs GXG motif. 
Response: Additional data and figures including perhaps most importantly panels in Figure 2 including panel F.

I think between the heterologous expression data (2F) and the mutants (2IK, 2JK) I am convinced of the significance of these motifs, at least in Lactobacillaceae BSHs.

**We agree with the reviewer that our data is most convincing in Lactobacillaceae BSHs and likely applies to other families as well.**

Concern 2: Variable results and confounding inhibitory activity in ex vivo incubations performed with BSH-treated fecal extracts.
Response: These samples come from real humans with complex unobserved variables. Untargeted metabolomics data detected several antibiotics including vancomycin in the feces of recipients 4-6.

This variability does not surprise me in the least, in fact, I am almost surprised the experiment worked as well as it did. I think it serves as a useful proof of concept; however, more definitive evidence would likely be time/cost prohibitive. The detection of antimicrobials in Fig S8 Is useful, but the units of peak area do not lend themselves well to knowing if there was an inhibitory concentration of antimicrobial present. The bile acid profiles of subjects 1-3 are quite different than 4-6 and It seems somewhat odd that the authors did not quantify LCA, but I think that might be splitting hairs.
**We too were surprised by the inhibition we saw. LCA was measured, but was not detected in these samples, likely since these patients were previously or concurrently on antibiotics that are known to diminish secondary bile acid production.**

Concern 3: Animal model did not show effect on gross disease activity and reduction in C. difficile colonization is ~ 1-2 log.
Response: Model is intended to understand prevention rather than treatment and C. difficile load is appropriate way to address that question. Examples of published literature with similar changes.

I think the data is sufficiently compelling and can confirm from my own work that many microbiome directed interventions have similar effect sizes on C. difficile abundance. I think it would probably be possible to revise the model to find a gross effect on clinical signs, but that may be secondary to the main point of the manuscript.
**We agree that we would likely need to investigate this further to find possible differences in clinical signs of disease. As stated by the reviewer, this is beyond the scope of this paper as our question focused on colonization.**

Reviewer #2

Concern 1: Concerns about experiment replication in Figure 5 and how statistics were applied.
Response: Clarification of statistical treatment, reporting summary modifications.

I would propose that the authors should clearly identify that the data is pooled from multiple experiments in the legend of Figure 5 and explicitly identify the number of mice used and per experiment. I think they addressed the plotting discrepancy of missing dots adequately; however, it wasn’t clear how these ND values were treated in the statistical treatment. The authors should consider clarifying how they normalized/filled data before their statistical testing in the methods. The figure legend still makes reference to a one-tailed t-test for 5B. I think the ANOVA with Dunnett’s multiple comparison is suitable; however, I would encourage the authors to consider non-parametric statistical tests (mann whitney/ kruskal wallis) going forward as they would probably better fit the nature of the data and not be impacted by data normalization/psuedocounting not detected values. 
**We have now color-coded the data in Figure 5B to help visualize the independent experiments separately. Given this comment and the request of Reviewer 1, we have now analyzed the data in Fig. 5B using a one-tailed Mann-Whitney test and the data in Fig. 5C using a Kruskal-Wallis test which we agree works best with these data. Figure 5C ND values were treated as 0 in the statistics as they were calculated to fall below limit of detection relative to internal standards.**

Concern 2: Not identifying limitations adequately and overstating/misrepresentation of the results.
Response: additional discussion paragraph about limitations and textual changes for accuracy.

I think the response was appropriate.

Concern 3: Detection of GCA in mice where taurine conjugates are predominant.
Response: Clarification with references.

I am also aware of the presence of glycine conjugates in mice.

Concern 4: Absolutel quantification of MCBAs not performed as no authentic standards available but pure MCBAs used in experiments.
Response: None are available commercially with labels. 

While the labelled MCBAs would be preferable as an internal standard for quantification, I am not aware of why a labelled version is absolutely required to generate a standard curve. Either way, I do not think this is a critical detail and to go back to do absolute quantification would be time and resource intensive.

Concern 5: Claim of 3-residue motif being generalizable weak.
Response: additional data as previously identified and text changes.

I think the modifications and text changes are sufficient.

Concern 6: authors do not see increase in DCA/LCA.
Response: Clarifications about LCA/DCA from literature. 

I do not view this as a significant issue, and as the authors identify, it is more interesting to me that they are not increased.

Reviewer #3

Concern 1: motifs do not determine specificity and there are other structural considerations which have been ignored.
Response: pointing out experiments in figure 2, particularly the mutagenesis. Supplementary data as well relating to structure calculations.

I’m not sure about the chronology of the manuscript version I have versus these comments, but I think the data is sufficiently compelling.

Concern 2: novelty of Figure 3AB and variability of Fig 3EF.
Response: Pointing towards response to previous reviewer for EF and does not address AB.

I think there is some validity to criticizing the novelty of Fig 3AB, but these are important data none the less. Worst case scenario these could be moved to supplemental data. As previously discussed, the data from the 6 humans is sufficient in my opinion as a proof of concept.

Concern 3: novelty of MCBA formation in small intestine, lack of absolute bile acid quantification.
Response: Respectfully disagree re novelty, and point to previous reviewer response re: isotope labelled standards. Add limitation relating to lack of absolute quantification to discussion.

I would agree with the authors that MCBA formation is quite novel and as before, do not view the lack of absolutely quantification to be a major downfall of the manuscript.

Concern 4: MCBA concentrations not physiologically relevant.
Response: within range previously assayed by other groups.

The concentrations are not outrageous and we do not yet know the true physiologically relevant concentrations.

Concern 5: Lack of disease outcomes from animal model.
Response: as before for reviewer #1 concern #3.

Concern 6: Not established that BSH produces MCBAs. 
Response: supported by two preprints (presumably in review).

I think this would be an editorial decision, but given it has been replicated in at least two different groups, it is likely safe to include; however, perhaps the language should be softened slightly.
**We thank the reviewer for their comment and have softened the language accordingly:**

*”Additionally, these data suggest that BSHs are involved in MCBA production; indeed, other studies are already investigating the possibility of BSH-catalyzed reconjugation.”*

Concern 7: tenuous link between structural biology and C. difficile work.
Response: text modifications to make link clearer

I think there is some validity to the reviewer’s comment; however, I don’t think this is sufficient to impede publication as both elements on their own are important contributions.

**Final Decision Letter:**

8th February 2023

Dear Casey,

I am pleased to accept your Article "Bile salt hydrolases shape the bile acid landscape and restrict <i>Clostridioides difficile</i> growth in the murine gut" for publication in Nature Microbiology. Thank you for having chosen to submit your work to us and many congratulations!

Over the next few weeks, your paper will be copyedited to ensure that it conforms to Nature Microbiology style. We look particularly carefully at the titles of all papers to ensure that they are relatively brief and understandable.

Once your paper is typeset, you will receive an email with a link to choose the appropriate publishing options for your paper and our Author Services team will be in touch regarding any additional information that may be required. Once your paper has been scheduled for online publication, the Nature press office will be in touch to confirm the details.

After the grant of rights is completed, you will receive a link to your electronic proof via email with a request to make any corrections within 48 hours. If, when you receive your proof, you cannot meet this deadline, please inform us at rjsproduction@springernature.com immediately. You will not receive your proofs until the publishing agreement has been received through our system

Due to the importance of these deadlines, we ask you please us know now whether you will be difficult to contact over the next month. If this is the case, we ask you provide us with the contact information (email, phone and fax) of someone who will be able to check the proofs on your behalf, and who will be available to address any last-minute problems.

Acceptance of your manuscript is conditional on all authors' agreement with our publication policies (see https://www.nature.com/nmicrobiol/editorial-policies). In particular your manuscript must not be published elsewhere and there must be no announcement of the work to any media outlet until the publication date (the day on which it is uploaded onto our website).

Please note that <i>Nature Microbiology</i> is a Transformative Journal (TJ). Authors may publish their research with us through the traditional subscription access route or make their paper immediately open access through payment of an article-processing charge (APC). Authors will not be required to make a final decision about access to their article until it has been accepted. <a href="https://www.springernature.com/gp/open-research/transformative-journals"> Find out more about Transformative Journals</a>

**Authors may need to take specific actions to achieve <a href="https://www.springernature.com/gp/open-research/funding/policy-compliance-faqs"> compliance</a> with funder and institutional open access mandates.** If your research is supported by a funder that requires immediate open access (e.g. according to <a href="https://www.springernature.com/gp/open-research/plan-s-compliance">Plan S principles</a>) then you should select the gold OA route, and we will direct you to the compliant route where possible. For authors selecting the subscription publication route, the journal’s standard licensing terms will need to be accepted, including <a href="https://www.nature.com/nature-portfolio/editorial-policies/self-archiving-and-license-to-publish">self-archiving policies</a>. Those licensing terms will supersede any other terms that the author or any third party may assert apply to any version of the manuscript.


If you have any questions about our publishing options, costs, Open Access requirements, or our legal forms, please contact ASJournals@springernature.com

An online order form for reprints of your paper is available at <a href="https://www.nature.com/reprints/author-reprints.html">https://www.nature.com/reprints/author-reprints.html</a>. All co-authors, authors' institutions and authors' funding agencies can order reprints using the form appropriate to their geographical region.

We welcome the submission of potential cover material (including a short caption of around 40 words) related to your manuscript; suggestions should be sent to Nature Microbiology as electronic files (the image should be 300 dpi at 210 x 297 mm in either TIFF or JPEG format). Please note that such pictures should be selected more for their aesthetic appeal than for their scientific content, and that colour images work better than black and white or grayscale images. Please do not try to design a cover with the Nature Microbiology logo etc., and please do not submit composites of images related to your work. I am sure you will understand that we cannot make any promise as to whether any of your suggestions might be selected for the cover of the journal.

You can now use a single sign-on for all your accounts, view the status of all your manuscript submissions and reviews, access usage statistics for your published articles and download a record of your refereeing activity for the Nature journals.

To assist our authors in disseminating their research to the broader community, our SharedIt initiative provides you with a unique shareable link that will allow anyone (with or without a subscription) to read the published article. Recipients of the link with a subscription will also be able to download and print the PDF.


As soon as your article is published, you will receive an automated email with your shareable link.


With kind regards,

[redacted]
